# TBX3 Regulates Splicing *In Vivo*: A Novel Molecular Mechanism for Ulnar-Mammary Syndrome

**DOI:** 10.1371/journal.pgen.1004247

**Published:** 2014-03-27

**Authors:** Pavan Kumar P., Sarah Franklin, Uchenna Emechebe, Hao Hu, Barry Moore, Chris Lehman, Mark Yandell, Anne M. Moon

**Affiliations:** 1 Department of Pediatrics, University of Utah, Salt Lake City, Utah, United States of America; 2 Department of Internal Medicine and the Nora Eccles Harrison Cardiovascular Research & Training Institute, University of Utah, Salt Lake City, Utah, United States of America; 3 Department of Anesthesiology, University of California Los Angeles, Los Angeles, California, United States of America; 4 Department of Neurobiology and Anatomy, University of Utah, Salt Lake City, Utah, United States of America; 5 Department of Human Genetics, University of Utah, Salt Lake City, Utah, United States of America; 6 Molecular Medicine Program, University of Utah, Salt Lake City, Utah, United States of America; 7 Weis Center for Research, Geisinger Clinic, Danville, Pennsylvania, United States of America; Stanford University School of Medicine, United States of America

## Abstract

TBX3 is a member of the T-box family of transcription factors with critical roles in development, oncogenesis, cell fate, and tissue homeostasis. *TBX3* mutations in humans cause complex congenital malformations and Ulnar-mammary syndrome. Previous investigations into TBX3 function focused on its activity as a transcriptional repressor. We used an unbiased proteomic approach to identify TBX3 interacting proteins *in vivo* and discovered that TBX3 interacts with multiple mRNA splicing factors and RNA metabolic proteins. We discovered that TBX3 regulates alternative splicing *in vivo* and can promote or inhibit splicing depending on context and transcript. TBX3 associates with alternatively spliced mRNAs and binds RNA directly. TBX3 binds RNAs containing TBX binding motifs, and these motifs are required for regulation of splicing. Our study reveals that *TBX3* mutations seen in humans with UMS disrupt its splicing regulatory function. The pleiotropic effects of *TBX3* mutations in humans and mice likely result from disrupting at least two molecular functions of this protein: transcriptional regulation and pre-mRNA splicing.

## Author Summary

TBX3 is a protein with essential roles in development and tissue homeostasis, and is implicated in cancer pathogenesis. *TBX3* mutations in humans cause a complex of birth defects called Ulnar-mammary syndrome (UMS). Despite the importance of TBX3 and decades of investigation, few TBX3 partner proteins have been identified and little is known about how it functions in cells. Unlike previous investigations focused on TBX3 as DNA binding factor that represses transcription, we took an unbiased approach to identify TBX3 partner proteins in mouse embryos and human cells. We discovered that TBX3 interacts with RNA binding proteins and binds mRNAs to regulate how they are spliced. The different mutations seen in human UMS patients produce mutant proteins that interact with different partners and have different splicing activities. TBX3 promotes or inhibits splicing depending on cellular context, its partner proteins, and the target mRNA. Eukaryotic cells have many more proteins than genes: alternative splicing is critical to generate the different mRNAs needed for production of the specific and vast repertoire of proteins a cell produces. Our finding that TBX3 regulates this process provides fundamental new insights into how altered quantity and molecular function of TBX3 contribute to human developmental disorders and cancer.

## Introduction

TBX3 belongs to the T-box family of transcription factors. The crucial roles of TBX3 in development are evident in the fact that heterozygous mutations of *TBX3* cause Ulnar–mammary syndrome in humans (UMS). This syndrome includes limb malformations, apocrine and mammary gland hypoplasia, dental and genital abnormalities. Altered *TBX3* expression is implicated in the pathogenesis of breast and other cancers by affecting cell adhesion, proliferation and senescence [1], [2], [3], [4], [5], [6]. Tbx3 improves germ line competence of iPS cells [7] and can reprogram mature cardiomyocytes [8]. In addition to its roles in limb and mammary development, Tbx3 is required for formation and homeostasis of the cardiac conduction system [9], [10] and plays a role in cardiac development and function in humans [11], [12], [13], [14].

The DNA-binding domain (DBD) that TBX3 shares with other T-box family members is critical for its function since missense mutations in this domain cause UMS. In addition to binding consensus T-box binding elements (TBEs), TBX3 contains a C-terminal dominant repressor domain [15]. Investigations into TBX3 molecular functions have focused on transcriptional effects including repression of p19^ARF^ and p21 to regulate cell proliferation and prevent apoptosis [3], [6], [16], [17]. Interactions with histone deacetylases and co-repressors mediate at least some TBX3 repressor function [5], [16], [18], [19], [20]. Beyond these studies, few direct transcriptional targets or interacting proteins have been identified.

Given the importance of TBX3 in development, human disease, and its potential as therapeutic target for cancer and tissue regeneration, it is essential to define the pathways in which it functions. To obtain new insights into the molecular functions of TBX3/Tbx3 (TBX3 = human; Tbx3 = mouse), we interrogated interacting partners *in vivo*. Based on previous studies, we expected to identify transcription factors and chromatin modifying proteins. Remarkably, we discovered a novel function of TBX3 with direct relevance to understanding the molecular mechanisms of mutations that cause human disease.

## Results

### Mass spectrometry analyses of TBX3 co-immunoprecipitated proteins identify novel interacting proteins

**Figure 1:**
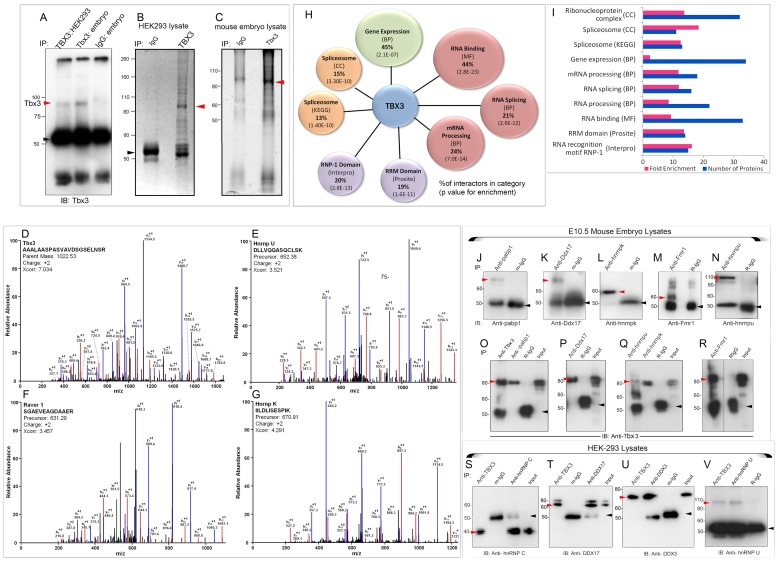
Splicing factors and RNA BPs are over-represented in the Tbx3 interactome identified by MS and are validated co-immunoprecipitation. A) Immunoblot (IB) detecting endogenous TBX3/Tbx3 protein immunoprecipitated (IP) from HEK293 and mouse embryo lysates, respectively. black arrowhead: IgG. B, C) Representative, Coomassie (B, 4–12% gradient) and Oriole stained (C, 10%) SDS-PAGE gels of anti-Tbx3 or negative control IgG IP'd complexes subsequently analyzed by MS. Red arrowheads, TBX3/Tbx3. D–G) Representative mass spectra for identification of Tbx3 (D) and interacting proteins: Hnrnp U (E), Raver 1 (F), Hnrnp K (G). Diagnostic b- and y-series ions are shown in red and blue. H) Gene ontology (GO) analysis identified predominant molecular functions and biological processes of Tbx3 interacting proteins. Diagram shows percent interacting proteins annotated with each GO term (molecular function, MF; biological process, BP; cellular component, CC; functional domain (Interpro and Prosite) and molecular pathway (KEGG). I) Bar graph of number of proteins and fold enrichment per category. J–V) Mouse embryo and HEK293 lysates were immunoprecipitated with the antibody listed at the top of the panels (IP) and the antibodies used to probe the immunoblot (IB) below. J–N) Immunoprecipitation of endogenous: pabp1, Ddx17, hnrnp k, Fmr1, hnrnp u from e10.5 mouse embryo lysates. red arrowheads: interacting protein; black arrowheads: IgG O–R) Endogenous Tbx3 coIPs with pabp1 (O), Ddx17 (P), hnrnp k and hnrnp u (Q), Fmr1 (R) in e10.5 mouse embryos. S–V) Endogenous TBX3 coIPs with hnRNP C (S), DDX17 (T), DDX3 (U) and hnRNP U (V) in HEK293 cells. Red arrowheads: interacting protein; black arrowheads: IgG.

We employed unbiased proteomic screens using a custom polyclonal antibody generated against a peptide from the C-terminus of TBX3 [21]. We immunoprecipitated (IP'd) protein lysates from embryonic day (e)10.5 mouse embryos and from the human embryonic kidney cell line, HEK293 (Figure 1A) to isolate Tbx3 and TBX3 interacting proteins, respectively. IP'd proteins were resolved by SDS-PAGE (Figure 1B, C). Excised protein bands were subjected to tandem mass spectrometry (MS). We performed a total of seven IP-MS analyses under the same experimental conditions on mouse embryos (N = 3) and HEK293 cells (N = 4).

All co-IP'd proteins were identified by detection of multiple peptides. MS confirmed Tbx3/TBX3 in the IPs (Figure 1D) with a molecular weight of ∼85 kDa. Only proteins present in at least two independent IP/MS datasets and not in negative control IPs were considered further: 75 interacting proteins met these criteria (Table S1). Representative MS traces are shown of hnRNPU, Raver1, and hnRNPK (Figure 1 E–G). Of the 73 interactors present in both species, 50 were detected by MS in both mouse and human co-IPs (Table S1).

### TBX3 interacts with RNA binding proteins and splicing factors *in vivo*

The known associations of TBX3 with HDACs and transcriptional co-repressors led us anticipate enrichment of such proteins in our MS screen. To our surprise, ontological classification of annotated functions, biological processes and functional domains demonstrated marked enrichment of RNA binding proteins (RNA BPs) and splicing factors among Tbx3/TBX3 interactors (Figure 1H, I, Table 1, Table S1). Forty-four percent of interacting proteins bind RNA (33/75), and many participate in splicing (Table 1). mRNA processing factors comprise 24% of the interactome (18/75). 20% contain RNP-1 and/or RRM (RNA recognition motif) domains (15/75, Figure 1H). Members of the ribonucleoprotein complex and RNA BPs are more than 30 fold enriched (Figure 1I) compared to the entire proteome. Over-representation of RNA BPs is not due to bias from use of antibody against the TBX3 C-terminus because many interactors were also co-IP'd with a commercial antibody against an internal epitope (Table S1, “SC”).

**Table 1:** TBX3 interacting proteins involved in mRNA splicing.

NAME	UNIPROT	ANNOTATION	SOURCE *
Heterogeneous nuclear ribonucleoprotein A1-like 2	Q32P51	Spliceosome mRNA splicing	GO, KEGG, UP
Heterogeneous nuclear ribonucleoprotein A1-like 3	P09651	Spliceosome mRNA splicing	GO, KEGG, UP
Heterogeneous nuclear ribonucleoprotein A2/B1	P22626	Spliceosome mRNA splicing	GO, UP
Heterogeneous nuclear ribonucleoprotein A3	P51991	Spliceosome mRNA splicing	GO, KEGG, UP
Heterogeneous nuclear ribonucleoprotein C (C1/C2)	P07910	Spliceosome mRNA splicing	GO, KEGG, UP
Heterogeneous nuclear ribonucleoprotein H1	P31943	Spliceosome mRNA splicing	GO, UP
Heterogeneous nuclear ribonucleoprotein K	P61978	Spliceosome mRNA splicing	GO, KEGG, UP
Heterogeneous nuclear ribonucleoprotein M	P52272	Spliceosome mRNA splicing	GO, KEGG, UP
Heterogeneous nuclear ribonucleoprotein U	Q00839	Spliceosome mRNA splicing	GO, KEGG, UP
Poly(A) binding protein, cytoplasmic 1	P11940	Spliceosome mRNA splicing	GO, UP
Heat shock 70 kDa protein 1-like	P34931	Spliceosome	KEGG
Heat shock 70 kDa protein 1A; heat shock 70 kDa protein 1B	P08107	Spliceosome	KEGG
Heat shock 70 kDa protein 2	P54652	Spliceosome	KEGG
Heat shock 70 kDa protein 8	P11142	Spliceosome Alt splicing	KEGG, UP
Poly(rC) binding protein 1	Q15365	Spliceosome	KEGG
ATP-dependent RNA helicase DDX5	P17844	Spliceosome mRNA splicing	GO, KEGG, UP
Probable ATP-dependent RNA helicase DDX17	Q92841	RNA binding Alt splicing	GO, UP
ATP-dependent RNA helicase DDX3X	O00571	RNA binding	GO, UP
Ribonucleoprotein PTB-binding 1	Q8IY67	RNA binding Alt splicing	GO, UP

* GO: Gene Ontology; KEGG: Kyoto Encyclopedia of Genes and Genomes; UP: Uniprot.

To further validate the MS results, we assayed interactions between endogenous Tbx3/TBX3 and a subset of candidates detected by MS known to be involved in pre-mRNA splicing and RNA metabolism. In mouse embryos, Tbx3 co-IP'd with endogenous Pabp1, Ddx17, hnrnp u, hnrnp k, and Fmr1 (Figure 1 O–R). Endogenous hnRNP C, DDX17, DDX3, hnRNP U, PABP1 and FMRP co-IP'd with endogenous TBX3 in HEK293 lysates (Figure 1 S–V, and Figure S1). Thus the association *in vivo* between Tbx3/TBX3 and splicing factors is conserved in mouse and human. We further tested 4 of these TBX3 binding partners for association with overexpressed, His-Myc- dually tagged TBX3 in Ni-NTA pull-down assay followed by anti-myc co-IP (Figure S1, A–E). All 4 interactions tested withstood both the pull down and the subsequent co-IP.

We then tested whether interactions between TBX3 and a subset of RNA BPs are RNA dependent in HEK293 cells. RNAse treatment did not disrupt the interactions between TBX3 and hnRNPC, DDX3 or PABP1, but slightly decreased the amount of FMRP that co-IP'd with TBX3 (Figure S1, F–G), indicating that RNA is not absolutely required for the interactions tested.

DDX3 was the most robust interactor with dually tagged TBX3 in the Ni-NTA pulldown/co-IP assay, therefore we tested whether its interaction with TBX3 was direct using purified GST-DDX3 and MBP-TBX3 fusion proteins (Figure S1K) in a pull-down assay followed by immunoblotting for TBX3. MBP-TBX3 bound to GST-DDX3, but not GST alone (Figure S1L, lane 3 vs 6).

### TBX3 DNA-binding and C-terminal domains mediate interactions with different RNA BPs

**Figure 2:**
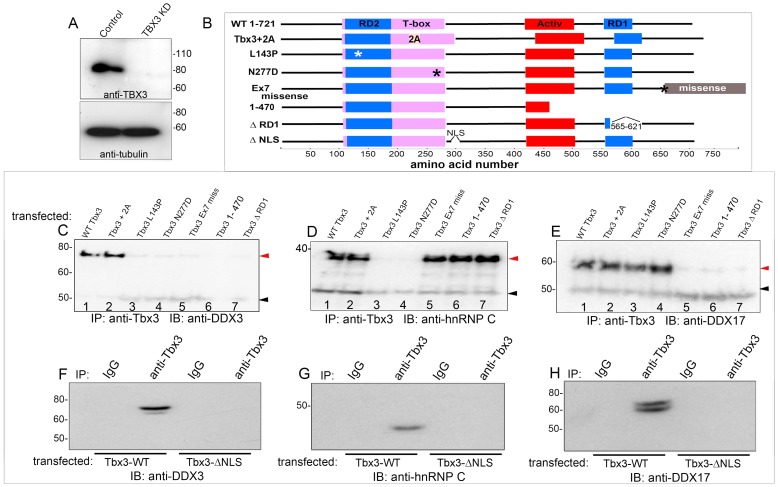
Different Tbx3 protein domains are required for protein-protein interactions. Stable retroviral mediated knockdown of endogenous TBX3 in HEK293 cells followed by transfection of wild type and Tbx3 mutant proteins as diagrammed. Lysate IPs were assayed by IB with antibodies noted at bottom of panels. A) Immunoblot of control versus *TBX3* shRNA (KD) lysates. B) Schematic of Tbx3 mutant proteins. Confirmation of expression and successful IP of overexpressed Tbx3 proteins is presented in Figure S2. C–E) Interactions of mutant Tbx3 proteins with DDX3, hnRNP C, and DDX17 tested by IP and western blotting. F–H) Tbx3 NLS is required for Tbx3 interactions with DDX3, DDX17 and hnRNP C. red arrowheads: interacting proteins; black arrowheads: IgG.

To determine which protein domains of TBX3 mediate its interactions, we used viral shRNA transduction to knockdown endogenous TBX3 in HEK293 cells (Figure 2A) and then transfected various Tbx3 expression constructs (Figure 2B). This allowed us to test requirements for different Tbx3 functional domains without simultaneously detecting interactions with endogenous TBX3. Tbx3+2a contains an additional 20 amino acids (aas) in the DBD. Point mutations N277D and L143P identified in humans with UMS abolish DNA-binding activity [22]. Tbx3ΔRD1 lacks the C-terminal repressor domain [15]. The frameshift encoded by the *Tbx3* “ex7 miss” missense mutation substitutes the final 65 aas and generates a 765 aa protein (please see methods). C-terminal deletions are as shown. All variants are translated into protein post transfection into HEK293 cells (Figure S2A), and are efficiently IP'd (Figure S2B, C).

Immunoblot analysis of Tbx3 IP'd proteins with anti-DDX3 demonstrates that DDX3 associates with Tbx3 +/− exon 2a. DBD mutations (Figure 2C, lanes 3, 4) abrogate this interaction, as do C-terminal and RD1 deletions (Figure 2C, lanes 6, 7). Tbx3 lacking a nuclear localization signal (Tbx3ΔNLS) does not interact with DDX3 (Figure 2F). The DBD and NLS are required for Tbx3/hnRNP C interaction (Figure 2D: lanes 3, 4 and 2G), but C-terminal and RD1 deletions had no effect (Figure 2D, lanes 5–7). The C-terminus, RD1 and NLS are required for Tbx3/DDX17 interaction (Figures 2E: lanes 5–7 and 2H), while DBD mutation had little effect (Figure 2E, lanes 3, 4). We conclude that nuclear localization of TBX3 is required for the interactors tested thus far, whereas the C-terminus, RD1 and DBD are independent and variably required to mediate interactions between Tbx3 and different RNA BPs. Combined with the RNAse data, our results indicate that no one “rule” constrains the interaction of TBX3 with its binding partners.

### Tbx3 regulates alternative splicing *in vitro* and *in vivo*

**Figure 3:**
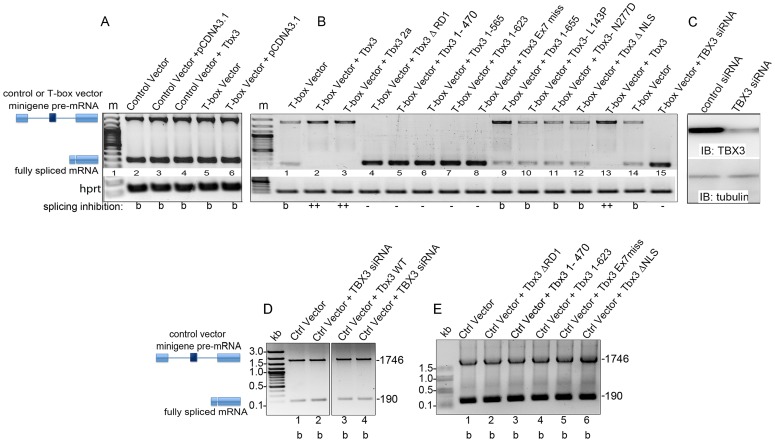
Tbx3 regulates alternative splicing *in vitro*. A) Either pRHCglo (Control vector) or RHCGlo+T-box binding element (T-box vector) were co-transfected into HEK293 cells with Tbx3 expression vectors indicated above each lane. Splicing products were assayed by RT-PCR; positions of unspliced pre-mRNA and spliced products are indicated at left. Hprt control PCR products are shown below. B) Ability of Tbx3 mutant proteins to regulate T-box vector splicing. Symbols at bottom of panel summarize effect on splicing inhibition. b, baseline ratio of pre-mRNA to completely spliced mRNA; +, splicing inhibited over baseline; −, no inhibition. C) Immunoblot of control or TBX3 siRNA lysates probed for TBX3 and tubulin. D) Splicing products from the control minigene vector in the presence of Tbx3 or Tbx3 knockdown, assayed by RT-PCR. Schematics and positions of unspliced pre-mRNA (1746) and fully spliced (190) products are indicated at left. Baseline (b) ratio of unspliced to fully spliced mRNA from the control minigene vector is unchanged by overexpression of Tbx3 or knockdown of endogenous TBX3 in HEK293 cells. E) Splicing products from the control minigene vector in the presence of Tbx3 mutant proteins assayed by RT-PCR. In the absence of a TBE in the minigene, Tbx3 mutant proteins have no effect on the baseline ratio of unspliced to fully spliced mRNA.

Given the overrepresentation of splicing factors among Tbx3 interactors, we tested whether Tbx3 regulates splicing using the pRHCglo minigene developed by Singh and Cooper: in CosM6 cells, they showed that the second exon regulates splicing of the pre-mRNA transcribed from this minigene [23]. We first tested whether the pRHCglo pre-mRNA was spliced in HEK293 cells (“Control vector”, Figure 3A, lane 2) by transfecting the plasmid into the cells and assaying for transcription/splicing products produced from the minigene using reverse transcription-PCR (RT-PCR). We detected the complements of two mRNAs and sequencing revealed that the 1700 bp cDNA is the RT product of the unspliced pre-mRNA, while the 190 bp cDNA is produced from the completely spliced transcript, as shown schematically in Figure 3A.

Splicing of transcripts from the control vector pRHCglo was not affected by overexpression of Tbx3 (Figure 3A, lane 4) or by knockdown of endogenous TBX3 (Figure 3D, lane 4). We replaced the second exon of pRHCglo (32 bp, shown by Singh and Cooper to regulate splicing) with a T-box DNA binding element (TBE, 31 bp) to generate the “T-box vector” splicing reporter and again detected two splicing products by RT-PCR (Figure 3A: lanes 5–6). We then examined effects of Tbx3 mutations on splicing of the T-box vector pre-mRNA. Relative to baseline (“b”, Figure 3B, lane 1), Tbx3 and Tbx3+2a inhibit splicing (Figure 3B, lanes 2, 3), and this requires the TBE (Figure 3A, lane 4 vs. 3B, lane 2). Consistent with the domain requirements for interactions, ΔRD1, exon7 missense mutation, or C-terminal deletions prevented Tbx3 splicing inhibition (Figure 3B: lanes 4–8). Indeed, all detected reporter mRNA is fully spliced in the presence of C-terminal mutants that lack the repressor domain, revealing a dominant effect over the factor(s) that inhibit splicing at baseline. This suggests that binding of N-terminal portions of mutant proteins to the TBE in the T-box vector DNA (or nascent transcript) disrupts the function of factors that normally inhibit splicing. This effect is dependent on the TBE because these mutant proteins had no effect on splicing of the Control vector (Figure 3E). Baseline splicing was observed with the L143P, N277D and Tbx3ΔNLS mutants (Figure 3B, lanes, 10–12). Knockdown of endogenous TBX3 regulates the ratio of unspliced/spliced T-box vector mRNA (Figure 3C) such that only fully spliced mRNA is present (Figure 3B, lane 15). In combination with the observation that knockdown of endogenous TBX3 has no influence on splicing of the Control vector pre-mRNA (no TBE, Figure 3D), we conclude that endogenous TBX3 inhibits splicing of T-box vector pre-mRNA via the TBE.

The *in vitro* splicing results led us to test whether Tbx3 regulates splicing *in vivo*. We conditionally ablated *Tbx3* function in e10.5 mouse embryo forelimb mesenchyme using *Prx1Cre* [24] and assayed for differential splicing using mRNA sequencing (RNA-Seq). We microdissected control and *Tbx3;Prx1Cre* mutant forelimb buds into anterior and posterior segments, made cDNA libraries from the mRNA obtained (2 from each geno/tissue type) and performed deep sequencing Messenger RNAs with annotated alternate splice forms (UCSC knownAlt [25]) were evaluated for differential exon usage events between WT and Tbx3 ablated samples based on RPKM values. The following criteria were used to identify statistically significant alternative splicing events: Fisher's exact test p< = 0.05, Bayesian error rate < = 0.1, fold change > = 1.5, reads supporting event > = 15 (please see Supplemental Information for complete informatics methodology).

**Figure 4:**
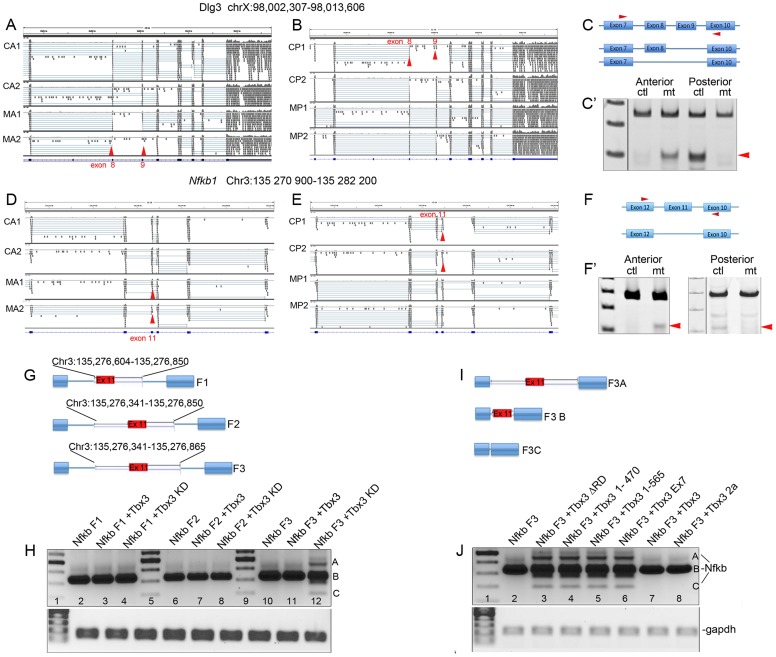
Tbx3 regulates alternative splicing *in vivo*. A, B, D, E) Screen shots from the Integrated Genome Viewer (IGV) comparing RNA-Seq reads obtained from wild type/control anterior (CA1, CA2) or posterior (or CP1, CP2) limb bud mRNA libraries with those from *Tbx3;Prx1Cre* mutants after conditional ablation of *Tbx3* in anterior or posterior limb bud mesenchyme (MA1, MA2 or MP1, MP2, respectively). Red arrowheads indicate positions of exons that are differentially spliced in a Tbx3-dependent manner. A) Loss of Tbx3 causes exclusion of *Dlg3* exons 8 and 9 in the mutant anterior compartment (MA1, MA2 red arrowheads). B) Loss of Tbx3 causes inclusion of *Dlg3* exons 8 and 9 in the mutant posterior compartment (MP1, MP2 red arrowheads). C) Schematic of splice variants and location of PCR primers used to detect different *Dlg3* isoforms by RT-PCR assay of control versus *Tbx3* mutant mRNAs (C′, Anterior and Posterior compartments, ctl, control; mt, mutant). D) Loss of Tbx3 causes exclusion of *Nfkb1* exon 11 in the mutant anterior compartment (MA1, MA2 red arrowheads). E) Loss of Tbx3 causes inclusion of *Nfkb1* exon 11 in the mutant posterior compartment (MP1, MP2 red arrowheads). F) Schematic of splice variants and location of PCR primers used to detect different *Nfkb1* isoforms by RT-PCR assay of control versus *Tbx3* mutant mRNAs (F′, Anterior and Posterior compartments, ctl, control; mt, mutant). G) Schematic of *Nfkb1* exon 11 minigenes in which exon 2 of pRHCglo was replaced by different *Nfkb1* genomic fragments (F1, F2, F3). H) Splicing products assayed by RT-PCR from the *Nfkb1* minigenes containing fragments F1, F2 or F3 in the presence of Tbx3 or after knockdown (KD). Only F3 confers differential splicing (lane 12). I) Schematic of splice variants obtained *in vivo* (sequence confirmed) in response to TBX3 knockdown. J) Splicing products assayed by RT-PCR from the *Nfkb1* F3 minigene in the presence of Tbx3 different Tbx3 mutant proteins. C-terminal mutants (lanes 3–6) result in alternate splicing similar to Tbx3 knockdown.

We randomly selected 11 of the statistically significant exon alternative splicing (AS) events identified *in silico* in the anterior mesenchyme (Tables S2 and S3) for validation by RT-PCR (Figure 4A–F′, Figures S3 and S4A). All statistically significant events tested validated, whereas most events tested that were below the false discovery rate of 5% did not (Tables S2, S3, Figure S4A, B). RNA-Seq reads visualized with the Integrated Genome Viewer (IGV) show that remarkably, Tbx3 has opposite effects on *Dlg3* splicing in the anterior and posterior limb: in the anterior limb bud Tbx3 promotes inclusion of exons 8 and 9 (Figure 4A, C′: loss of Tbx3 results in increased levels of the short isoform) whereas in the posterior, Tbx3 promotes exon skipping (Figure 4B–C′). Similar findings occurred with *Nfkb1* exon 11 (Figure 4D–F′). Additional examples of Tbx3 variably promoting inclusion or skipping are shown in Figure S3. These experiments reveal that Tbx3 influences AS in a transcript- and tissue-specific manner *in vivo*.

Since altered splicing after ablation of Tbx3 could be indirect or secondary to transcriptional effects, we tested whether Tbx3 directly regulates splicing of the *in vivo* target *Nfkb1*. We replaced exon 2 of pRHCglo with *Nfkb1* exon 11 (92 bp) flanked by 3 different fragments from the adjacent introns (Figure 4G). Primary sequences of these fragments contained the number of TBE cores (GGTG or CACC, please see Supplemental Methods in Text S1 for sequence of each fragment) predicted by chance. However, a motif located 4 bps from the splice donor site of exon 10 was in the larger context of a TBE consensus motif (5′ A/TGGTGTG) [26]. Remarkably, the only difference between Fragment 2 and Fragment 3 was the addition of 15 bps containing this consensus motif, and only *Nfkb1* minigene Fragment 3 (Nfkb1 F3) was alternately spliced in response to TBX3 knockdown, which resulted in *Nfkb1* minigene exon 11 skipping and inclusion (Figure 4H, lane 12), as observed *in vivo* (Figure 4F′). Figure 4I schematically shows splice variants (sequence confirmed) resulting from TBX3 knockdown. This is consistent with the T-box vector minigene experiments which showed that TBX3 requires a TBE to regulate splicing (Figure 3A, B). Deletion/mutation of TBX3 C-terminal domains previously demonstrated to be required for TBX3 interactions have the same effect on *Nfkb1* minigene splicing as *TBX3* knockdown (Figure 4J, lanes 3–6).

### TBX3 associates directly with alternately spliced and TBE -containing RNAs

**Figure 5:**
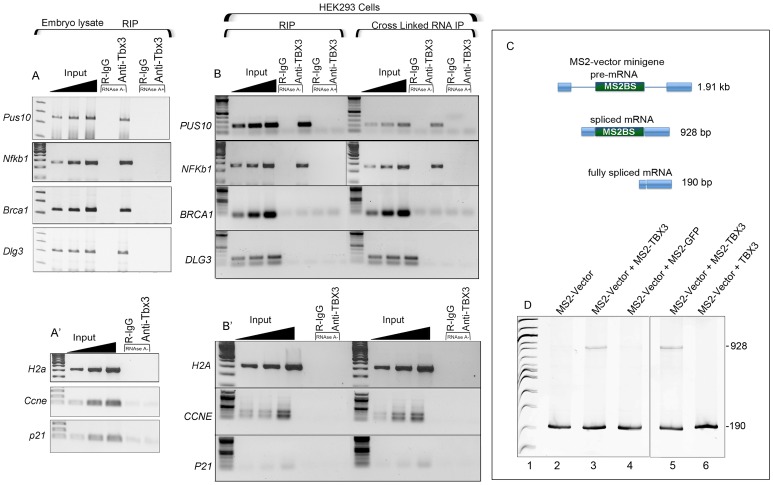
Endogenous Tbx3/TBX3 associates with specific mRNAs in mouse embryonic tissues and HEK293 cells. A, A′) RNA-IP with anti-Tbx3 on embryo lysates in the presence or absence of RNAseA followed by RT-PCR for transcript detection. Tbx3 binds *Pus10*, *Nfkb1*, *Brca1* and *Dlg3* mRNAs. A′) H2a, *Ccn*e and *p21* are negative controls for the positive interactions seen in panel A, as Tbx3 does not bind/RIP these mRNAs. B, B′) RIP and cross-linked RNA-IP (CLIP) of HEK293 lysates followed by RT-PCR. Endogenous TBX3 binds the *PUS10* and *NFKb1* mRNAs but not *BRCA1*, *DLG3* (B) or negative controls (B′). C) Schematic of splicing products from a novel splicing reporter containing multimeric MS2 binding sites in exon 2 of the pRHCglo minigene (MS2-vector); the unspliced pre-mRNA (1.91 kb), partially (928 bp), and fully spliced (190 bp) products are shown. D) RT-PCR analysis to detect splicing products from the MS2 reporter RNA in HEK293 cells. In the presence of MS2-TBX3, a partially spliced mRNA is present consisting of exons 1–3, but no introns (927 bp, lanes 3, 5). MS2-GFP (lane 4) and wild type TBX3 (lane 6) have no effect.

Splicing complexes are recruited to the exon-intron and/or exon-exon junctions on pre-mRNAs. TBX3's effects on splicing and the RNA–independence of interactions with RNA BPs/splicing factors (Figure S1A) suggest physical association of TBX3 and mRNAs *in vivo*. To test this, we performed RNA immunoprecipitation (RIP) and RT-PCR. Anti-Tbx3 RIP on mouse embryo mRNA/protein showed that *Pus10*, *Nfkb1*, *Brca1*, and *Dlg3* transcripts were associated with Tbx3 (Figure 5A, lanes 5), while the negative controls mRNAs *H2a*, *Ccne* and *p21* (Figure 5A′) were not. Endogenous TBX3 RIP'd *PUS10* and *NFKB1* mRNAs in HEK293 cells as well (Figure 5B), but not *BRCA1* or *DLG3*, which further supports the conclusion that Tbx3/TBX3 regulation of splicing is transcript and context dependent.

These surprising results led us to determine if TBX3 associates directly with RNA. TBX3 could influence splicing of the T-box vector minigene (Figure 3) or Nkb1 (Figure 4) by binding to the TBE in the reporter or genomic DNA in a cotranscriptional processing mechanism, or by binding to the pre-mRNAs. We generated an MS2-TBX3 fusion protein in which the MS2 RNA binding protein domain was fused in frame at the N-terminus of TBX3 (MS2-TBX3). We replaced exon 2 of pRHCglo with multimeric MS2 binding sites (“MS2-Vector). Note that the MS2 binding site only forms in RNA [27]. The MS2-vector is fully spliced in HEK293 cells to a form that contains only exons 1 and 3 (190 bp, Figure 5C, D lane 1). In the presence of MS2-TBX3, a partially spliced mRNA is present consisting of exons 1–3, but no introns (928 bp, Figure 5C, D lanes 3,5). This splice variant was not present when either MS2-GFP or wild type TBX3 were employed (Figure 5D, lanes 4 and 6 respectively). These results indicate that MS2-TBX3 regulates splicing of the reporter by binding the MS2 binding site in the mRNA. Furthermore, complete splicing inhibition seen with the T-box vector (Figure 3B) was not observed for the MS2-vector transcript, consistent with our other results indicating that the effects of TBX3 on splicing are transcript dependent.

**Figure 6:**
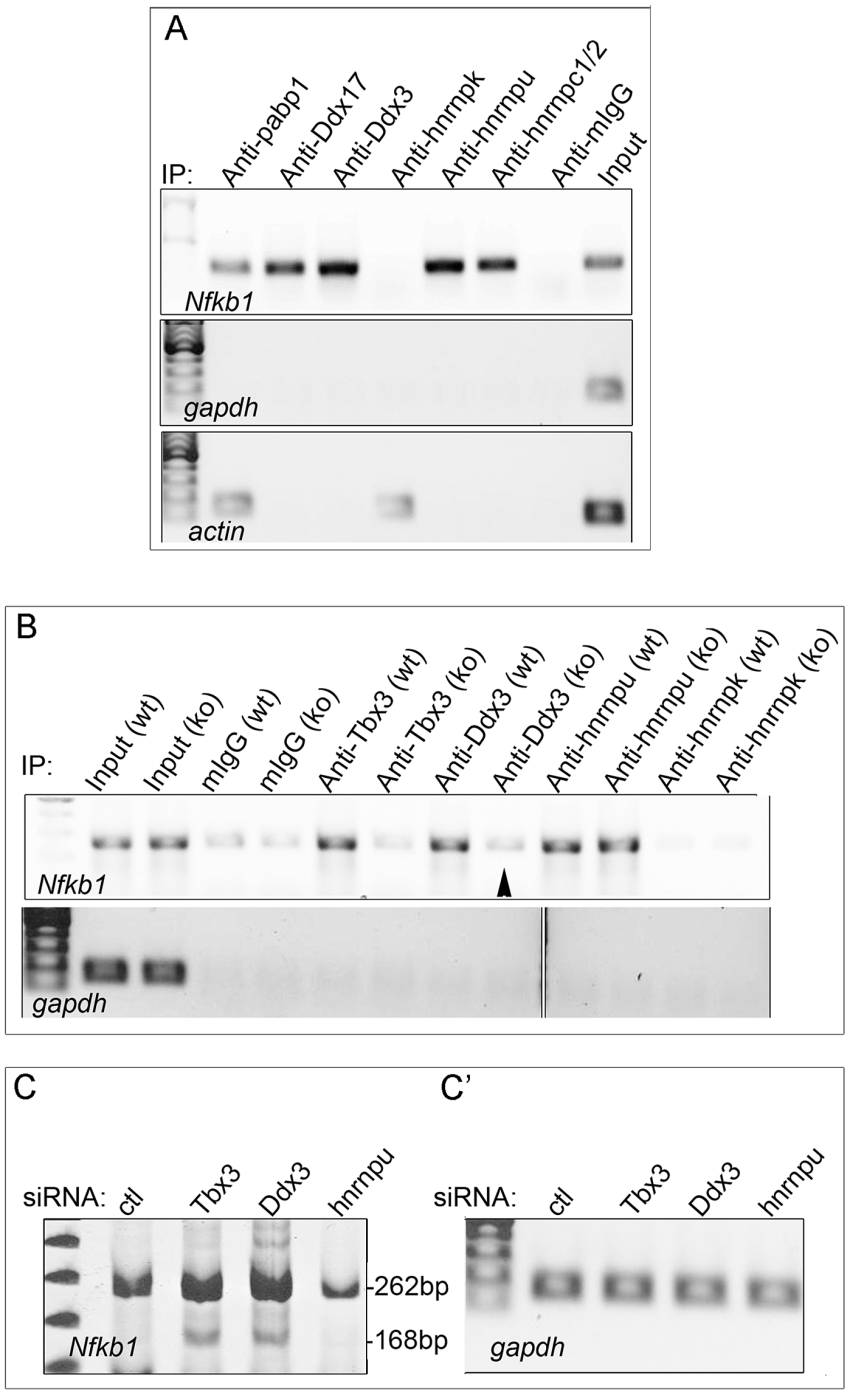
Tbx3 binding partners associate with the *Nfkb1* mRNA that is alternatively spliced in response to Tbx3, and a subset require Tbx3 to bind this mRNA. A) RNA-IP with anti-Tbx3 on embryo lysates using antibodies against Tbx3 interacting proteins to test binding to the *Nfkb1* transcript which is differentially spliced in response to Tbx3 in mouse limb. Actin and Gapdh are negative controls. Note that not all Tbx3 interactors bind this mRNA. B) RIP/RT-PCR testing Tbx3 interactors for binding of the *Nfkb1* mRNA in *Tbx3* wild type (wt) and null (ko) murine embryonic fibroblasts (MEFs). Ddx3 requires Tbx3 to bind the *Nfkb1* mRNA (arrowhead) but hnrnpu does not. C) RT-PCR assay of splice variants in control, *Tbx3* and *Ddx3* siRNA knockdown MEFs. Knockdown of *Ddx3* results in the same alternative splicing of *Nfkb1* exon 11 as *Tbx3* knockdown, whereas knockdown of hnrnpu decreases the total amount of *Nfkb1* transcript but has no effect on exon 11 splicing. C′) *Gapdh* control RT-PCR.

To probe the mechanism whereby Tbx3 interactions with RNA BPs and RNA influence splicing, we tested whether interacting proteins also bind the *Nfkb1* mRNA in mouse embryonic tissue. Of the 6 interactors tested (Figure 6A), only hnrnpk did not bind this mRNA. As with Tbx3, binding of these interactors to *Nfkb1* mRNA is specific as they do not bind *gapdh* or *actin* mRNAs (Figure 6A). We next tested whether Tbx3 was required for *Nfkb1* binding by Ddx3 and hnrnpu (the most robust *Nfkb1* binders) by assaying mRNA/protein complexes in tissues from wild type and *Tbx3* null embryos. Loss of Tbx3 abolished the interaction between Ddx3 and Nfkb1 mRNA, but not hnrnpu (Figure 6B). Additionally, knockdown of Ddx3, but not hnrnpu, had the same effect on *Nfkb1* splicing as knockdown of Tbx3 (Figure 6C). These results indicate that Tbx3 functions to recruit and/or dock some interactors to mRNAs that are alternately spliced in a Tbx3-responsive manner.

**Figure 7:**
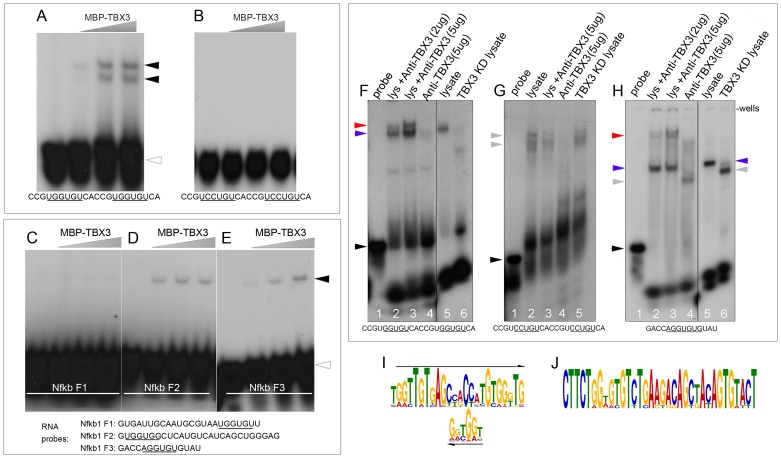
TBX3 binds TBE-containing RNAs directly. A–E) EMSA assays of purified, MBP-conjugated Tbx3 with radiolabeled RNA probes whose sequences are listed at bottom of panels. C–E) RNA probes derived from *Nfkb1* intronic fragments (F1–F3). Black arrowheads: probe/protein complex; clear arrowheads: unbound probe. F–H) Shift and supershift RNA EMSA assays of endogenous TBX3; radiolabeled probe sequences are listed below panels. Probes were incubated +/− HEK293 lysate, +/− anti-TBX3 antibody, +/− TBX3 knockdown. F) RNA probe containing GGUGU motifs. Probe/protein complex (lane 5) supershifts with anti-TBX3 antibody (lanes 2, 3) and disappears with TBX3 knockdown (lane 6). TBX3+RNA complexes are indicated by blue arrowheads, anti-Tbx3 antibody supershifted TBX3+RNA complexes are indicated by red arrowheads and unbound probe by black arrowheads. Gray arrowhead indicates probe-protein complexes detected in the absence of TBX3 (lane 6). G) Mutation of the GGUGU to CCUGU results in formation of probe/protein complexes (lane 2) does not supershift with anti-TBX3 antibody (lane 3) and unaffected by TBX3 knockdown (lane 5). H) RNA probe containing AGGUGUG consensus TBE motif from *Nfkb1* Fragment 3. Probe/protein complex (lanes 2, 3, 5, blue arrowhead) supershifts with anti-TBX3 antibody (lanes 2, 3, red arrowheads) and decreases with TBX3 knockdown (lane 6). Gray arrowhead indicates probe-protein complexes detected in the absence of TBX3 (lane 6). Probe incubated with no lysate (F–H, lanes 1) or anti-TBX3 antibody alone (F–H, lanes 4) are additional negative controls. IgG negative control EMSAs are shown in Figure S5 G, H. I, J) Significantly over-represented sequence motifs identified by MEME on genomic regions flanking statistically significant AS events contain the 5′GGTG T-box core binding element.

To further examine the interaction of TBX3 directly with RNA, we performed EMSAs with RNA probes and purified TBX3 protein. Of 14 variably complex RNA probes tested, TBX3 bound poly-GGU, -AG, -GU RNAs (Figure S5A–C), see Supplemental Information in the file Text S1 for all probes tested). It also bound strongly to a probe containing two RNA-TBEs 5′UGGUGU (Figure 7A), but not to a probe with mutated TBEs (Figure 7B) Further, we examined TBX3 binding to RNAs from *Nfkb1* F1-3 (previously assayed in the minigene, Figure 4I, J). The F1-derived probe was not bound despite the presence of a 5′UGGUGU motif (Figure 7C). Although F2 was not sufficient to drive minigene splicing, TBX3 bound this fragment, as well as A consensus TBE present in F3 (Figure 7D,E). Mutation of the TBEs in F2 and F3 disrupted binding by TBX3 (Figure S5, panel F). In combination with the *Nfkb1* minigene splicing findings, these results reveal that TBE context is critical for both RNA binding and splicing regulation.

We next tested whether endogenous TBX3 in HEK293 cells binds RNA. Lysates were incubated with radiolabeled RNA probe +/− anti-TBX3 antibody and +/− TBX3 knockdown. Probe containing 2 RNA-TBEs (5′ UGGUGU) was shifted by proteins in wildtype lysate (Figure 7F, lane 5, blue arrowhead) and supershifted by anti-TBX3 antibody (Figure 7F, lanes 2, 3, red arrowhead). Negative control antibody does not supershift these complexes (Figure S5G, lane 6). In TBX3 knockdown lysate, a different complex was detected (Figure 7F, lane 6, gray arrowhead). Mutation of the RNA to 5′UCCUGU resulted in shifted probe/protein complexes that were insensitive to anti-TBX3 antibody or loss of TBX3 (Figure 7G lanes 2, 3, 5, gray arrowheads). Similar experiments with the RNA motif from *Nkb1* F3 revealed a complex (Figure 7H lane 5; blue arrowhead) that was supershifted by anti-TBX3 antibody (Figure 7H, lanes 2, 3; red arrowhead) and lost with TBX3 knockdown (Figure 7H, lane 6). The supershift did not occur with negative control antibody (Figure S5H, lane 6). Collectively, these data indicate that TBX3 binds RNA containing RNA-TBEs and in the absence of TBX3, other protein(s) bind these RNAs.

We defined putative TBEs conservatively based on the literature [26], [28]: 5′ T/A GGTG T/A/G. Among the 11 statistically significant alternatively spliced genes in the anterior limb compartment that we validated, this TBE is present in the introns flanking the alternatively spliced exons of *Nfkb1*, *Dlg3*, *Ttc3*, *Pus10*, *Brca1*, *Fanca*, *Cacnb3*, *Arnt2*, *Wdr70* (9/11, Table S4). Not all of these sites were conserved, although for some sites, there was conservation to opossum and deeper.

MEME motif analysis of 1 kb of sequence flanking statistically significant alternatively spliced exons from the anterior RNA-Seq dataset (Table S3) revealed that SINE motifs containing TBEs were highly overrepresented (Figure 7I, J, Table S4). The MEME motifs shown were present in 20 and 10 of 51 statistically significant alternative splicing events detected with Expect values of 1.1 e^−96^ and 3.3 e^−83^, respectively. MEME mapping did not reveal a pattern or consistent location or orientation of these overrepresented motifs relative to the alternative splicing event (Table S4). A Tbx3 motif with a less stringent core (5′ G/T G/C TG N) was identified by MEME on Tbx3 ChIP-Seq data from adult mouse heart [28]. This sequence (as well as 5′ T/A GGTG T/A/G) is present within the larger MEME motifs we identified (Figure 7 I, J). Among the 11 AS genes we validated, these MEME motifs are present in *Dlg3*, *Dtnb1*, *Pus10*, *Brca1*, *Fanca1*. Table S4 shows location and conservation of putative TBEs and MEME motifs for all 11 validated alternative splicing events in the anterior compartment. In total, the data indicate that splicing outcomes are affected by binding motifs present near alternatively spliced exons, but the splicing outcomes are context and transcript dependent.

## Discussion

We used a rigorous series of biologic replicates, controls and statistical filters to identify TBX3 interacting proteins in HEK293 cells (75 interactors) and E10.5 mouse embryos (59 interactors) by mass spectrometry. This unbiased proteomic screen revealed that TBX3/Tbx3 interacts with RNA BPs and splicing factors. Our discovery that TBX3 regulates alternative splicing *in vivo* suggests new mechanisms of TBX3–mediated regulation of target genes. Importantly, TBX3 proteins that model different human UMS mutations have different splicing activities: C-terminal mutants dominantly interfere with splicing inhibition mediated by endogenous TBX3, while DNA binding and NLS mutants neither inhibit splicing nor have dominant effects.

Tbx3 physically associates with mRNAs that undergo AS in response to *Tbx3* loss of function *in vivo*, and directly binds RNA containing the core motif of T-box DNA-binding elements (RNA-TBEs). Since AS is a critical mechanism of post- and co- transcriptional gene regulation and proteome diversity and disruption of AS occurs in many cancers [29], [30], [31], the finding that TBX3 regulates this process provides new insights into how altered dosage and molecular functions of TBX3 contribute to human developmental disorders and cancer. For example, the mechanism(s) of repression of *CDKN2A*, which is alternatively spliced to produce the p16^INK4^ and p14^ARF^ tumor suppressors, will need to be reexamined for potential post-transcriptional effects of TBX3.

Interactions between TBX3 and specific RNA-binding proteins require different TBX3 functional domains indicating that crosstalk with different molecular partners mediates distinct TBX3 functions. We tested the effect of NLS deletion on 3 Tbx3 interactors: DDX3, hnRNPC and DDX17, and the data suggest that these specific interactions take place within the nucleus. Unlike wild type Tbx3, the Tbx3 NLS mutant does not inhibit splicing, nor does it have the dominant effect on splicing seen with most C-terminal mutants (which still get to the nucleus, Moon unpublished and [15]). These findings are consistent with the overwhelming evidence that for the majority of mRNAs, splicing is a co-transcriptional event [32]. Many of the Tbx3 interacting proteins we identified (DDxs, hnRNPs, Fmr1 and others) are known to be present in both the nucleus and the cytoplasm. Wild type Tbx3 is also present in the cytoplasm [21], [33], thus it is likely that Tbx3 interacts with some partners outside the nucleus. DDX3, DD17, hnRNPc1, hnRNPU have all been previously reported to play role in splicing by directly binding to RNA or via the splicing complex; a major shift has occurred in the field due to the discovery that the majority of splicing is cotranscriptional and nuclear, thus some functions of these proteins previously attributed to the cytoplasm are nuclear. Our study reveals novel properties of these proteins in terms of their interaction with TBX3 and that in some cases, the ability of the RNA BP to influence splicing is TBX3-dependent. For example, although DDX3 has previously been shown to regulate splicing, we have discovered a novel DDX3 splicing target pre-mRNA (*Nfkb1*) and shown that DDX3 requires TBX3 to bind this target. Future studies of the functional consequences of interactions with TBX3 may reveal completely novel functions for some of its partners.

Although we have discussed the TBX3 DBD as such, TBX3 complexes with AS RNAs *in vivo* and directly binds RNAs containing TBEs. The *Nfkb1* minigene and EMSA results reveal that the context of RNA-TBEs is critical for TBX3 to affect splicing. TBE motifs are over-represented in sequences flanking alternative exon events resulting from loss of Tbx3 *in vivo* contain, including SINE motifs. This is noteworthy because evidence for crucial regulatory functions of SINEs is accumulating [34]. MEME mapping of sequences flanking statistically significant AS events did not reveal a pattern or consistent location of putative TBEs or other overrepresented motifs relative to the splicing event. The results with *Dlg3* and *Nfkb1 in vivo* and the *Nfkb1* minigene are very informative in this regard: these transcripts are alternatively spliced, and putative TBEs or MEME motifs are near the alternatively spliced exons. However, loss of Tbx3 has the opposite effect on splicing of these transcripts in the anterior and posterior compartments of the limb (Figure 4 A–F′): Tbx3 promotes exon inclusion of Dlg3 exons 8/9 and of *Nfkb1* exon 11 in the anterior, but exclusion of these exons in the posterior. Furthermore, inclusion of putative TBEs from different regions flanking *Nfkb1* exon 11 have different effects on splicing of the *Nfkb1* minigene (Figure 4G, H). Additional evidence of context dependence is the finding that Tbx3 and TBX3 both bind *Nfkb1* and *Pus10* mRNAs *in vivo* (in mouse embryo and HEK293 cells, respectively) but only Tbx3 binds *Dlg3* and *Brca* mRNAs. We conclude that alternative splicing outcomes are affected by binding motifs present near affected exons, but the outcomes are context and transcript dependent; future studies will test our postulate that this is due to interaction with different cofactors.

Splicing and transcription are most often coupled [32]: chromatin-associated factors aid in the recruitment of the splicing complex to nascent pre-mRNAs and regulate exon inclusion or exclusion [35]. HDAC inhibition disrupts AS of hundreds of pre-mRNAs [36]. Our screen identified TBX3 interactions with chromatin structural/modifiying factors H2A, H2B1B, TCP1 and PTB1. HDACs 1–5 also interact with TBX3 [5]. Our observation that, at least for the *Nfkb1* mRNA, TBX3 regulates AS by directly binding to an intronic RNA-TBE raises the issue of whether the effect of TBX3 on splicing are co-transcriptional and could also be mediated by TBX3 binding to TBEs in genomic DNA. We examined the published Tbx3 ChIP-Seq dataset [32] but did not find ChIP peaks within the 10 kb flanking the AS exons of any of the 11 validated genes. However, this ChIP-Seq was performed on adult mouse heart after forced pan-myocardial expression of Tbx3, whereas our RNA-Seq and splicing assays were on embryonic mouse limb. Additional studies to determine whether TBX3 influences splicing and gene expression by effects on chromatin and/or by binding DNA and RNA TBEs in alternatively spliced targets are underway.

We focused the present study on the splicing function of TBX3 with respect to novel binding partners however, it is unlikely that all interactions between TBX3 and RNA-binding partner proteins are related to splicing regulation: many of these interactors are known to have multiple roles and influence trafficking and other aspects of mRNA processing, and some have transcriptional effects and DNA binding properties. The numerous hnRNPs that interact with Tbx3 exemplify this point. This diverse family of multifunctional proteins has crucial roles in RNA processing in addition to AS. Dysregulation of hnRNP function and resulting disruption of AS contribute to carcinogenesis [37], [38]. Among the 12 hnRNP TBX3 interactors (Table 1), interactions with hnRNP U and hnRNP A1 could have widespread consequences: depletion of hnRNP U has profound effects on AS by regulating the maturation of the U2 snRNP and all snRNAs required for splicing [39], [40]. More AS events are altered by depletion of hnRNP A1 and U in human 293T cells than other hnRNPs [40]. Because hnRNPs exhibit context-dependent effects on splicing and RNA processing, they may modulate TBX3 target gene activity in a tissue-specific manner.

The association of TBX3 with several factors that regulate response to estrogen and other nuclear hormones (Table S1) [41], [42], [43], [44], [45] is interesting in the context of postulated roles for TBX3 in tumorigenesis and metastasis of hormone responsive breast, prostate and other cancers. DDX RNA helicases facilitate alternative promoter usage and splicing, and are transcriptional co-activators with ERα, p53 and Runx2 [46], [47], [48], [49]. Notably, TBX3 regulates both p53 activation and *Runx2* expression [17], [50]. Phb2 coregulates estrogen responsive genes by potentiating the effects of antiestrogens, inhibiting the effects of estrogens, and recruiting HDAC1 and 5 (both are Tbx3 interactors [5]) to nuclear hormone targets [42], [51]. XRCC6 interacts with Msx2 (a known TBX3 interactor) and Runx2 (a TBX3 target) [52] and mutations in *XRCC6* are associated with breast cancer risk and estrogen exposure [43]. We also detected Caperα (Coactivator of AP1 and Estrogen Receptor) in one co-IP by MS and by yeast 2-hybrid screen for Tbx3 interactors (Kumar et al., submitted). Caperα modulates steroid hormone receptor-mediated transcriptional regulation and AS [53]. Future studies will address the functional relevance of interactions between TBX3 and splicing factors in regulating estrogen response.

A comprehensive discussion of the other classes of TBX3 interactors identified in these analyses awaits validation of members of each class. However, the presence of multiple cytosolic and mitochondrial RNA and protein chaperones, nuclear import/export factors (including previously identified XPO1 [33]), ribosomal components, and nuclear membrane proteins suggest additional functions for TBX3 in both the nucleus and cytoplasm. The diverse interactome of TBX3 suggests that it may function as a docking molecule for the assembly of various RNA processing or regulating complexes and could functionally couple post-transcriptional processing and protein translation.

TBX5 was previously shown to interact with SC35 and regulate splicing [54]. The fact that SC35 was not detected as a TBX3 interactor by any of our screens suggests that the interactome of TBX3 and 5 are different, as would be predicted since there is minimal homology between these proteins outside the DNA binding domain. Thus, Tbx3 is among an increasing number of transcription and DNA binding factors that complex with splicing proteins and future studies to determine if other Tbx proteins bind RNA and/or influence splicing are warranted [55], [56], [57].

Numerous DNA binding transcription factors have now been demonstrated not only to bind RNA [58] but to bind a similar motif in RNA as in DNA, including some hnrnps [59], [60], [61], [62], [63].TBX3 may act as a docking molecule for assembly of RNA processing complexes, as has been postulated for Smad proteins: in addition to binding DNA of transcriptional targets, Smads bind to Smad binding elements in pre-miRNAs (R-SBEs) conferring BMP/TGFβ regulation to pre-miRNA processing [59]. Additional investigation is needed to determine how, and in what contexts, TBX3 exerts different post-transcriptional effects: it may function as a docking factor that directly binds pre-mRNAs, be recruited to pre-mRNAs via association with RNA BPs, or influence the activity of SINE or other regulatory elements.

The mutant Tbx3 proteins we tested for splicing activity *in vitro* represent the spectrum of mutations seen in humans with UMS, including the 1857delC frameshift [64]. Although we could not examine the splicing activity of these mutations *in vivo*, the minigene splicing assay (Figure 3) indicates that mutations that truncate the protein 5′ of the repressor domain dominantly interfere with the ability of endogenous TBX3 to inhibit splicing, while those that prevent DNA binding do not. The *Nfkb1* minigene is alternately spliced to both shorter and longer isoforms in the presence of Tbx3 mutants lacking the C-terminus (Figure 4). Based on data in OMIM (http://www.omim.org/) and a review of the literature, there are at least 10 human point mutations predicted to cause premature termination and loss of the C-terminal repressor domain. The human 1857 delC mutation is a missense mutation in the C-terminus; while not an exact replicate of this mutation, our exon7 missense mutation does produce a protein with an altered C-terminus and abnormal splicing activity. Our observation that knockdown of TBX3 alters splicing has significant implications that will require extensive further investigation since the presumed mechanism for *TBX3* loss of function mutations has been haploinsufficiency of transcriptional repressor activity. Furthermore, a subset of C-terminal human mutations encodes truncated proteins that undergo increased rates of protein decay; such mutations could thus disrupt splicing by multiple mechanisms. In combination with our observations that AS regulation by Tbx3 is context and mRNA dependent, the splicing functions of Tbx3 provide additional complexity to regulation of gene expression by Tbx3.

In conclusion, our study reveals that TBX3 is a splicing regulator and that mutations seen in humans with UMS disrupt this function. There is little genotype/phenotype correlation between and within UMS families, and phenotypes in mice are extremely dosage sensitive. We propose that the pleiotropic effects of *TBX3* mutations result from disrupting at least two context-specific molecular functions: transcriptional regulation and pre-mRNA splicing. Dissecting the requirements for different TBX3 molecular functions in specific developmental and disease contexts will improve our understanding of oncogenesis and UMS pathogenesis. The splicing function of TBX3 may be a new target for cancer treatment or in tissue regeneration efforts.

## Materials and Methods

### Cell culture and transfections

HEK293 cells were grown in DMEM with 10% FBS and pen/strep. Plasmids and transfection procedures are detailed in Supplemental Information in the file Text S1.

### Immunoprecipitation

Dignam lysates were prepared from HEK293 cells or e10.5 mouse embryos. Immune complexes were subjected to SDS-PAGE analysis followed by immunoblotting with specific antibodies. Detailed procedures are in SI.

### Enzymatic digestion of IP'd proteins and MS

IP'd proteins were separated by SDS-PAGE and in-gel digested prior to analysis by MS as previously described [65], [66]. GO annotation analysis was performed with DAVID Bioinformatics Resource as described in SI.

### RNA interference

HEK-293 cells were transfected with control or TBX3-specific siRNAs using lipofectamine 2000 (Invitrogen). RNA was extracted 48 hrs post-transfection and cDNA prepared with SuperScript III Reverse Transcriptase (Invitrogen).

### Retroviral transduction

High-titer retrovirus was produced by transfection of TBX3 shRNA retroviral construct along with gag/pol and VSVG encoding plasmids into HEK293 EBNA cells. Stably integrated colonies were selected and analyzed for TBX3 knockdown.

### RNA IP

Lysates were IP'd with anti-TBX3 and R-IgG. RNA was extracted with Tri reagent (Sigma), converted to cDNA. Primer details and CLIP are described in SI.

### RNA sequencing

Total RNA was isolated from pooled microdissected anterior and posterior segments of e10.5 wild type and *Tbx3* conditional mutant forelimb buds using the miRNeasy Kit (Qiagen). Libraries were prepared by the University of Utah Microarry Core and single end sequencing reads obtained on an Illumina HiSeq2000. Additional details and bioinformatic analyses are in SI.

## Supporting Information

Figure S1 Dual-tagged pull down and immunoprecipitation assays and RNA independence of TBX3 interactions. A–E) Immunoblots of Ni-NTA eluate and anti-Myc immunoprecipitation products from HEK293 cells expressing dually (His-Myc tagged)-TBX3 to examine association with endogenous TBX3 interactors. The lanes labeled input contain the lysate prior to application to the Ni-NTA column; those labeled Ni-NTA contain the Ni-NTA eluate, whereas those labeled anti-Myc contain dialyzed Ni-NTA eluate post IP with anti-Myc. The mouse IgG IP is a negative control. Immunoblots were then probed with the antibodies listed below the panels (IB). Endogenous hNRNPC1/C2, DDX3, DDX17 and PABP1 are all bound, eluted with, and coIP with His-Myc-TBX3. F–J) HEK293 lysates +/− RNAse A treatment were immunoprecipitated with the antibody listed at the top of the panels (IP) and the antibodies used to probe the immunoblot (IB) below. Only the interaction of FMRP with TBX3 (D) was affected by RNAse treatment. In all panels, the protein of interest is noted by the red arrowhead; the ∼50 kd band is IgG from the IP (black arrowhead). In all panels, the protein of interest is noted by the red arrowhead; the IgG band from the IP is labeled with a black arrowhead. K) Coomassie Blue stained PAGE gels of in vitro synthesized GST-DDX3, GST and MBP-GST. L) *In vitro* GST pull down assays: GST or GST-DDX3 affinity columns were incubated with. MBP-TBX3. Bound proteins were eluted, subjected to SDS-PAGE followed by IB with anti-TBX3 antibody.
(TIF)

Figure S2 Tbx3 mutant proteins are produced after transfection of expression plasmids and are efficiently immunoprecipitated. A) Anti-Tbx3 immunoblot of HEK293 lysates post transfection of Tbx3 constructs. Upper bands are full length proteins and the lower bands are degradation products (confirmed by MS). B) Immunoblot of IP'd HEK293 lysates. Upper bands are Tbx3 mutant proteins (red arrowheads show different size proteins); ∼50 kd bands are IgG (black arrowhead). Anti-goat IgG serves as a negative control for the IPs as the primary anti-Tbx3 IP antibody employed for this experiment was raised in goat. C) Production and IP of Tbx3ΔNLS protein in transfected HEK293 cells.
(TIF)

Figure S3 Tbx3 regulates alternative splicing of numerous targets in vivo. A–C) Tbx3 regulates alternative splicing in mouse limb buds *in vivo*. IGV screen shots comparing control anterior (CA1, CA2), *Tbx3;Prx1Cre* mutant anterior (MA1, MA2) forelimb bud RNA-Seq data for *Nfkb1* exons 5,6 (A), *Pus10* exons 7, 8 (B), and *Brca1* exon 8 (C). Red arrowheads indicate exons that are differentially spliced in a Tbx3-dependent manner. A′–C′) Schematics of splice variants and PCR primers used to detect them and RT-PCR validating variants. Anterior, anterior compartment; ctl1,control biologic replicate 1; mt1, mutant biologic replicate 1; ctl2,control biologic replicate 2; mt2, mutant biologic replicate 2.
(TIF)

Figure S4 RT-PCR testing of statistically significant versus insignificant splicing events identified by RNA-seq. A) RT-PCR testing of additional transcripts (listed at bottom of panels) from statistically significant alternate splice events in the anterior compartment (Tables S2, S3); all tested validated. B) RT-PCR testing of genes with statistically insignificant alternate splice events in the anterior compartment (Tables S2, S3); the events did not validate (i.e. no difference in mutant and control). C) Actin mRNA loading control. M, marker; Ctl, control; Mt, mutant.
(TIF)

Figure S5 Interaction of purified and endogenous TBX3 with RNA probes. A–F) EMSAs of MBP-conjugated TBX3 with radiolabeled RNA probe sequences listed at bottom of panels. red arrowhead: probe/protein complex; black arrowheads: unbound probe. G–H) Shift and supershift RNA EMSA assay of endogenous TBX3 in HEK293 cell lysates; radiolabeled probe sequences are listed below panels. Probes were incubated +/− HEK293 lysate, +/− anti-TBX3 antibody, and with the negative control antibody rabbit IgG (R-IgG). G) RNA probe containing GGUGU motifs. Probe/protein complex (lane 2) supershifts with Anti-TBX3 antibody (lanes 3) but not with R-IgG (lane 5). TBX3+RNA complexes are indicated by blue arrowhead, anti-Tbx3 antibody supershifted TBX3+RNA complexes are indicated by red arrowhead and unbound probe by black arrowhead. H) RNA probe containing AGGUGU motif from *Nfkb1* Fragment 3. Probe/protein complex (lanes 2, 3, 5) supershifts with Anti-TBX3 antibody (lane 2) but not with R-IgG (lane 5). TBX3+RNA complexes are indicated by blue arrowhead, anti-Tbx3 antibody supershifted TBX3+RNA complexes are indicated by red arrowhead and unbound probe by black arrowhead.
(TIF)

Table S1 TBX3 interacting proteins meeting MS screen criteria.
(DOCX)

Table S2 Validation results of randomly selected alternatively spliced target ordered by statistical significance.
(DOCX)

Table S3 RNA-Seq analysis of anterior limb compartment control versus Tbx3 mutant transcripts. Data and significance are provided for both expression fold change and splicing variants detected.
(XLSX)

Table S4 Location of putative TBEs and MEME motifs for the 11 validated alternative splicing events in the anterior limb compartment. Primary sequences of alternatively spliced exon and 1 kb of 5′ and 3′ flanking regions. Introns, exons, motifs and conservation are as noted in the KEY.
(DOCX)

Text S1 Supplemental Methods and Figure Legends. This supplement contains 2 sections: Supplemental Experimental Procedures and Supplemental Information References.
(DOCX)
