# Supplementary figures and images for: TBX3 Regulates Splicing In Vivo: A Novel Molecular Mechanism for Ulnar-Mammary Syndrome
Source: PLoS Genet. 2014 Mar 27;10(3):e1004247. doi: 10.1371/journal.pgen.1004247 (PMC3967948; doi:10.1371/journal.pgen.1004247)

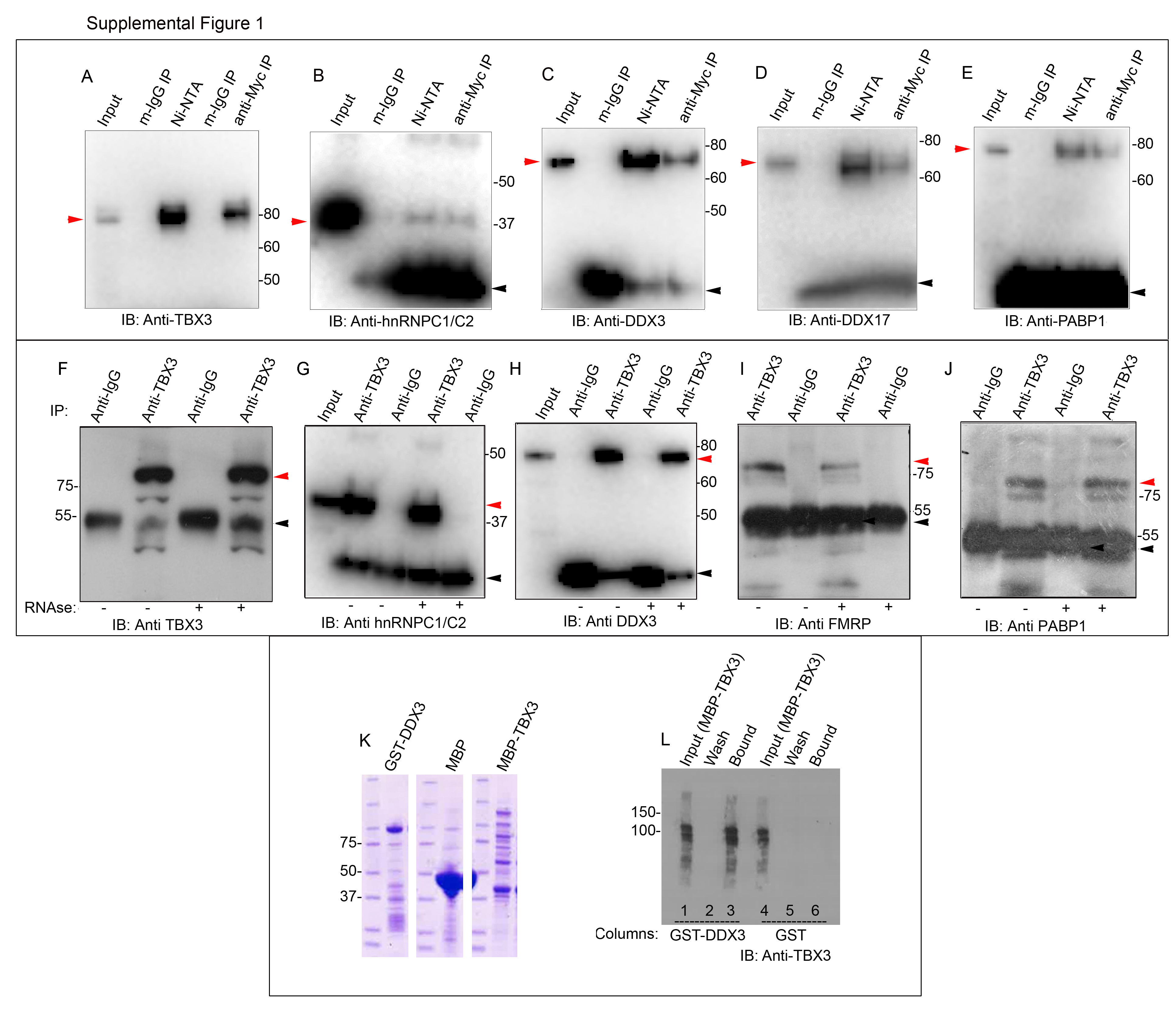

Supplement: Figure S1 — Dual-tagged pull down and immunoprecipitation assays and RNA independence of TBX3 interactions. A–E) Immunoblots of Ni-NTA eluate and anti-Myc immunoprecipitation products from HEK293 cells expressing dually (His-Myc tagged)-TBX3 to examine association with endogenous TBX3 interactors. The lanes labeled input contain the lysate prior to application to the Ni-NTA column; those labeled Ni-NTA contain the Ni-NTA eluate, whereas those labeled anti-Myc contain dialyzed Ni-NTA eluate post IP with anti-Myc. The mouse IgG IP is a negative control. Immunoblots were then probed with the antibodies listed below the panels (IB). Endogenous hNRNPC1/C2, DDX3, DDX17 and PABP1 are all bound, eluted with, and coIP with His-Myc-TBX3. F–J) HEK293 lysates +/− RNAse A treatment were immunoprecipitated with the antibody listed at the top of the panels (IP) and the antibodies used to probe the immunoblot (IB) below. Only the interaction of FMRP with TBX3 (D) was affected by RNAse treatment. In all panels, the protein of interest is noted by the red arrowhead; the ∼50 kd band is IgG from the IP (black arrowhead). In all panels, the protein of interest is noted by the red arrowhead; the IgG band from the IP is labeled with a black arrowhead. K) Coomassie Blue stained PAGE gels of in vitro synthesized GST-DDX3, GST and MBP-GST. L) In vitro GST pull down assays: GST or GST-DDX3 affinity columns were incubated with. MBP-TBX3. Bound proteins were eluted, subjected to SDS-PAGE followed by IB with anti-TBX3 antibody. (TIF) [file pgen.1004247.s001.tif]

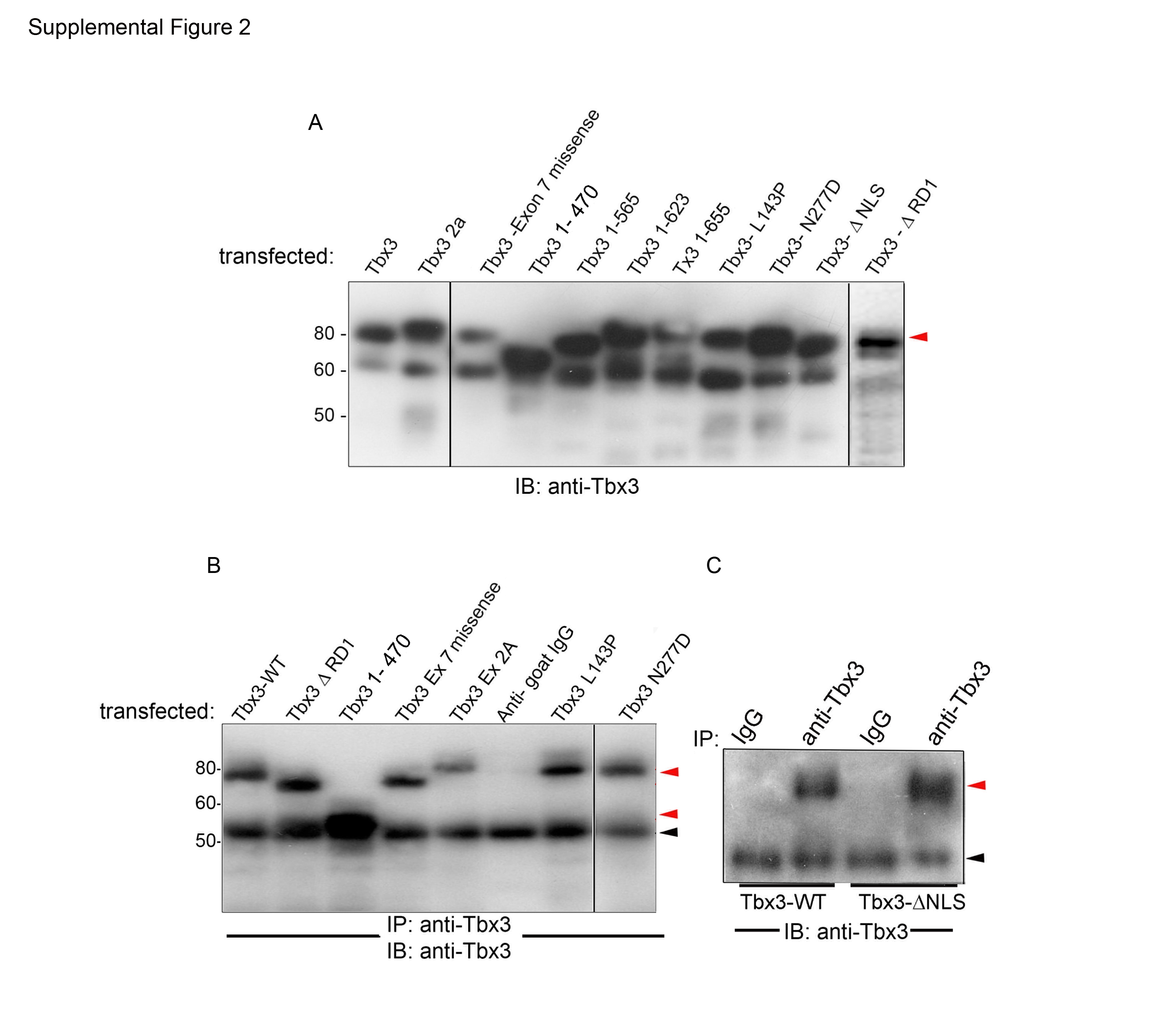

Supplement: Figure S2 — Tbx3 mutant proteins are produced after transfection of expression plasmids and are efficiently immunoprecipitated. A) Anti-Tbx3 immunoblot of HEK293 lysates post transfection of Tbx3 constructs. Upper bands are full length proteins and the lower bands are degradation products (confirmed by MS). B) Immunoblot of IP'd HEK293 lysates. Upper bands are Tbx3 mutant proteins (red arrowheads show different size proteins); ∼50 kd bands are IgG (black arrowhead). Anti-goat IgG serves as a negative control for the IPs as the primary anti-Tbx3 IP antibody employed for this experiment was raised in goat. C) Production and IP of Tbx3ΔNLS protein in transfected HEK293 cells. (TIF) [file pgen.1004247.s002.tif]

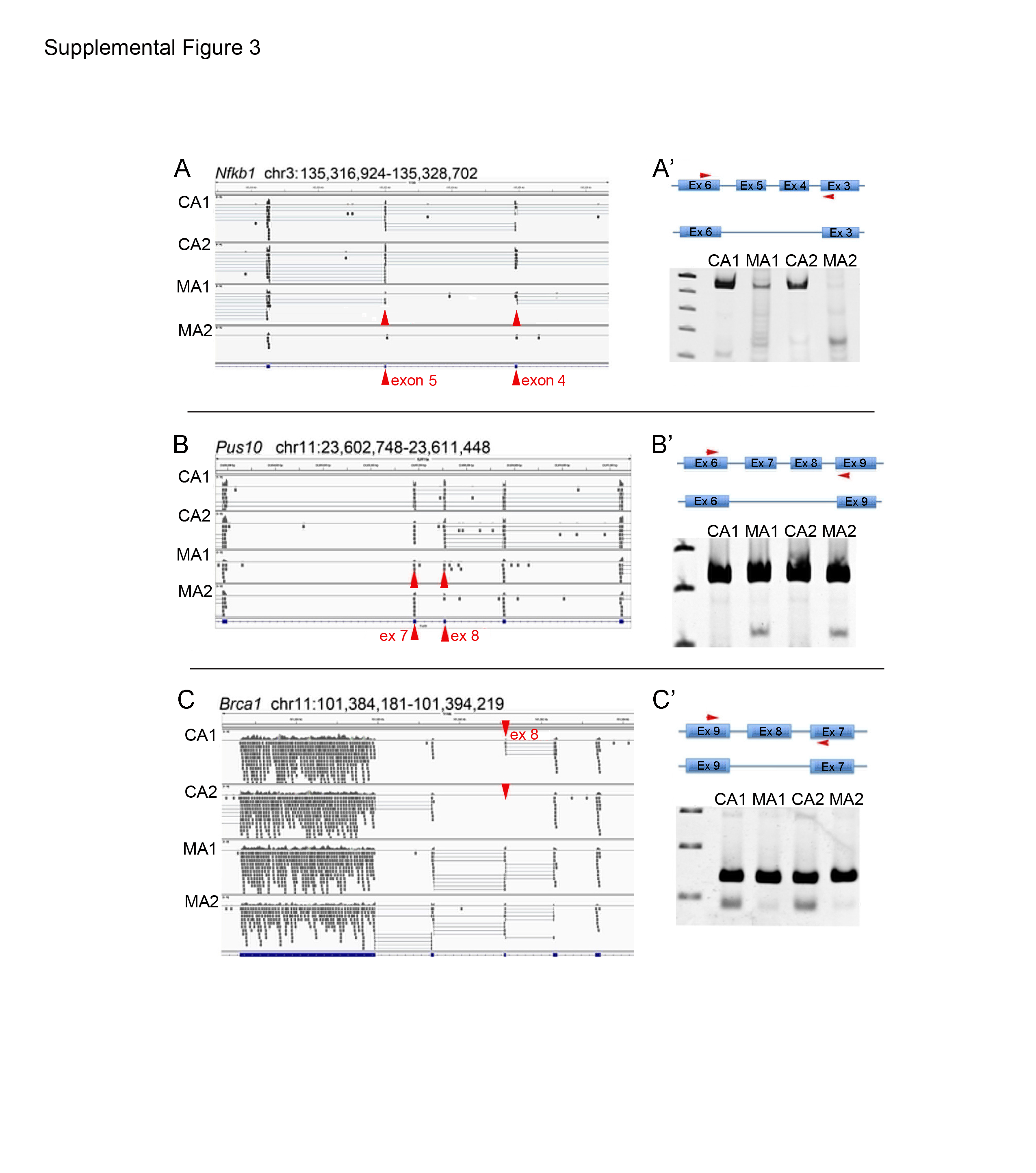

Supplement: Figure S3 — Tbx3 regulates alternative splicing of numerous targets in vivo. A–C) Tbx3 regulates alternative splicing in mouse limb buds in vivo. IGV screen shots comparing control anterior (CA1, CA2), Tbx3;Prx1Cre mutant anterior (MA1, MA2) forelimb bud RNA-Seq data for Nfkb1 exons 5,6 (A), Pus10 exons 7, 8 (B), and Brca1 exon 8 (C). Red arrowheads indicate exons that are differentially spliced in a Tbx3-dependent manner. A′–C′) Schematics of splice variants and PCR primers used to detect them and RT-PCR validating variants. Anterior, anterior compartment; ctl1,control biologic replicate 1; mt1, mutant biologic replicate 1; ctl2,control biologic replicate 2; mt2, mutant biologic replicate 2. (TIF) [file pgen.1004247.s003.tif]

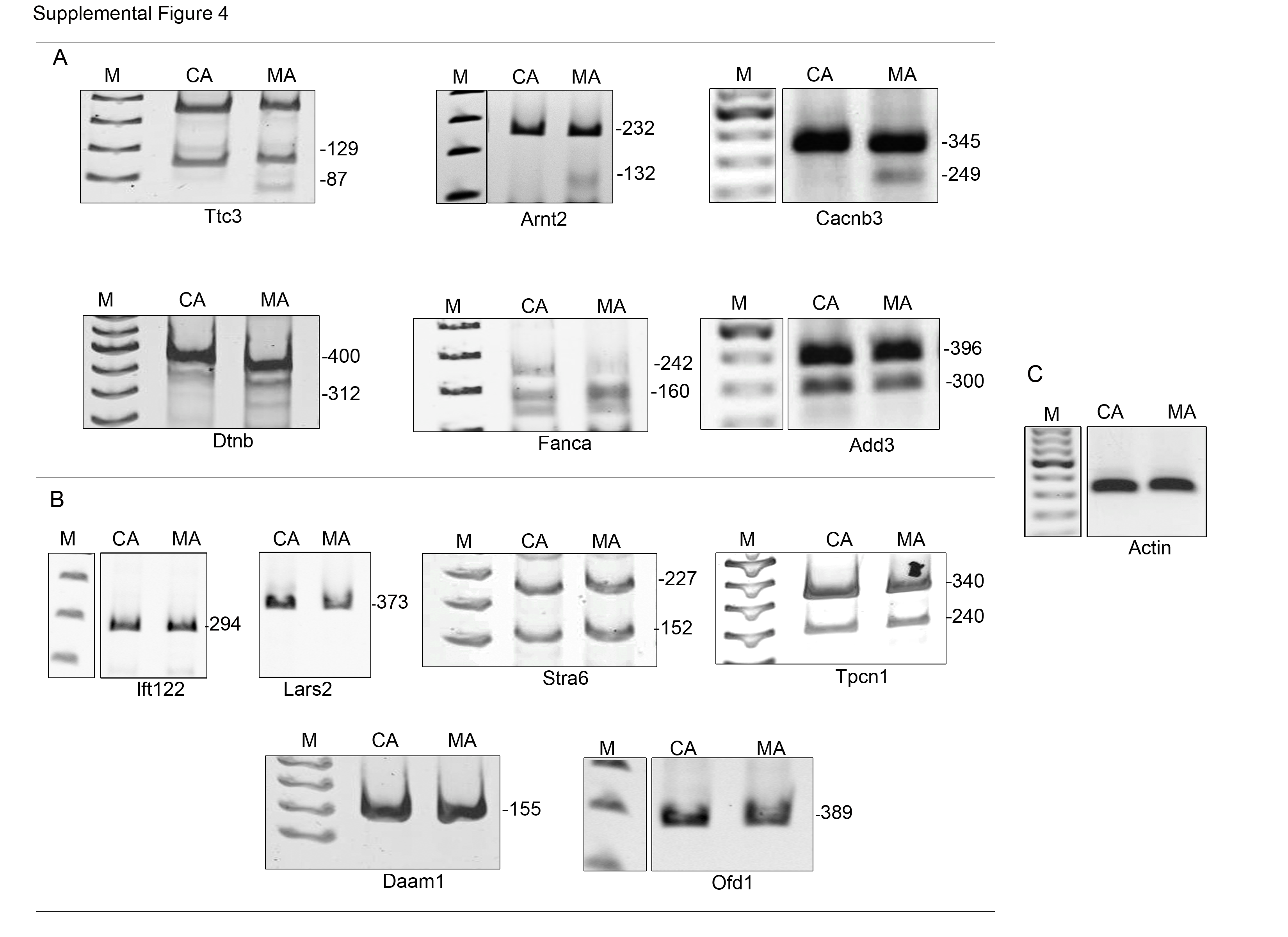

Supplement: Figure S4 — RT-PCR testing of statistically significant versus insignificant splicing events identified by RNA-seq. A) RT-PCR testing of additional transcripts (listed at bottom of panels) from statistically significant alternate splice events in the anterior compartment (Tables S2, S3); all tested validated. B) RT-PCR testing of genes with statistically insignificant alternate splice events in the anterior compartment (Tables S2, S3); the events did not validate (i.e. no difference in mutant and control). C) Actin mRNA loading control. M, marker; Ctl, control; Mt, mutant. (TIF) [file pgen.1004247.s004.tif]

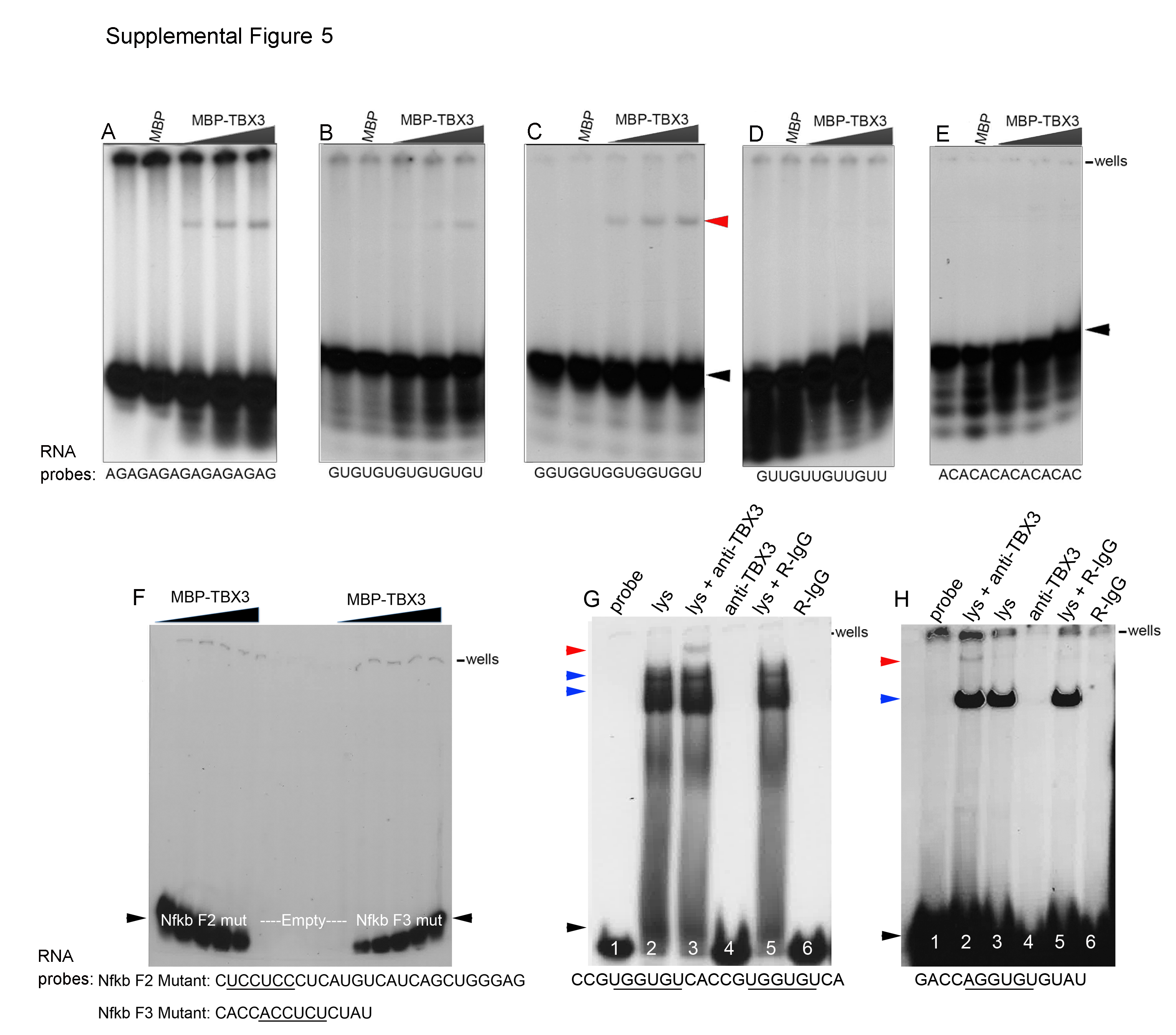

Supplement: Figure S5 — Interaction of purified and endogenous TBX3 with RNA probes. A–F) EMSAs of MBP-conjugated TBX3 with radiolabeled RNA probe sequences listed at bottom of panels. red arrowhead: probe/protein complex; black arrowheads: unbound probe. G–H) Shift and supershift RNA EMSA assay of endogenous TBX3 in HEK293 cell lysates; radiolabeled probe sequences are listed below panels. Probes were incubated +/− HEK293 lysate, +/− anti-TBX3 antibody, and with the negative control antibody rabbit IgG (R-IgG). G) RNA probe containing GGUGU motifs. Probe/protein complex (lane 2) supershifts with Anti-TBX3 antibody (lanes 3) but not with R-IgG (lane 5). TBX3+RNA complexes are indicated by blue arrowhead, anti-Tbx3 antibody supershifted TBX3+RNA complexes are indicated by red arrowhead and unbound probe by black arrowhead. H) RNA probe containing AGGUGU motif from Nfkb1 Fragment 3. Probe/protein complex (lanes 2, 3, 5) supershifts with Anti-TBX3 antibody (lane 2) but not with R-IgG (lane 5). TBX3+RNA complexes are indicated by blue arrowhead, anti-Tbx3 antibody supershifted TBX3+RNA complexes are indicated by red arrowhead and unbound probe by black arrowhead. (TIF) [file pgen.1004247.s005.tif]
