# Supplementary material for: TBX3 Regulates Splicing In Vivo: A Novel Molecular Mechanism for Ulnar-Mammary Syndrome
Source: PLoS Genet. 2014 Mar 27;10(3):e1004247. doi: 10.1371/journal.pgen.1004247 (PMC3967948; doi:10.1371/journal.pgen.1004247)
Supplement: Table S1 — TBX3 interacting proteins meeting MS screen criteria. (DOCX) [file pgen.1004247.s006.docx]

| CALX_HUMAN Calnexin OS=Homo sapiens GN=CANX PE=1 SV=2  SUPPLEMENTAL TABLE 1. TBX3 Interacting Proteins Identified by Mass Spectrometry and Passing Screen Criteria | | 2.88 | 30.22 | | 7.43 | | 67526.0 | P27824 | P35564 | X |  |  |
| --- | --- | --- | --- | --- | --- | --- | --- | --- | --- | --- | --- | --- |
| CAPR1_HUMAN Caprin-1 OS=Homo sapiens GN=CAPRIN1 PE=1 SV=2 | | 2.89 | 30.19 | | 4.80 | | 78318.2 | Q14444 | Q60865 | X | X | X |
| CH60_HUMAN 60 kDa heat shock protein, mitochondrial OS=Homo sapiens GN=HSPD1 PE=1 SV=2 | | 9.56 | 100.23 | | 21.47 | | 61016.5 | P10809 | P63038 | X | X |  |
| CLH1_HUMAN Clathrin heavy chain 1 OS=Homo sapiens GN=CLTC PE=1 SV=5 | | 2.83 | 30.18 | | 1.97 | | 191491.7 | Q00610 | Q68FD5 | X | X |  |
| CPSF6_HUMAN Cleavage and polyadenylation specificity factor subunit 6 OS=Homo sapiens GN=CPSF6 PE=1 SV=2 | | 6.59 | 70.22 | | 15.25 | | 59173.5 | Q16630 | Q6NVF9 | X |  |  |
| DDX17_HUMAN Probable ATP-dependent RNA helicase DDX17 OS=Homo sapiens GN=DDX17 PE=1 SV=1 * | | 10.78 | 120.21 | | 19.85 | | 72326.0 | Q92841 | Q501J6 | X | X | X |
| DDX3X_HUMAN ATP-dependent RNA helicase DDX3X OS=Homo sapiens GN=DDX3X PE=1 SV=3  Protein Description Sf XC Coverage M. Wt. Uniprot Uniprot  Score % HUMAN MOUSE H M SC | | 1.94 | 20.23 | | 3.63 | | 73198.1 | O00571 | Q62167 | X | X |  |
| DDX5_HUMAN Probable ATP-dependent RNA helicase DDX5 OS=Homo sapiens GN=DDX5 PE=1 SV=1 * | | 3.68 | 40.19 | | 7.33 | | 69104.8 | P17844 | Q61656 | X | X |  |
| DSRAD_HUMAN Double-stranded RNA-specific adenosine deaminase OS=Homo sapiens GN=ADAR PE=1 SV=4 | | 1.79 | 20.16 | | 2.69 | | 135981.0 | P55265 | Q99MU3 | X | X |  |
| ELOC_HUMAN Transcription elongation factor B polypeptide 1 OS=Homo sapiens GN=TCEB1 PE=1 SV=1 | | 1.35 | 20.16 | | 17.86 | | 12465.0 | Q15369 | P83940 | X |  |  |
| ENAH_HUMAN Protein enabled homolog OS=Homo sapiens GN=ENAH PE=1 SV=2 | | 10.45 | 110.32 | | 19.12 | | 66470.1 | Q8N8S7 | Q03173 | X | X |  |
| ENPL_HUMAN Endoplasmin OS=Homo sapiens GN=HSP90B1 PE=1 SV=1 | | 1.85 | 20.21 | | 3.11 | | 92411.2 | P14625 | P08113 | X | X |  |
| FBRL_HUMAN rRNA 2'-O-methyltransferase fibrillarin OS=Homo sapiens GN=FBL PE=1 SV=2 | | 8.06 | 86.31 | | 34.58 | | 33763.4 | P22087 | P35550 | X |  |  |
| FMR1_HUMAN Fragile X mental retardation 1 protein OS=Homo sapiens GN=FMR1 PE=1 SV=1 * | | 2.64 | 30.21 | | 8.79 | | 71130.9 | Q06787 | P35922 | X | X |  |
| FOP_HUMAN Friend of PRMT1 protein OS=Homo sapiens GN=C1orf77 PE=1 SV=2 | | 2.80 | 30.25 | | 11.69 | | 26380.3 | Q9Y3Y2 | Q9CY57 | X | X |  |
| GAR1_HUMAN H/ACA ribonucleoprotein complex subunit 1 OS=Homo sapiens GN=GAR1 PE=1 SV=1 | | 4.57 | 50.26 | | 24.88 | | 22334.2 | Q9NY12 | Q9CY66 | X |  |  |
| GRP75_HUMAN Stress-70 protein, mitochondrial OS=Homo sapiens GN=HSPA9 PE=1 SV=2 | | 7.57 | 80.22 | | 16.35 | | 73634.8 | P38646 | P38647 | X | X | X |
| GRP78_HUMAN 78 kDa glucose-regulated protein OS=Homo sapiens GN=HSPA5 PE=1 SV=2 | | 14.30 | 148.28 | | 27.52 | | 72288.5 | P11021 | P20029 | X | X | X |
| H2AY_HUMAN Core histone macro-H2A.1 OS=Homo sapiens GN=H2AFY PE=1 SV=4 | | 3.81 | 40.22 | | 18.82 | | 39592.5 | O75367 | Q9QZQ8 | X |  | X |
| H2B1B_HUMAN Histone H2B type 1-B OS=Homo sapiens GN=HIST1H2BB PE=1 SV=2* | | 0.98 | 10.25 | | 11.90 | | 13941.6 | P33778 | Q64475 | X | X | X |
| H4_HUMAN Histone H4 OS=Homo sapiens GN=HIST1H4A PE=1 SV=2 | | 1.87 | 20.18 | | 21.36 | | 11360.4 | P62805 | P62806 | X | X |  |
| HNRCL_HUMAN Heterogeneous nuclear ribonucleoprotein C-like 1 OS=Homo sapiens GN=HNRNPCL1 PE=1 SV=1 | | 3.44 | 40.20 | | 10.92 | | 32122.7 | O60812 | N/A | X |  |  |
| HNRH1_HUMAN Heterogeneous nuclear ribonucleoprotein H OS=Homo sapiens GN=HNRNPH1 PE=1 SV=4* | | 1.38 | 16.20 | | 4.68 | | 49198.4 | P31943 | O35737 | X | X | X |
| HNRH3_HUMAN Heterogeneous nuclear ribonucleoprotein H3 OS=Homo sapiens GN=HNRNPH3 PE=1 SV=2 | | 6.21 | 70.28 | | 27.17 | | 36903.0 | P31942 | D3Z6Y3 | X |  |  |
| HNRL2_HUMANHeterogeneous nuclear ribonucleoprotein U-likeprotein2OS=Homo sapiens GN=HNRNPUL2 PE=1SV=1* | | 1.96 | 20.23 | | 3.61 | | 85052.2 | Q1KMD3 | Q00PI9 | X | X |  |
| HNRPC_HUMAN Heterogeneous nuclear ribonucleoproteins C1/C2 OS=Homo sapiens GN=HNRNPC PE=1 SV=4 * | | 2.75 | 30.25 | | 8.82 | | 33649.6 | P07910 | Q9Z204 | X | X | X |
| HNRPK_HUMAN Heterogeneous nuclear ribonucleoprotein K OS=Homo sapiens GN=HNRNPK PE=1 SV=1 * | | 4.81 | 50.29 | | 15.98 | | 50944.4 | P61978 | P61979 | X | X |  |
| HNRPM_HUMAN Heterogeneous nuclear ribonucleoprotein M OS=Homo sapiens GN=HNRNPM PE=1 SV=3 | | 10.97 | 118.20 | | 14.52 | | 77464.3 | P52272 | Q9D0E1 | X | X |  |
| HNRPU_HUMAN Heterogeneous nuclear ribonucleoprotein U OS=Homo sapiens GN=HNRNPU PE=1 SV=6 * | | 13.87 | 150.28 | | 15.39 | | 90528.0 | Q00839 | Q8VEK3 | X | X |  |
| HS71L_HUMAN Heat shock 70 kDa protein 1-like OS=Homo sapiens GN=HSPA1L PE=1 SV=2 | | 7.70 | 78.22 | | 12.48 | | 70331.5 | P34931 | P16627 | X |  |  |
| HS90A_HUMAN Heat shock protein HSP 90-alpha OS=Homo sapiens GN=HSP90AA1 PE=1 SV=5 | | 2.89 | 28.23 | | 5.74 | | 84606.7 | P07900 | P07901 | X | X | X |
| HS90B_HUMAN Heat shock protein HSP 90-beta OS=Homo sapiens GN=HSP90AB1 PE=1 SV=4 | | 1.93 | 20.24 | | 4.14 | | 83212.2 | P08238 | P11499 | X |  | X |
| HSP71_HUMAN Heat shock 70 kDa protein 1A/1B OS=Homo sapiens GN=HSPA1A PE=1 SV=5 | | 5.79 | 60.27 | | 11.54 | | 70009.2 | P08107 | Q61696 | X |  |  |
| HSP72_HUMAN Heat shock-related 70 kDa protein 2 OS=Homo sapiens GN=HSPA2 PE=1 SV=1 | | 2.83 | 30.21 | | 4.38 | | 69978.0 | P54652 | P17156 | X | X | X |
| HSP7C_HUMAN Heat shock cognate 71 kDa protein OS=Homo sapiens GN=HSPA8 PE=1 SV=1 | | 14.51 | 160.25 | | 18.11 | | 70854.4 | P11142 | P63017 | X | X | X |
| IMA2_HUMAN Importin subunit alpha-2 OS=Homo sapiens GN=KPNA2 PE=1 SV=1 | | 4.75 | 48.25 | | 13.99 | | 57826.1 | P52292 | P52293 | X | X |  |
| IMB1_HUMAN Importin subunit beta-1 OS=Homo sapiens GN=KPNB1 PE=1 SV=2 | | 4.77 | 50.24 | | 7.99 | | 97108.2 | Q14974 | P70168 | X | X |  |
| KIF7_HUMAN Kinesin-like protein KIF7 OS=Homo sapiens GN=KIF7 PE=1 SV=2 | | 4.67 | 50.18 | | 4.17 | | 150494.3 | Q2M1P5 | B7ZNG0 | X | X |  |
| LAP2A_HUMAN Lamina-associated polypeptide 2, isoform alpha OS=Homo sapiens GN=TMPO PE=1 SV=2 | | 2.90 | 30.25 | | 6.63 | | 75445.8 | P42166 | Q61033 | X | X |  |
| LMNB1_HUMAN Lamin-B1 OS=Homo sapiens GN=LMNB1 PE=1 SV=2 | | 2.83 | 30.19 | | 6.14 | | 66367.7 | P20700 | P14733 | X | X |  |
| MYH9_HUMAN Myosin-9 OS=Homo sapiens GN=MYH9 PE=1 SV=4 | | 5.68 | 60.23 | | 3.52 | | 226390.6 | P35579 | Q8VDD5 | X | X | X |
| NSF_HUMAN Vesicle-fusing ATPase OS=Homo sapiens GN=NSF PE=1 SV=3 | | 4.77 | 50.22 | | 8.06 | | 82542.2 | P46459 | P46460 | X |  |  |
| PABP1_HUMAN Polyadenylate-binding protein 1 OS=Homo sapiens GN=PABPC1 PE=1 SV=2 * | | 5.68 | 60.25 | | 11.01 | | 70626.0 | P11940 | P29341 | X | X |  |
| PARP1_HUMAN Poly [ADP-ribose] polymerase 1 OS=Homo sapiens GN=PARP1 PE=1 SV=4 | | 15.93 | 168.27 | | 15.48 | | 113012.4 | P09874 | P11103 | X | X |  |
| PCBP1_HUMAN Poly(rC)-binding protein 1 OS=Homo sapiens GN=PCBP1 PE=1 SV=2 | | 4.76 | 50.24 | | 20.79 | | 37474.0 | Q15365 | P60335 | X | X |  |
| PCBP2_HUMAN Poly(rC)-binding protein 2 OS=Homo sapiens GN=PCBP2 PE=1 SV=1 | | 5.69 | 60.21 | | 24.93 | | 38555.6 | Q15366 | Q61990 | X |  |  |
| PDIA4_HUMAN Protein disulfide-isomerase A4 OS=Homo sapiens GN=PDIA4 PE=1 SV=2 | | 2.71 | 30.18 | | 6.20 | | 72887.1 | P13667 | P08003 | X |  |  |
| PDIP3_HUMAN Polymerase delta-interacting protein 3 OS=Homo sapiens GN=POLDIP3 PE=1 SV=2 | | 3.69 | 40.19 | | 14.73 | | 46060.6 | Q9BY77 | Q8BG81 | X |  |  |
| PHB2_HUMAN Prohibitin-2 OS=Homo sapiens GN=PHB2 PE=1 SV=2 | | 3.80 | 40.18 | | 15.05 | | 33275.9 | Q99623 | O35129 | X | X | X |
| PRKDC_HUMAN DNA-dependent protein kinase catalytic subunit OS=Homo sapiens GN=PRKDC PE=1 SV=3 | | 21.16 | 228.22 | | 5.67 | | 468786.9 | P78527 | P97313 | X | X |  |
| PTBP1_HUMAN Polypyrimidine tract-binding protein 1 OS=Homo sapiens GN=PTBP1 PE=1 SV=1* | | 0.96 | 10.22 | | 3.01 | | 57185.8 | P26599 | P17225 | X | X | X |
| RA1L2_HUMAN Heterogeneous nuclear ribonucleoprotein A1-like 2 OS=Homo sapiens GN=HNRNPA1L2 PE=2 SV=2 | | 9.46 | 98.28 | | 30.31 | | 34204.3 | Q32P51 | B7ZWG9 | X |  |  |
| RAB1A_HUMAN Ras-related protein Rab-1A OS=Homo sapiens GN=Rab1A PE=1 SV=3 | | 1.72 | 20.18 | | 16.10 | | 22663.4 | P62820 | P62821 |  | X | X |
| RAVR1_HUMAN Ribonucleoprotein PTB-binding 1 OS=Homo sapiens GN=RAVER1 PE=1 SV=1 | | 1.88 | 20.21 | | 5.78 | | 63837.3 | Q8IY67 | Q9CW46 | X |  |  |
| RBM14_HUMAN RNA-binding protein 14 OS=Homo sapiens GN=RBM14 PE=1 SV=2 | | 11.14 | 120.27 | | 22.87 | | 69449.0 | Q96PK6 | Q8C2Q3 | X |  |  |
| RFC4_HUMAN Replication factor C subunit 4 OS=Homo sapiens GN=RFC4 PE=1 SV=2 | | 1.91 | 20.19 | | 6.61 | | 39656.9 | P35249 | Q99J62 | X |  |  |
| RL11_HUMAN 60S ribosomal protein L11 OS=Homo sapiens GN=Rpl11 PE=1 SV=4* | | 0.92 | 10.18 | | 7.87 | | 20239.7 | P62913 | Q9CXW4 |  | X |  |
| RL12_HUMAN 60S ribosomal protein L12 OS=Homo sapiens GN=Rpl12 PE=1 SV=2 | | 2.74 | 30.18 | | 28.48 | | 17793.5 | P30050 | P35979 |  | X |  |
| RL23_HUMAN 60S ribosomal protein L23 OS=Homo sapiens GN=Rpl23 PE=1 SV=1 | | 1.91 | 20.18 | | 25.00 | | 14856.1 | P62829 | P62830 |  | X |  |
| RL40_HUMAN Ubiquitin-60S ribosomal protein L40 OS=Homo sapiens GN=UBA52 PE=1 SV=2* | | 0.97 | 10.20 | | 12.50 | | 14719.0 | P62987 | P62984 | X | X |  |
| RLA0L_HUMAN 60S acidic ribosomal protein P0-like OS=Homo sapiens GN=RPLP0P6 PE=5 SV=1 | | 2.86 | 30.20 | | 13.88 | | 34342.7 | Q8NHW5 | N/A | X |  |  |
| ROA1_HUMAN Heterogeneous nuclear ribonucleoprotein A1 OS=Homo sapiens GN=HNRNPA1 PE=1 SV=5 | | 3.91 | 40.32 | | 14.52 | | 38723.0 | P09651 | P49312 | X |  |  |
| ROA2_HUMAN Heterogeneous nuclear ribonucleoproteins A2/B1 OS=Homo sapiens GN=HNRNPA2B1 PE=1 SV=2 | | 22.19 | 236.34 | | 47.31 | | 37406.7 | P22626 | O88569 | X | X |  |
| ROA3_HUMAN Heterogeneous nuclear ribonucleoprotein A3 OS=Homo sapiens GN=HNRNPA3 PE=1 SV=2 | | 18.17 | 190.31 | | 40.48 | | 39570.5 | P51991 | Q8BG05 | X |  |  |
| RS3_HUMAN 40S ribosomal protein S3 OS=Homo sapiens GN=RPS3 PE=1 SV=2 | | 4.66 | 50.18 | | 23.05 | | 26671.4 | P23396 | P62908 | X |  |  |
| RS4X_HUMAN 40S ribosomal protein S4, X isoform OS=Homo sapiens GN=RPS4X PE=1 SV=2 | | 10.55 | 110.28 | | 34.98 | | 29579.1 | P62701 | P62702 | X | X | X |
| RSSA_HUMAN 40S ribosomal protein SA OS=Homo sapiens GN=Rpsa PE=1 SV=4 | | 1.91 | 20.23 | | 10.17 | | 32817.5 | P08865 | P14206 | X | X | X |
| TCPA_HUMAN T-complex protein 1 subunit alpha OS=Homo sapiens GN=TCP1 PE=1 SV=1 | | 3.74 | 40.24 | | 8.63 | | 60305.7 | P17987 | P11983 |  | X | X |
| TIF1B_HUMAN Transcription intermediary factor 1-beta OS=Homo sapiens GN=TRIM28 PE=1 SV=5 | | 2.74 | 30.17 | | 5.15 | | 88493.5 | Q13263 | Q62318 | X | X |  |
| TNPO1_HUMAN Transportin-1 OS=Homo sapiens GN=TNPO1 PE=1 SV=2 | | 1.82 | 20.15 | | 2.45 | | 102288.9 | Q92973 | Q8BFY9 | X | X |  |
| TRAP1_HUMAN Heat shock protein 75 kDa, mitochondrial OS=Homo sapiens GN=TRAP1 PE=1 SV=3 | | 1.95 | 20.27 | | 4.97 | | 80059.8 | Q12931 | Q9CQN1 | X |  |  |
| UBP2L_HUMAN Ubiquitin-associated protein 2-like OS=Homo sapiens GN=UBAP2L PE=1 SV=2 | | 3.73 | 40.20 | | 5.06 | | 114465.4 | Q14157 | Q80X50 | X | X |  |
| VIME_HUMAN Vimentin OS=Homo sapiens GN=VIM PE=1 SV=4 | | 13.99 | 150.27 | | 35.62 | | 53619.2 | P08670 | P20152 | X | X |  |
| XPO1_HUMAN Exportin-1 OS=Homo sapiens GN=XPO1 PE=1 SV=1 | | 11.28 | 120.22 | | 10.18 | | 123306.1 | O14980 | Q6P5F9 | X |  |  |
| XRCC6_HUMAN X-ray repair cross-complementing protein 6 OS=Homo sapiens GN=XRCC6 PE=1 SV=2 | | 3.73 | 40.20 | | 9.03 | | 69799.2 | P12956 | P23475 | X |  |  |
| ­­­­­N/A - indicates human proteins with no mouse ortholog |  | | |  | |  |  |  |  |  |  |  |
| # - indicates Tbx3 interacting protein confirmed by a complimentary method; see Figure 3 |  | | |  | |  |  |  |  |  |  |  |
| * - indicates protein with XC score < 20 and therefore manually inspected to confirm identification by mass spectrometry | | | | | |  |  |  |  |  |  |  |
