# Supplementary material for: TBX3 Regulates Splicing In Vivo: A Novel Molecular Mechanism for Ulnar-Mammary Syndrome
Source: PLoS Genet. 2014 Mar 27;10(3):e1004247. doi: 10.1371/journal.pgen.1004247 (PMC3967948; doi:10.1371/journal.pgen.1004247)
Supplement: Table S2 — Validation results of randomly selected alternatively spliced target ordered by statistical significance. (DOCX) [file pgen.1004247.s007.docx]

**Supplemental Table 2A. Statistically significant exon alternative splicing events tested.**

| **Gene, tissue** | **AltSpliceChiSqrPVal** | **AltSpliceMaxLog2Ratio** | **result** |
| --- | --- | --- | --- |
| Arnt2,anterior | 39.9 | -2.69 | validated |
| Wdr70, anterior | 36.2 | 2.63 | validated |
| Dlg3, posterior | 31.03 | -9.01 | validated |
| Dlg3, anterior | 26.6 | -2.79 | validated |
| Pus10, anterior | 26.3 | -2.15 | validated |
| Nfkb1 ex11, posterior | 23.9 | -7.55 | validated |
| Brca1, anterior | 23.2 | 2.26 | validated |
| Add3, anterior | 22.1 | -1.41 | validated |
| Ttc3, anterior | 14.5 | -2.38 | validated |
| Dtnb, anterior | 13.9 | -1.74 | validated |
| Fanca, anterior | 13.4 | -1.77 | validated |
| Cacnb3, anterior | 13.4 | -1.74 | validated |

**Supplemental Table 2B. Statistically insignificant exon alternative splicing events tested.**

| **Gene, tissue** | **AltSpliceChiSqrPVal** | **AltSpliceMaxLog2Ratio** | **result** |
| --- | --- | --- | --- |
| Ift122 anterior | 12.6 | -2.32 | did not  validate |
| Stra6 anterior | 12.3 | -2.25 | did not  validate |
| Tpcn1 anterior | 10.6 | 2.13 | did not  validate |
| Daam1 anterior | 10.1 | -2.92 | did not  validate |
| Ofd1, anterior | 6.9 | -2.24 | did not  validate |
| Lars2, anterior | 1.2 | -2.19 | did not  validate |
| Nfkb1 ex11 anterior | 0 | -1.65 | **validated** |
| Nfkb1 ex 5, 6 anterior | 0 | -1.65 | **validated** |
