# Supplementary material for: TBX3 Regulates Splicing In Vivo: A Novel Molecular Mechanism for Ulnar-Mammary Syndrome
Source: PLoS Genet. 2014 Mar 27;10(3):e1004247. doi: 10.1371/journal.pgen.1004247 (PMC3967948; doi:10.1371/journal.pgen.1004247)
Supplement: Table S4 — Location of putative TBEs and MEME motifs for the 11 validated alternative splicing events in the anterior limb compartment. Primary sequences of alternatively spliced exon and 1 kb of 5′ and 3′ flanking regions. Introns, exons, motifs and conservation are as noted in the KEY. (DOCX) [file pgen.1004247.s009.docx]

**Supplemental Table 4.** Motif analysis of 11 statistically significant, validated alternatively spliced genes in the anterior limb compartment.

KEY

Regular font: intron

*Italics*: alternatively splice exon

**BOLD**: consensus TBE

Red: conservation

Underlined: MEME motif/Sine

>dlg3

>mm9_dna range=chrX:98006713-98009482 5'pad=0 3'pad=0 strand=+ repeatMasking=none

GTAGGCCCTCCTGTGGCCTGACTTGGTTGGCCGCTCGCTGTCTCACTCCT

GGCGCATTTGAGTTTGTGCTGCATGTGACCTCTCAG**TGGTGTG** conserved to shrew

TTCTGAATTGGAGTCTCTTTTTTGTTGGC

*TTGCAGAGGGAGTGACATCC****AACACCA*** conserved to zebrafish *GTGACAGCGAAAGCAGTTCCAGTAAG*

TGTGTGTCATGCCCCTGTGTCCTGT

AGCTTCCCATGTCTCCCTGCC**ATCACCA**TTCCAGTGTCCACCACTGCCAT

CACCACGCAGAGGCTGCTGCTCCTTTTAGCCATGTATACTTTGAGCCCCT

GGAGATGCTGTGGCCAGCTCATTCACCTTGTGGGCTGGAATTTGGAGATT

AGAGGGAGAGAATAAGCCCTATAGCTCTAGCTAGGACCTAGCTTGAAACC

ATCTCTGGGGTGCTGGGAAGGATAAACTGCCACTGATCAGGTCCCTGACA

TGGGCTTCTAGGCTGCTAAGACTATCAGCTGGACAGATTTGCTCAGAGAC

CACAAGTAAAATGGCTTCTGCCTTCCATCTGCCTTTCTGGGCTCCTAGG**A**

**CCACCT**TACCTTCTGGCTGGGTTACATAGTCAGGAAAGGAATTGTCCCAA

ATGCCTTGGGAACTCCTATGACTACAGCATTGAAGCCATAGTTCAAATCT

CACACGCTCTGGGAACACTGGTCTTTGAGTCTCATGTTCAGGAGCATCCC

TGTAGCAAGTATCCTGTGGCTCCATGCAGCTCTTGCTGCCTTCCCTAGGA

CTGCTGTCAGCTCATTTGGGAACCATAGGAACCCAACACCAAGTGTCTTG

GGGACTTTTTTTAGGAAGGCCTCATCATTGCCAGGTCACATGGGGGTTTA

CCCATGAGCAGAGATGAGGCTCAGTATTGCTCTTAAAGGATGCTGGGACT

TTAGCTCAGGCATGCACCAGTTTCCTGGATGCTTCTTAGAGATGTGATGT

AGGTAAATAAATGGACCTTTAAAGGGGCATCATTAGGCTATAGTTTTGGA

AGTTGGGCCTAAGGGTCTGGACTCAGGTAAAGTGACTCTGC**AGGTGTG**TT

TCAACCAGCTGGAGCTTCTCTTGATTCTTCTCAGAACAAGGGGGCTATTG

AATATAACTATTACCTATAACAAGTAACTCCCTAGAGCTTGGGGTGGTTT

GGTGGAGACTCGACTTATGGGCCTAGCCCCATAATTGGGCAATATTTCAC

TGAATGAAAACAGCCCAGCCCAAGGCTGCTGAATGGCTCCTTAGCCTTCT

TGCCAACAGAAAGTGAGCCTGTTTTGTGGTGGGCGTGG**GGTGTG**GT conserved to dog

GAATGCTGTACACAGTCATCAGATCATAAAGTGCTGACGTGTTTCCCTTTTTGC

*AGAAGGACAAGAGGATGCTATTTTGTCATATGAGCCAGTGACACGACAAG*

*AAA*

GTAAGCCCCCTTCTGAGAGTCTTGCCTTTGTGCTAGTCTCCCGGTCA

TGGGGCTTAGTAAAGAGGATAGCTTGGGGAGCAGGGAAGGAGGAGGAGGC

ACATCCAGCCCCTGGCTCATCCCTAGCATCACTGGATGGATGTCATGCTG

CA**CACACCT**CTTGTGATAATTTTGCCCACTGCTAGGATGCCTTCCAAAGG

GTGGGTGGATTTCAGAGGAAAGCAGGGCAAGGCCTTTTTCAGCAGAGGGG

GTTGTTGCTGCCCAGGCAAAGGACCTGTATTCTCCCCTGGGCATCTGCTT

TGCAAGAACCTTGCTTAGTAGCTAGGGTCTGTGTGAAACTTGCTCCCGAA

TCTGTTGGAAATGGAAAGGCTTCTGATTTCAGACTTGCCCGAGACTTTAC

ATTGTCAAATTTAAAGGCTCTTTGAAGTCTTTAATTAAAAGCCAGAGAGT

AACCATAGTTGCTTCACCTTGTCTGAGTGGTGGCCACCGAGCCTCTGTCC

ACGGAGGTGGGGCAGGTTGTCTTGTGGGAGCTCTGTTCACTGTCTTGTCC

TCTCCTTCCCCAG*TTCACTATGCCAGGCCTGTGATCATCTTGGGCCCAATGAAGGACCGAGTCAACGATGACCTCATCTCTGAGTTCCCGCATAAATTTGGATCCTGTGTGCC*

*AC*GTAAGAGCCCAGGCAGACTCAGATGAGTCAGGTTT

GCTACTCTTGGAAGTGCTACTTTTTCAAAAGTGTCTAGGATCAGGGCCAT

GTGGGACCTCCTGGAAGAGAGTAGGTAGCAATGGATAACCCCAGAGTAAG

TGGTATTGGTAAGTTTGGCATCCAACTTGGCTGTTTGCCTCATGCTGTGA

AGTTAAACTTTGGGTGGTGATGGTCAGGGTTGATTTTACAAACCCATCAT

TGTTTTGGTTTTTGTTTCCTTGTGCTAGGAATTAAACAAAGTGGTTTGAG

GAAGCATTTCTTGGTTTTTTGTTTGTTTGTTTGTTTGTGTTTTGGGTTTT

TTTTGAGACAGGGTTTCTCTGTAGCCCTGGGTGTCCTGGAACTCACTCTG

TAGACCAGGTTGGCCTTGAACTCAGAAATCCGCCAGCCTCTGCCTCCCAA

GTGCTAGGATTAA**AGGTGTG**CATCACCACCGCTCGGCTGTTTCTTGGGCT

TCTTAAATGCCAAGTATTGCATCCTTTACCAGGCCATTGCTCAGGGAGCC

CCACCTTTTTGGTCCATATTACCCTCAAGGACCCTGTGTATAATATCTGG

AGTTTGGAAGAATAAATAGGCCAGACAAAATCTTTCCGAGTGTTTTACTC

ATTCATCTGAAAAATATTGGAGTAGCCGTGTGTTCTAGCCTAGTGAGAAA

GGATGAAGTTGGCTTTTAAT

>ttc3

>mm9_dna range=chr16:94591304-94593757 5'pad=0 3'pad=0 strand=+ repeatMasking=none

GGTCCTTCCTGACTTATTCTGATAGAACTGCCAGGGAGTGGCCTGGAGGT

AGCTAACCCCAATTTCTAAACCTTCCAAGGCTCTTCCAGGGCACTCATCA

TGCTCTCTGAACTGTGGGTTTACTATGCTTCTCCACATGACTATGACCTC

AAGGATTGGGGCCAGGTTTTATTCACATTAGCATCAAGCCTAGTGCTAAA

TAAATATTGGAGAGTTCATCTAGAACTGCATACATAAACTGAGCAAACAA

ACGGTACCCCTTTTAGGACTCGTGAAGAGACACAGGTAGAAAGAATCAAC

AGTTCATGTGTGTACGTTCTAAGGACAAACACATTATTTCCTGGTTCTCA

ATACCATTAAGGGCCTTGGCCATCCATCATTCCAGTACTTACTGAAGCCA

AATTGTGAGCTTAGGAAAAGAACAAAGCCATAAATCGCTCTTTCTGGCAA

TGGCGACGGTGAATTTTCCACCATCTTCCCTGTGGCTTTAGACAATCTGT

GGAAAAGGAACACAATCGTCGTCAGACATCTCCCCCCGCCCCCCTTCACC

AATGGTCTGTTACATAATCTCATCGCTTGCTGCGGACACAGCCACTGCGG

ATGTTCGCGCGCGCGCGCCACTGCAGCTGAGGGGGCGGCGGGAGCGCGCG

CTGCGGGGAAAATGCGCACCTCGGACCCTCTGAGGCGAGGAAGGGGCCCG

GGCTGGACAGAGGCCCCGAGTCCTGCCCTCTCATGATGGGCCCTGTCCCA

GCCTTCGTGAGGCTCAGAGGAAACAGGAGGCCCGGGACCTTGAGTCCTGC

GAGCACCGACGCCCACCCGTCCCCACCCTTCAGCCCCACTCAGTCCAATG

CGCAGGCGCGGCTCAGCTACCCCCGCAGCGTGGGCGGAAATTAAAAAAAA

AAAAAAACCTGAGGAAAAGCAGAGGCTGGCAGGCGCCCCCGCCGCTTGGC

GCAGGCGCACCGCGACCGCAGCGAGGGGGCTAGCACGG**TCTCACCA**CCCA

CCCCACCGAGCTGAGGGGGCGACGCGGCGACTGCGCATGCGC

*ACCCTAAGCCACGTGACCCGCCCGCCTCAGGAGCCGCGGAGCGTTCGCTCT*

*GCGGGTCTGGTTGACGTGGAGGGCCGGAGGCGTCGGCAGCCCGCGTCTGAG*

*GCTCGCGGGCTGCGGGCCCGGGTG*

AGTGCAGACCCTGCGCGCGGCGGGGCTCACGG

AAGCGTCTGCGCGTCCCGCCGCAGCCGAGATGCCCGGGCATCGGGGCGCT

C**CACACCT**CCCTCCGTG**GGTGTGTGGTG**AGTGTG**GGTGTG** conserved to human CGCGTCGCCG

CGCGTCTCGCCGAGCGCCCGCGCGCTGAGGGGGTGTGGCCGCCCTTGCCC

TTGTGGGTGTGTTCCCTTTGTCCAGTGACCCTCGGGCGTCCTTAACGGTA

GAAGGGGGGTCTTTTGTGTGCGCGTCGCCTGGCGTGCGGGTGACATCCCG

GGTCAGGTACCCGTTCCCGCGTTCCGAGCGCTCGGGGACGCGCCTCTGGG

TCTGCGGCTGGCCCGCTTTGTTCCTACGCTCTCCCTTCCCTACGACGGCG

TGGGCGTCCTTCTCATCCTCTGAGGATGGTGGTTCCCCTCCCACCTTTGT

CACTGCCTTCCCTG**TGGTGTG**CGCGCCCCCTCCTCAGCACCCGCGCGCGC

CCGCCCACCCGCCGCTGCGGCAGGCTGCAGTGGCAGGCGCGCGCGTGCGT

CCTCGAGGGTCCCCAAAGGTGCGCTGTGTAGGGGCCCTGTGGCGTGCGCG

GGGTTGGAATTGCGGTGACGCGCGTGCGCGCTGCCCCTGGTCTGTGTGTG

CGCGCCTCTCCCCCCCACCCCCACCCCCGTTTCTGGGAGGACCCGGGAGG

GACGCAGAGCTGCCCCCCACCCCACCTCCCCACCTGCAGCCTTTTCCTAC

GCCTGCGCAGGGCTGCTGCTCCGCAGTGCTGGCGACGTCACTGAGGCCTG

GGCTGCCATCTCCTATTGGGGAGGGGACAGACTAGGGGGGTGGGGGGACA

AAGGACCAAAATGAAGCCGAGGGTGTGTCGGTACCACTGCACGGTACCTT

TCTCAGAAGCTTGTAGTTTCCAGACGTCCTTCAGTTTGCAAATTCTTTCC

ACCGTTTGTGCTTGTGCC**TCTCACCA**GGCGATGGCTAACACTGGGTATTG

CAGGGTGTTTATAGCAGCCTTTTAAAGCTCTGGACTCTGGCGAGGGATCA

AGGGAACAGGGTTGAAATCATACAGGTTTTACTGCTCTCTCCGGGGCAGT

TTTCTCACAACCACAGCTCTAGTTAGAACACGTTCTTTCAAGAAATGTGT

ATTTTATTTTAAGAAAAATGGGTTTGTTATACGGTAAAACTCTCCTAGGG

ATTCAATTGCCACTACTGTGCTTGTCAGGGGTTTGTGCTTTGCGCTCAAG

GGCTGCGGATTGTCTTTCTCTCCTGCTCGGTTTTCTTATTGTTGCTTAAT

TTTC

>dtnb1

chr12:3,616,341-3,618,9006341-3618900 5'pad=0 3'pad=0 strand=+ repeatMasking=none

AAGATTGAGTCCAGGGCCTCAGACATGGATTCACTTTATACTAAGCTGCA

TCCCCAGCCTGGGAAGAATTCTCTGGGCTCTGTGAATGTAACAGGGTGTT

TGCCTTTCAAAACCAACATGGTAATCTTTCACTGCAAAGTTGCTTTGCTC

TAAGTTACTGTGAAACTTTGTGATCACAGACTTTATATGATTACTGCAGG

GGCTGAGAGATGGCGCAGCAGTTAGAGCACTGACTGCCCCTCCAGAGGTC

CAGAGTTCAATTCCCAGCAACCATTTGGTAGCTCACAACCATCTGTAATG

GGATCTCATGTCCTCTTC**TGGTGT**GTTTAAAGATAGTTACATAAAATAAA

TCTTGTTTGTTTGTTTTGTTTGTTTGTTTTTTGAGACAGGGTTTCTCTGT

ATAGCCCTGGCTGTCCTGGAACTCACTCTGTAGATCAGGCTGGCCTCGAA

CTCAGAAATCTGCCTGCCTCTGCCTCCCGAGTGCTGGGATTAAAGGCGTG

TGCCACCACTCTTGGCTAAATAAATAAATCTTAAAAAAAAAAAAAAGATT

GCCGCAAAGTTCCATTGCTAGCCTGCACACGTGCTGACTTTAAGCAGCTA

GTGAAGTACTCTGTCTGCGGTGGCAGC**AGGTGTGA**CTGAAGGACTGACAC

TCTGGGGCTGCCCTGCTCTGCTGCAGGTGCACCGCATGCATCATGGCTGC

CTCTCATCTTTCCAGTGTTACCCTTCCACTCACAGCGGTGTACGGATGTG

AGACAGATGTGGTTGGGCTTCGCAGAATAAATACTTAACATCGAGTAATG

ACTGCTGCAAGAAAAAGAAAGATGAACATTCTATGAAAATTTTTTTTAAA

GAATTATTTATTTATTTTTGTATATAGTAGTACACTGTCGCTGTCTTCAG

ACATACCAGAAGTTGGCATCAGATCCCATTAAAGATGGTTGTGAGCCACC

ATGTAGTTGCTGGGAATTGAACTCAGGACCTTTGGAAGAGCTTTCTTACC

TGCTGAGCCACCTCTCTAGCCCCTCTATGAAATCTTCATAGCAGGGTGCA

CCTGGTATCTAATGTGTGAAGATAAACTGCTGTTATTCTGACGTTGGGGC

ATATTTTTAAATTGTATTCAAAGGATGAAATGCAGAGTTTATAAATGTGG

TTAAATTAAAGTATTTAAGAGTGAGAATCAGTTTGTCAATTGTTCAGTAT

TTAGAGATGCGTTTGGTTGCTTTTGTGGTTTCAGATAGCAATTATTTCTA

TTATTTATTTTGCAG

*TGAGGGCCGAGGCAAGTTGACCGTGTTTTCAGTTAAAGCTATGTTAGCAA*

*CCATGTGTGGTGGAAAAATGCTGGACAAATTGAGAT*

GTAAGTATTTTCATATTATTTGAAAGTTTCTGTTTTCAACTTAGGCTGT

TTAAACAATTTTAGAGTGAATGAATGTTTATAGGACTTTGGATGAGGCTA

CACTGTAGCAACTGACTCCATACATGAGCTGAGCCAGCTGCTGCTCAGAG

GCACAAGCAGACCGTGCTTCTTAGTGGTTTCTAAAGTGAGAAAACTGAAG

TTCAAAATAATGATATTGACATTTAAAAAATCCTTTATTTATTTAATTTT

TTTTTTTTTGGTTTTTCGAGACAGGGTTTCTCTGTGTAGCCCTGGCTGTC

CTGGAACTCACTCTGTAGACCAGGCTGGCCTCGAACTCAGAAATCCACCT

GTCTCTGCCTCCCAAGTGCTGGGATTAAAGGCGCGCGCCACCACGCCCGG

CATTAAAAAATCCTTTAAAATACCTTATATATTAGCAGAAATGTGATTGG

AGATGATTGTTTTTAGTTAAAAAGTAAGTTTAATTACTTTTATTGGGTTT

AGCCAAGTTTCGCCGTCTTACTAACTCCATTCTCCACCAGGTGATGTTTG

CTGGGGTTAAACTTTTCCTTAGTCAGAAACTGACAGCCCAATCTCCCTCT

CACCTGTCCTTTTTGTTGTTGTTGTTTCTGGTTGTTTTTTTTTTTTTTTT

TTTTTTTTTTTTTCTCTTTTATTGGGGGCTGCCATTTCTGCATTCACTAT

TCAGTGGTTAGTGTAAGAGGAAGACTAGAGCAAGTCGGAGAAGATGAAAT

TTATAACTATGTAATCTTGCTGTGTGTGGTCTTTACTTACCCAGCTATCA

GTTGAGGTAGCATTTCCCTCTACAGAGGGCGACAGTGGGGTAGTCATCTA

AATAGTTAGAGCCTAGGCCTTTTGTTTTGTTTTCTTGAGACAGAGTTTCT

GTGTGTAGCATTGTCTATCCTGGAATTCTCTTTGTAGACCAGGCTGACCT

TGAACTCAGAGATCCTGCAGGTCTCTGCCTCCCCAGTGCTGGGATTGAAG

AC**AGGTGT**CACCATGCCAGGCTTAGAACCTAGTTTTAAGCTACTTTTTTT

CTCCACTGTGCTAATACTGTCACTGCAGAGACACTCAGGGTCAGCTGCCA

CATTACAGAGAGCTGAAAGTGAAATGTTGGAATTATGTGTGCGGGGCCGG

GGCAGATGACTCGGGAGATTAAAGTACTTGTCAGAAGCCTAACAGGGCCC

CAGAACCCAC

>pus10

mm9_dna range=chr11:23606263-23608825 5'pad=0 3'pad=0 strand=+ repeatMasking=none

CCAGACTTCTTAAAGATTTAAACAAAATGTCTGTCCTGCTAGCAATTGTT

CAGGGACCTGGAATGTCTCTTCGATTTTTAGTTATCTCCTTCCAGCTCCT

GAATTTTTTTTTAAAAGATTTATTTATTTATTATATGTAAATACACTGTA

GCTGTCTTCAGACACTCTAGAAGAGGGAGTCAGATCTTGTTACAGATGGT

TGTGAGCCACCATATGGTTGCTGGGATTTGAATTCAGGACCTTCAGAAGA

GCAGTTGGTGCTCTTAACCACTGAGCCATCTCTTCAGCACCCCAGCTCCT

GAATTTTTATCCATCTTCATTCTAATGTACGCATTTGTCTGACACTGACA

CAAGCTGATTTGACTTAAAAACTTATTTGGGGGAGGGGTATTTGGGGAGG

TTTGGATGGAAAGAAGGGAAAACAGTTTTGCTAGTCTCATTGGTTACTGG

GTTTAGAAGTGCCTCCCTTCTAAAGCAGACAGGCAGGTTTGAGAGTTCTA

GTATTCTAGGTTCCTTGTAGCTTTCTGAGCCAACCTTTAGGAGTTGACTT

CTATTTCCATTCATAAATCAGTAGCTGTTAGTTTCTTTTTGCTCATTTAA

AACCCGTCTTTGTCATTTGTAG

*AGCTTGTTCGAAGTGAGCGTGGTCTTTG*

*CTCATCCAGAAACAGCTGAAGATTGCCATTTCCT*

GTAAGTTGCCCCGTAA

AAAAGTGTCAATTATGTAAAATTGTTAGAGTTTTTCTTTTTCATGAAGAT

AGTATTGTTTCTATTTTTTGTGACTACTTTAGAACTTGTCATGTCTTAGA

TTTAAAATGTGCCCCTTTGTAGAGATTCCTCAGAGAAACCAATACAACTC

AAAAATGTATACATTATATTTATATAGTATATAAAAATATATAAATATAG

GTATATAGGATGTATTTAATTATGTCCACCTAATATAATTTCATTAAATG

TTCTTCTTTTTCTCTTCCTCTGACTCTCCCCCTTCCTTTCTTCTTCTCTA

CCCCCTCTTCCTCCTCTCTTTTCTCTCCCCCCCTTTCTCTCTTTTTTTCC

CTCCTGCTCTTCTTCTTCCTCCTCCTTTTTTTCTTCTTTTTGAAACAGTG

TCTCACAATGTTGCTCTGGCTGTCCTGGAACTCACTATGTAGACCAGGCT

GGCCTCAAATTCAGAGTTACATTTGCCTTCTGCCTCACAAGTGCTGGGAT

GAAAGGCATA**TGTCACCA**CCACCTGGCTAGATTTTTATCTTAACTTGAAT

TTTATTTCAG

*AGGTGAAGTTTGCCGAGATTGTTTCAAACCAGCTAAAAAT*

*AAACAG*

GTACTTTTACTTTTTACCTTATGTATTTATTTGTTTTCTTCTGG

AATTTTTGAGACAGCCTTATGCAGGGTTACCTGGAA**CTCACCA**TATAAAC

CTGTTTGGTCTGGAACTTGTAGCTCAGGACCTCTTTGCAGAACTCCAATC

ATGCCATACATTACCCAGCCTCCGCTCTTCTCTGAACCACACAGCAGGGC

TCCATAACCCCCCCCAACACACACACACACACACACACACACACATACAC

ACACACACACAAATCTTGTGTCTTCGGTACCTAAAACACAGTGCCCTGTG

GATGTGCTGCTAAGTTCTGCTGCCAGCTCAGGATGTAGTCTGGCTCCTTT

GGCCCGCAGTTGTATCATCTTCTTTGTACTGACCCTGGGGAAACACTTCA

GTTGGCATTTACCTTCAAGGAGCCAAGAACCCCTTTGATTTAGGCTTACT

TCCTTCAAATGAATTTGACTCTTCACAAGATGGAACCTAAAGTGTGTGCG

GTTTGCCCTCAAGACACCTTTCCTGTTGCTCCAGTGCTGGGCAGGAGGTT

CTCTTCATCTCTTTAACAGTTGTCCCACGAGTAGCCCCTAGTCGTCCTCA

TGGAAGCTTTGTTCCACCTGACACCAAATGAACCAGGCCTGTACACATTC

CTGAATTCTCCTCAGCATCCTTATCTTTCCAGCTTCCACAAGAATGGCCC

AGTAGGCTCTTCTTAGATCATCAAGAGCTTCTCTCACTCAAGGTTCCCAA

CTCTTCCACATTCCTCCCGTAAGTCAGTTCCAGAGGCTTAAGGACCACAG

GGCCAGGCTCATCACACAGCAGTGGGCTGGGCTCAGTATTGATCCTACCT

TTGTTACTTTACCTTTGCTGTGTAAAATACCCTGATAGAATATTTGGAGA

GAAAAGTTTTGGCTCACAGTACTGTACAAAAAGGAGCCAGTGAGCTGAAC

ACAAGTATCCATTGAGTCTTTCTTCCTGATGTCTTGATCTGCCCTGGCTG

CTTTCGTTAAGGACATAGTCTGAAGAAGAGGCCTGGAGAGGCATGCCAGG

TACATTAAGAGCACCTAAGTTTGACTTGCTTTCTTGGGATTCTTAAGTAT

TTCTTGATTTTAAATATGTAAGATGATATAATTCATCCACTCAGTGTATT

TCAGCTCTTGTTTAGTTGATGTCTTTTTCATTTCACCCCATATAG

*TCGGTATTTACCAGAATGGCAGTTTTGAAAGCTTTGAGTAAGATAAAAGAA*

*GAAGACTTCCTCGG*GTA

>brca1

mm9_dna range=chr11:101389622-101392747 5'pad=0 3'pad=0 strand=+ repeatMasking=none

ACTCACATACTCTTGCAAGCATGCTCACAGACCATTGATAAAGAAGGGGT

TAAAAGACTCTTAAGAGGGCTGGAGAGATGGCTCAGTGGTTAAGAGCACC

GACTGCTCTTCTGAAGGCCCTGAGTTCAAATCCCAGC**AACCACA**TGGTGG

CTCACAATCATCTGTAATGATAACTGAATCTCTTCTGGGGTGTCTGAAGA

CAGCTACAGTGTACTTACATATAATAAATAAATAAACCTTAAAAAAAAGA

GG**TTCACACCA**CATTAAAAAAAAAAAAAAAAGATTCTAAGAACCGTAAGT

TGGAAAGACCAGAGCAAAACAGTGTCTATTGGACATGACAGAGGCACTGT

ACATGAGCTCATAGCAATTTTGGTTTACACAACATCTGTACATGATCAAG

CCTATCAACATTCTAGCAGAGAGAGGGGAGGGGCTCATGAGCCACACCCC

TAACTAAGGAGCTATGGACAACTGATAGATTCTGAGGGAGAGACAGTTTC

CTTCGAGGTTGTGGGGGCCTTTAGAGCAACAACCTATAAGCACACTCAGT

GGATGGTCCAACACCTTGGAGTAATGAAGAAATGGTTCAGGGTTAAGAAC

ATGGGTTAATTTCCCAGAGGACCTGGGTTTGATCCCAGCAAACACATGGT

GGCTCACAACCACCTCTAATGAGATCAGACCTCCGCTTCTTGTGCATCTG

AAGATAACTACAGTGTAATTATGTATAATAATAAACAAATTTTGGGCTGA

AGTGTGCAAGGACTGAGCAAGCGGGGCCGAGCAGAGCGAGCAGAGGTCCT

AAATTCAATTTCCAACAACCACATGAAAGGCTCACAACCATTTGAACAGC

TACAGTGTACTCATATACATAAACTAAACTAAATAAAAGATGTTTGGGTC

AGGCATAGTGGTGGATGCCTTTGGTGACTTGAAAAGCAGCAGACGATTTC

TGAGACTTAGAGGTCTACATAGTGAGTTCCTGGACAGCCAGGGCTCTATA

GACAGACCCCACCCCAAAATAAGTAATTATTACCATATATGAGAAACTCA

GAGAACAACGATCTGGCAGAAACAAGTGCATGCTGACTTCCACTGTTATC

TAAAACTCGTTTGGCATTAAAAAAGTATTCGGGGCTGGAGAGATGGCTCA

GTGGTTAAGAAGACTGACTGCTCTTCCAGAGGTCCTGAGTTCAATTCCCA

GCAACCACATGGTGGCTCATAACCATCTGTAATGGGATGTAC**TGGTGTGT**

CTGAAGACAGAGAAGTGTACTCATATACATAAAGTAAATAAATAATAAAA

AAACCCAACAAACAAACAAAAAAAGTATCCAACAAACAAACAAAAAAAGA

TTTCCCAGAAAATAAAGTAGATAAAACTAAATGGTCCTTAAGGTTTTTTC

TAATACTATGATCTAAATCTAGGCTCCTCAATACTAATTAGATATGACAA

TAGCTTCACAGACAAAGGCTCTCTTTGACTCAC

*CTGCA****ATCACCT****GGCTTAGTTACTGTCTCTTCAGAAGAATCAGAGTC*

TAGAAAAAAACAAAGAAAAT

AAAACCCAAAAATCAGTCAAGATTATTAAGGCTCAAACAAAACCAAAACA

AACAACCCAAAACAAACCAAAAAAACCCTGCTTTTTGTAAAACATATAAT

AGTTCATCCAGAAATATTTATTATATTTATAATTAATACCCACTATGTAG

CAGGTACAGACACATGTGGCAATACAGAGAAGTGGCCGAAAGCACAGGCC

CCAAAGCCAGCTTCCTATGTCCAAATTCTTAACTTCTTTGTAAGTTAATT

TTCTCATCACCTCAGGACTGACATTTTAGATAAGGTATAAAAAAACTACA

ATAGCACCTATATTCTAATCAAGTGCTCCTGAATATTAGCCGATAACGTT

TGCACTACTCCTAGTAATCCATAATTACTTGTATGTGCATGCATGTGCAG

TGTAGATGTCAGAGAACAGCTTGTTGGAGCTGATTTCCCTCCTTCCACCC

TGTAGATTGTAGGGATTGAATTT**AGGTGACAA**GTACCTCACCAACTAAGA

TATCTCCCCAGTCTCTGGGATATTTTTATAGATCTGCTGTTGATGGAGAC

AGATGGTAGTCTGGAACTCAAA**ACACCA**CCATTCTTCCCAATTTGATTAT

TTACTATTATGATGATGGTGATGGTGGTAGTGGTGGTGGCAATGATGGTG

ATGACGATGATTTTGGGCTAGGGTTCTCTGTGTATCCCTGGTTGACCTCA

AACTCACTGAGGATTCTCCTACTTTAGCCTCTTAGATGCTGGGATGACAA

ATGTGCTAAGATAGAGAAGCCTGTCTGAGCTCTCAGTCTTTTTGGCCAGC

TTGAGATTGAACACAGAGCTTGTAGAATTTATCCCAGATCCTTCTAAATT

CTATGGCAACCAAACATTTTTTTTTCTATTATGACTAAGTTTTTCCCAAA

ATTCACTTAAACCCATAACCAGACATTTTTCCTTTTGTTCCTTTTATAAA

GTAAGATTCTTCAAGATGTAAAGTTTTAACTTTTACAAAATCTATAATTC

ATGATAGAACCCATAAAATTATTTCATCTTCACTTACCAAAACTCCATGC

AACACATACACATCTATGAGAAAACTCTAAGCCAAACAATCTAGCAAGTA

TCATTTCCAACTTAATAAAACCTGAGCCCCTTAC

*CTAGTTCAATGTAGACAGATTTCTTTCGAGGTTGGGTCTGCCTGTTTTTC*

*TTCACTGATCTCACGATTCCAAGGTTAGACAGCTG****GACACCT****AGGCTGTC*

*CTTC*

TGTCAAACAGTA

AAGAATGCCTACGGTTTACTTTGTGAAATATTTTTTCAGATTTATTTTTC

CAAAACTAATTATAATCACTAGAAGGCCAGCAACATAAAATGTATTTTTG

AGAAGGATAAATGATAAGAGGAGGAATCTGAAGGGGAGGCTACAAAGGAG

GCACAGAGGTAGCAAGGGCGGGAACAGAGGGAAAGAACTATGAACAATCT

CACTTGAAAATGTCACAGTGCATTAGTATATGTAAGCACGTGGACTGCAT

TACATGCTGACTGAAAGCCATGAACCGCGTCAAGACAGCTTAGAGGCTAA

GAGCATGAATTGTTCCTGCAGAGGTT

>add3

mm9_dna range=chr19:53317258-53320391 5'pad=0 3'pad=0 strand=+ repeatMasking=none

AGACAGAGGAACCTGTTCGTCTCGGAATGTCATCAGCGCATGGGCTGTGC

TATCATACAGTGTATACACCAAGGCCGTAGGAATAGAGAATTTCCAGACA

CAAGAAATACAGGTCTTGCTTTGAACATGCATAGTATGTGGATTTTTAGT

CATGGATTCTCCAATACATGGTTTTAATTAATGCTTTATGAAATCTTTGT

TC**TGGTGT**CTCAAGTT conserved to dog GCCAGTATAGTCATTTCAAAACAACATTTTAAAT

CAACATTTAATGTAAACTGCTGGGAATTTGAAGTGACTTTCCTGACACAT

GGGTGCTCTAATGCAGTGGAGGTTTTCTGACTTTGCAAGTCTCTGTAAGT

TTATAAGTAGGGGAATCAAGAGCTGATGATGGAGAACCAACTGTGGAGGC

ATATGCCTGTGATCCCGGCACCCCAGGCTGAGCCAGGAGGATTTTTTGAA

GAGTAGCCTAGGCTAAGTGGGAGATCTTGTCAATAATAATAATAATAAAT

AACAATAATCATAATCATAATCATAGATTGTTAGGAAGAGGCATTAGGAC

TTGAGTCCTAGCTCTAGTTTGCTCCAGAATTAAGGATTGGCCTTAGCGGG

GCACTGGGGCTG**AGGTGTTAA**GCCACCTGAAACACAGTGAACTGTGTGTA

AAACTGTCTGGGCAAGAAGGATTCAAGGGAGACGATTAGAAGTCCCTGAT

CCAGTAGGAGCCTCTGCGTTGGCGCATCCGCATTTAGCTACAGCTAGCAT

TTCTACCCAAGGGAAACGGTCAGTAATGAAAGAATTACTGTGTCTGTGTT

AGATACCACGGAATTACCTGAGAGCTAAAGGCTAATGACAGCTGGGCCAT

CCAAGATACCACTTTTATTAGTGTCTTTTTATTTTTGTGTCTTTAGCACA

AAATCAAGTTACCCTACTCATTAATGCACTTACAGAAAATAGTTCTAAGT

GGCATATTTACCTTGTTTGTTCTAACATAAGATGGAAGAAAACATCTTTC

ATACTTTATAAAATGTCCTTATTCAATCTCTTACTTTTGAAAGCCTGACA

ATGCCAGGTAACAAATACCATACTCTGGATGTCACCTTTCGCATACACTT

TGTAAATAGTTCCTTTTACGTCTGTGCATAAGCTCACTAATACTGCTCTA

ACCGGAATCACTAACCGGAAATTGCTAGGACTCAGTGGAACCTCTGCCTC

GTTCCTGTAGCAAGGCTGCTGAGGCTTCGTCAGTCAGTTTTTTCATCTGG

CTGTATTTTGGTTTCCTATACTAGCAAAGGCTAAACGCATTCAGTCTCTG

TCTGGGCTGCCAATACCCATGCTTTTTTTAACTCAGTGTGCAAGTTACTG

TTCATGGCCAGAAGTTTAACACATCAACCTCCTCAGAATCCTAGTGGCTG

ACATGTGGGATGGCGGTAAGCATGCTCAGTTCTCAGCTGACGAGCTTGCA

AGCAGGCACTGGATGTACGCACTAACCTCTCTGAAACCGTAAGCTTCCTT

GTTTTGTTTGCATGGCTTTTAACTGAGCATCTTGTTCAACAG

*ACGCTGAGCAGGGGTCACTCTCAGACGACGCTGCATCTGTTTCACAAATT*

*CAGTCTCAAACTCAGTCACCG CAGAGTGTCCCTGAGAGATTAGAAGG*

TACTCACTGTGACTCCCACAGCCGCCCCCTCGTAAGCCCTGTCGTTTGTC

CCGTGGACGGG

ATCGGACACTTGCTCACAGTCCCTCTGTCAGTGCCCCACACATAGGGATG

GGGCAAATCTGACTCACTGCAATTTACATTCACTCGCTGTGAATTCACTC

GCCTGGCTTGGGTCAACCTATAGTTTTATGAATTTGTGGAATTAAAAATG

ACTTGCCCTCGTGTACCTTCTAATAAGTAGCATGAAGTTCTGTACAGTTT

TAAATGTCAAATGACCCTCAAATTAAAGAAAAACATATCTGTCGTCATCC

TAGCTTATAATGACTTGATTTGCCATGGAAAGGCGGACCACCAGTACAGT

TTGTGACCCTGTTTAACACTTGCCTTCTCCCCTTTGTATCATTGAAGAAA

ATTAGCTTCATGCTAATAGATACATGTTATTAAAATAATAATCCATCCTC

ATTAAAATTAAAGCAATGCATGGCTCAAGACATTGCTAAAACTTCTCAGG

TTTGCAAATGAAACAGAAACGTTGACTGAGTAAATAATCTCCCCCCCCCC

CACCCCCACCCCCAACCTCTCCTTTCGTAGAAAACCACGAGCTGTTTTCC

AAGAGCTTCACCTCCATGGACGCGCCTGTCATGATCATGAACGGCAAGGA

CGAGATGCATGATGTCGAAGACGAGCTTGCTCAGCGAGTCAGTAGGCTGA

CCACAAGCACGACCATAGAGAACATCGAGATCACCATTAAGTCCCCAGAA

AGAACTGAAGAGGTCCTGTCGCCTGATGGCTCACCTTCAAAGTCGCCCTC

CAAGAAAAAGAAGAAATTCCGCACGCCTTCTTTTCTCAAAAAGAACAAAA

AAAAGGAGAAAGTTGAGGCCTAAGTAGTCCTTTTGTAATTTGTATATTAC

ATTGTGGCATTGCACACTTAAATACCATATTTTAGTTAATCGTTAATATA

CTGTGGTAGATCAGATTGGGAGTATGTAGCAAACTGGACTTTAAAAAACT

GGAAAGAGGTTTTACTAAAAGAAAAATATTATTGAAAACTTTCAAATTCA

TCTCTTTTTATATGTCCGAAAACGGCCGAGCTTTTCAGCAGTGGCTGGCT

TGGCCGACTCTGCCGTCCCTGGCAGAGCGTTGTTACTGTTGCTCCTTAGC

AGCTGCTGCCTGGTGCCTAGGCCTCTGCAGCACATGTGCTGGGAAGGAGC

TGCCAGTGAGCACGAGACAATGCTTACTTGTCACTAATCAGACCTTGAGT

TTATCATCGTGGTTATTGACTGTATCTTTGGGTCCCCACTGTTTCCATGG

GCATTAATGGGATGTTTTAAAAGCTTCTACAAGACTCTAGAGCATTAGTA

TACATTGGCACATCATTTTTTAAAAAATGTTTTAAGAAAAGATTTGTCTG

GGTTTTTACTCATAGTATGAAATTCCCCTACCTGAAGTAACTGTTTGCCA

AAAAGTCATTTTAATAAACTATAATTTTTGAAGA

>fanca

mm9_dna range=chr8:125819099-125821402 5'pad=0 3'pad=0 strand=+ repeatMasking=none

CATAGAAAGGTAGACACACTATGATGAGCGCAGGCGAAAGGCTTCTCCCT

CTAAGCCACTCACATCCCAAGGATTTCCTCTCTGTTTGACTCATTTCATT

TTGTAAGATGGGAGTCTTATGTAGCCCCATTAGCCTCAAACGCACTAACG

TGACAAAGGCCTAAGATCCTGTTCTATGTCCCCATGTCTCAAGCACTGAG

GTTACAAGTGGGTGCCGCCAGGCCCAAACACTCATCATGTGGGCTTTGAT

TTGTTTTGTTTTATTGAGACAATTTCTCAATATCTTAGCACTGCTGGTTG

GCCTGAAACTCACAGAGATCCTCCTGCCTCTGCTTCTGAAGTGCTGGGAT

TAA**AGGTGTGT**GCCACCACATCTAGGCTTGATCCATGTTTCTAAGATATT

TTGTCTTCACTATTCTAAGATAATAGGGGTTGCGGTGGGGTCGCCATGGA

CTTTCTGCTTGG**AGGTGATAAG**CTGGGCTCAGGTCACCAACACTGACAGC

AATCACAATACAAGATGTTCAGGGTTCACAATGAATTCAATAAAGCTCCC

AGTTCCCCAGGCTGCGTCCACACTGGTCACCCCAGAAGCAAGTGAAACTC

TGTTGCTAACCAGTAAGTCAGTAATCCTTTATTCCCACATGGCTTCAATT

AGGCACCATCTATTGCCTGTTTCAAATATGGAGGCACCATGTGCTGTCGC

AGAAGTGAACAGGCTTGACGTGGAGGCACATGCCTTCAATCTTAGGACCG

AGGCAGGCAGTGAGTTTGAAGCCAGCCTGTTCTACATTGAGTTTCAGATC

AAAATCAGACATAGACCTGCCCTCATTCTATGCCACCTCAGACATCATCT

TTGACACAGCATCCACACCGGCCCGTCTCCCATGATAGAATATGACCCCT

CCATCCCCATGTGACCTTGAGAGCAAAAGGGGACCAACTTCAAGATGCCC

AGTAAAGTACTGCCGGCATGGCCATGTCTCAAACATGCTGCAGTGGGCAG

ATGCTCCCCTGCAGCTGAACATGCTGTAGTCGGAGGTGCTCCCCTGCATC

TGTATATGCTATAGTTGGC**AGGTGCT**CCCTGCAGCCTGTACCTAC

*CTGGCCTCCAGGACAGGCATTGGGATCTTCCCTGTTTGTTCAAACACCAT*

*GATGGCCTTCTTAACATCCTGCACCGCTTGGCTCTCACG*

CTAGGTCCATACAGGG

CGGGAGAGAGAAAGAAAGAAGATTTGAAAGCGCGTAAAGAAGCAGGCAAA

CCAGGCACCAACTTGGCTCCGGCTGCTCTGAGTGTTACACACCGCAGCGG

TAAAGGGTGAGATGGGCACCATTCAGCCTAGAACATTCCCAGATCCCCAG

GCCCTGCTGTGTGGACACACGAGCCCTAAGGGTCAGCTTAGCTCCAAAAG

CCAGTGCCATCTTCCAACTGCACTCGCTCAAGCCTCTGCTTCTCTCTCTC

TCTCTCTCTCTCTCTCTCTCTCTCTCTCTCTCTCTCTCTCTCTTCCTCTA

CCAAGACGAGAAAGAGGAAGGCTTTGAGGTGGGATGGGCTAGAATTGAGG

CTTAACTTTGAGATTTAAAAAAATTACTAAATGTTGGGGCTGGAGAGATG

GCTCAGCAGGTAAGAACACTGACTGCTCTTCCAGAGGTCCTGAGTTCAAA

TCCCAGCAATCTCACAGCCATCTGTAATGGGATCTGATGCCCTCTTCTGG

TGTGTCTGAGGGGAGTGATAGTGTTCTCATATAAATAAAAATAAATAAAC

CTGTAGAAAACATTGTTAAATGTCAAAGCTAGGTGTGGTGGCAAATACTC

CAAGTTTCCAGGAGACTGAGGAGTTTAAAGCCAGTCCGAGTCACATGATA

AGAAAATGGGGAAGACGGAAACAGAGAGAGGCAAAATGGCTCAGTGGGTC

ATGGTGCCTGCCATACAGGCCTGGGAGAAAACACAAAGCTGTCCTCTGAC

CACCACATGTGCACATGCATGGCCCATTCTTCTGACATATTTTATAAAAA

ATAAAAATGGGCCAAGCAAGCTGGTCCAGGAGGCAAAGCGTTTGCCACCA

AGCTGCTGTTCAACCCTCAGGATCTACGAGCTGAGAAGAGAGAACCAATT

CCTGTGGGTTCTGTTCTAACCTCTGCACCATAAAAAATCAGCTCAGGTTG

GTTGGTGGGGGCGCACGCCTTTAGTCCCAGCACTCTGAAGGCAGAGGCAG

GCAAATCTCTGAGTCTGAGGCCAGTCCAGTCTATAGATTGAGTGCCAGGT

CAGCCATGCTACACAAAGAGACCTCGTGTTGAAAGAACAACAACAACAAA

AAAG

>wdr70

mm9_dna range=chr15:7968428-7970905 5'pad=0 3'pad=0 strand=+ repeatMasking=none

TCTTTCCTACCAACCTACAAAATGTCAACTAAGTCATCTTCTTTCCCTCA

AACATGCTCTGTTGTCTTCGAATCTCAGGGTTTCAGGATCACTTTGGTCA

GGAAAGCACCTTCCCTTGTAGCACCCTCCTCTGTGTGATTTAAATCTCAA

CTAAAAGGAGACTTTCTTAGGGTCAAGCTGTAGTTCAGTGATAGACTAAC

TACATGTTTGCCAAGGCCTAGGCCTGCGTAACCTTAATAAGATAACAACA

ATGATAACTGCTTTATGTGTGGTAAATCCTTCTATGTTCTCTGTTTGTTT

TACATGTGTGTGTGCACAAGGAAACACACACACACACACACACACACGCC

AAGGTATCCCTGCAATCCTAGACCACCTTCAGGGGGCTACTCTCTCCCTT

CACTATGTGGGGTCCACGGATCAAGCTTGGCTGTTAGGCACCTGTACGCA

CTAAGCCAC**CTCACCA**GCCCTCTCAACCCTTCATTTCCTTTAGTTTAACT

TTATCCAACAATAA**TCACCA**CACATAAAACTACAGTGTTTATCTTTGGGT

ATTCCTGTCATTTCATCTTGCAGTATTCACTTCCAAGGGAACAAAGGGCC

CAGAAAGGTGGAGCCTCAGAACAGAGTGTAATGGTCC**ACACCT**CAGCTCA

GAACTAACTGCCATTCTAAGTCAAAGGCCAAGAAAAAGGCCAAATTGCAA

TTGATGTGAATTTATTACTGACAGACCTCTCTAGGATGTGATAAAGGATA

TGGATAATGGAGTCTGTGCTTCAAGGTTAGTGATCCCCAACAGAACTGTG

GCTGAAGAGCAGCCAGCTTGTACGGTGATACAGTCTCATTTCAGATGGAG

TCAGTCTTCACTGCAGAGATCCTAGTCACACTTGCAAAGCCATTTAGGTC

CTCTATTAGCATCAGTCTAATCCAGTGCGCCCCATGATAAACTCTACTGG

AATTTACAAAATCTCTCATTTCCATAGCATCCCTATTTGTTTAACTAAAC

TACACTGCAGACAGTAGTCAAAGCAAAGTCAAGAATAGGAACACATAAAC

ACCAAAATTCCTACTACTCTACTCCTTACTTTACTAGGGTTGCCATGATA

CAACTGCTAAAGAGAGCCTAGAATCCACTGAAGCATTCAAAAAAGTGCCA

ACAATACTCA

*CGCATCATTTGAGCAAGTCATAAATTCTCCTTTTATTTTG*

*GGATGCCATGAGCCAGTATGAAGCATTGCTGTGTGGCC*

CTTAAAGAAGAG

AGAGAAGAGGGGTTAAAATGTAAATACACTGACATCATACATTTAAGAGC

AAGAAGCCTTTGGGCACCCTGGAAATAGACTACCTTACTTATAAACTAGT

ATTATGTTGTACCAGCTTCATCCCATACACATGTTGGATTTCACTTGTTT

TCCATCTCTGACCAGTCTGGAAACCAGCAGTGCTTACCTGTCACGGGTGC

CTAATAAGGAGAGTAACTGGTATGTTCTCTTTAGGGAAAATAAGAGGCAA

AACCTAAGGCAGACAAGGAGCTTTTTTAAAGTCATGATCTTTTATGTAAT

AGAAAATAATCAAAGAAATTAGGAAGCATTTACAAACAAGCAAAGCAAG

**ACACCA**AGGTAGAAAATTCGTACATCCTAAAACTATGTTAACAATACTGAC

TCCACATCCTAGCTGTAACAATGAATATAAGAGGAAAAATATTTCAAACT

AGCAGGGAAATTATCTCTACCTAAAATAGAGAACAGGAAAAAAATACTAG

AATCAAAGAAACATGATAACAATGTAAAAATTAATATACAACCAAATCAA

TAAGAATACACATCAAATACAGATGCACATACAAACACCTCC**CACACCA**C

ACAATCCCAGGAGTGCAGTTAGGGGGAGTCTCTTACATATTGAGTCAGGA

GGATCGGGAGAGGCAGAAAAGAGGCTAACAGTTCTTATAGAATAATGACA

GGATCTCTAACAAAGTCCAAAATAAAGCTATACAAGAAAAATATCGCTCT

TTAAGCTCTCACCCTTTCTTACTCTCATCTCTAGCCCTCTTTCTCTCTCT

CTCTCCCTTCCCCCTTCTCTGCACGTGCCATGGCCAGCCTCTCTGTTTCT

ACCTTCTCTCCTAGCCCCTGCCTTTCTACAATAAAGTTCTAAAACCATTT

TATATATATAGCAGTGCCTCTCTGGAAAGAATAAATTTGAAATCAAAATC

TAAAAATCATTTCCAGCCTAAATTGACCACAATCCCTCAAATAGTATTTA

GTGTCATCCTAGCGATTCTATGGAACAAACAGGGACCACCAAGGTAGAGT

CTTGTAGAGTCAATAAGGAGACATGAACTAAAGCAATACCTATGGGGACT

AGATAAAAAGATGAGGATCTCTTCTGTATCTGAACTTGCGAGCATGTTTC

TAATTCCCCAATTGACATACCTACGTGCATTTCAAACACATATGTCCAAT

AAGGAAATTTTGATTTATAGTCTCTAAC

>cacnb3

mm9_dna range=chr15:98464367-98466844 5'pad=0 3'pad=0 strand=+ repeatMasking=none

AGAGATTGCGACTCTTTCCTCGAATGGCGGGGTGGGGGTGGAGGGCGCTG

ACTGGTGCTGGATGATTAGAACCAGCATCCTG**CACACC**GTACGTAATTCT

GGTCTTTTGCAAAGCGGTAGGCTTTACCCTTCTTCCTTCCCAACCTCAGC

GTCAGGATCGCACCTTTGCTCCACCCTTTAAGCTCACCTAAAACAGCCCC

TTTCTGCCCCAGTTCCCGCGGATGCTTTGGCAATGTCTGGCACAATCCTT

GGCAGGGAAGTGAAGAAGAGAATCTCCATCCCGGCTTAACTTGGATAAAA

TCTGCCCGTGCAAC**AGGTGTG** conserved to dog GGGAGGCCTAGCCACC

GTCCCCGAACTTCCCCCTTCTTAAGGGTCGGCCCTTTACCGCAGCCCGGAA

CGGAGAGGGCCG

GGCACCTGTTGGGGCAGGAGCTGCTTTCATGGCACGGGTCTGGGAGCTCA

AAACCATTTCTCCCTAGTACCACTGCGGGGTTTGCTTTGGGTGTCTCCAG

GACTTGCAGAGAGCTGACATTGCCGGATCACCTGAGACCTCTTCCCCATC

TGCTGTCCGTCCTCCTTTAGATCTCCATTTTCCTGTCTCCGCGTTCTCCC

TGCCTCCAGTCCTCCTCTCTCTCCAGCCATCCTCCCCGCCGCCCGCACCC

TCTCGATTCTCCACCGGGCTCGCGTCTGGTTGCGGCGTCTCTCCCCTTCC

CCTCCCCCGCGCCAGCCCTTTGTTTCCCGGCGGAGCAGTTCCTGGGTTGC

GAGAGTCCCTGTCTCTGCATCTCTCCTCGCCCCGCCTCCTCCCCGCCGGG

ATCCGCCCCGCCTGCAGCCTCTCCGCCCTTCTTCCCCGCCGCCCCGGCCC

CGGAGGTGCCGGGGACCCACCTTCCCGACTCGCGTGCGCCCGGTTCCGCG

GCCCCGGCGGGTCCCCCCTGGCTGCCGCGCCCCCGCCGCCCGCGTCTTCC

CGGGAGGGGGTCAGGTGGGCGGGCACTATTGTTGTGGGAGCCGGCGGCAG

ATTCCTCAGCCGCGCTGGCGCGCTCGGGGTGGGACCGGCTGGGCTGGGGG

GTGGGGAGGGGAGCGGTGATCTGAGCTGCGAGCAGCTGGTCTTCGCGGCT

CGCTCCTTCCTTCGCGCTCGCGCTCTCCGCCGCCGCCGCCCGCAGGGCTG

CGCGGCTCGGTGGCATCTCGGGCGCGGCCCGCCGTCCTCGCCCCCGGCGC

CGCTCGCTCCCCCCACCCACCCCGGACTCCCCC

*ATGTATGACGACTCCTACGTGCCCGGGTTTGAGGACTCGGAGGCG*

GTGAGTGCCCGCGGGGGAGGGTGGGGGGTGGGGGTGGGGGAGTTGGGA

**TGACACCT** conserved to horse CTCTCTCTTCAACCCTTCCCCCAGTTCTGCACTTTGAAAAACGCC

TTGGCAGCGAGCGGGGCAGCGCGCGTAAGCCGGCGGGGGGCTCTCTAGGAAAGTGGGAAGATGGTCTCTCATAGCCAGGTATCCCAGGTACTCC**GGTGTGTG** conserved to elephant CACAGGGACGCTGTAGGGGGAAGGCTCGAGCAGGTGGC CCCAAAGCTCAGGACATAGGAGGCTGAGGAG

GGGAGGACAGAAGGCCCCGACACCGAGGTTTTTAGAGCGAACATGGAACA

TTTGGAGTTCCGTATCCTGGGTTCATGGGGTCTGAACACAACAGGGAAAG

CTGAGCTCCGGGGGAGACACTCCGGACTTGTGCCTCAAGTAAATGACAAA

TTAGGGAGCTGGAAGAACATTAGCGACTCGCGTACCAGCCTCTGCAGACA

GGAATTCTAGAACTTGGTCTCTTTTTCCTCTCTGCCCACCCCCCCTGCGC

ATGGCCTTGACTGCTCTCACTCACCACTGCCTCTAGCTCAGCTTCTTGGA

AAGGCCACCAACCACAGGGGTGGGCCATCAGACTGTGTTCAGTCTAGCCC

CCTGCAGAGGAGGGCCTGGAGGTGAGGCCAGGGGAGCTCAGGGGCATGGG

GATGGGAAGGAAGCTCTTTGCTCCGAGAACCAGGCAACCAATAAATATTT

AATTCTTCTCTTTGCTTTTATCTCCTGACCCACTGAGACAAATCAGCTCA

TTAGACAGTGATTCCCGGAGCTCAGAGAGCAGAAAGACTGCCTGGAAAGA

TGGGACAAGAGTTGGGGGGGGGGGTCAGTGTCAGAGCCTCATGGAGTCAC

CAGTCTCCCCTTAGACATCCCACTCGATATGGGAAAGGACCACTAACAGG

GCTTGAAAGGGTCCCCTGACAGGATTAAATCGAGCCTTTTATCTCCGGCC

CAGGAAAGAGGAGTGGTGAGCCTCAGAGCAGGCATGTGTCAGTCAGTTGA

TTCTGGGACCAGGCTCCTTCAGGACCCAATTTTCCATGGGGCTCTGGGGC

TAATTACGTTGCAAGGGAGAGCAGAGAGGTGGTTCTGAAGGCTCTGGTGG

GCACCTCTTTCTGACAGTTGAAGGGACAGTTGGCTAGCCCTGGGAGTCAG

AATAAGCTGGAGGAGAGACCTCTCTGC

>arnt2

mm9_dna range=chr7:91413063-91415244 5'pad=0 3'pad=0 strand=+ repeatMasking=none

CCATGCCTATTGCCAAAGTCATGGACTTTACTCTGGTGCTATCATATATA

CCCATTACTTTGATCATATTACCTCTGTGTCTCCCTTTATTTCAGAGGAC

ATTTTCTAGACATTGTTCTTCGTAGTTCTTCTTTAACACATGCCTTAGCC

TGCACAGTTCTCCTTAGGATGCTGACTAATGAGGGACAGCTGTAACTCTG

CCTAGGATTTTTGGAACAAAGTGCTGGTCCTTATGCCCAGCCCTCCCTAG

GGCTGCTGG**TCACACCT**GCTGCTGATGCAGGAAATATGTTCTTAAAACAT

CAAGGAAGGGAGATTTCTATCCCATTGTGGCCAATGTAGTTTCTGGGCAC

TGGGTGTGCCTGCCTAACCCTGTTCAGGGTCTCTGAAGTCCTGTTTAGAG

TTGGGCCAAGCTCAACTGGGATGTAAGAAAGACATTGCCTTGCTGTACCT

AGGGTATGTGAAGCTTCTAGGGAGCTCAGGGGTAACTGTTGTGACACAGG

AGGATGCCTTCTCTGAGGACTGCTGGCCTGTCCTTTGGCAGTCCTCCTGG

GTTCTTGCTTTCATTTCTCAGAAGAAACTTGAACCAGGTTTGGCCCTCAG

TGGTAATGTCAAGCTTCTTCAGTAAGCTATAGCATGCCTTGATGCTTTAT

AGCCAGGCACCCTCCTGTACCCTCTAATAGTTCTTAGATATGACAGACCA

CCTTTTTTCCTTGCCCTTGGCCATGGCCTCAGCAAATTAGAGAACAGGAC

AGTTCTAGCTACTTTCCCAACAGGGCTCTACCTGCATCTTGGCTATAGAC

TATCTCCTTCCCTCTTTCCCTGCAGAATCACAGAGAGGACCAGTATCTTC

TGTGTCTTAGAGTCACTGACACTCAGTGCCCATGGAGGGGCTGATGGAGC

AGAGCTAGCAGTCCAACGTCTCCATCCTGGCTCCATCACACAGTACAGGA

TTCCTGCTCAGTCACACAGCCAATGCTGGCACTCAGGCCTTTCTTTCTAC

CAATCCCCTCCC**TGACACCT** conserved to dog AC

*CTGAAGGCCTTGCCCGAGTGCCCGGCAGGGAATGGACTTCCTTGGGAGTAG*

*ATCTGCTGGCTGCCTGATGCTGAGGCTGAGCTCATCATCTTCTTCTCAG*

CTAGGGAAACAATAAGAGTTGCATGTGA

AGGTCCCCCCTTGTGCTGCAGGCCACCAGGGAACACCCTGGAACAGTGCA

TGGCCTGGGAACTCAGGAGATCCATGTAGGAGAGCCTCCTCATCTTCAAC

TTCTCTTGGGCTCTTGGCTCCTAGGATGGCCCCAGCCAACTGGAGGCAAG

CTCTCGAGAATGGGGAAGGCACTATCGTATTTAGTCCTTCAGTTTCATAG

GGGAG**CACACCA**TAGGAAATGTGTAAAGAGGCGAGGACACCCAGGGTAAC

CTCAGCTGCCTGGTCTTGGACCTTGGCTTGGTCTTTTGTGTCACAGAGGT

CACTTGGGATGTTCCTTGAACTCTCCCCTGCCTCAGTTTCTCTACCTGAA

AGATGATAATTGTAGTCTCTTTCTCACTGGGCTGTGGTGAGGATCCTGTG

TTAATATAAGTAAAATGTTTACCGAGGACAGTGCTGGGAAAAACCAAACA

GCCTAAACATTTGCTTTCATTTATTGCAGATGAGAAAACGGAGGCACTGC

TGGTCCCAGGTCACAGTACAGCAAGGGAGGAACCAACTTGAATCCCATCC

AGTGGCCCTTCCCAGACCTGATCCAGCAGGCACTGAGGTCTCACACAAGA

AGGAAGCTGCTTTTGGTGGTGATATGAGAAGCAGGAACCAGATGTGAATA

GATGCAGGCTCCAGGCAGCAGAGCGTCAACCAGAGTGCACCCCTCCCTTG

GCTCCATGATGCATCATCATCTGTGACCCTAGAATATCATGATGCCTACA

TGTCTCATTTATAAAATCACCCTCATGACAGTGGGGGGGAAGGAAAGCAA

GGCTGGATACATGACAGTGACTATGTAGCATGTATGCCTCCCTGAGCCCA

GCTTGCCAGGCGGGTGTGAGCAGTACTGGGCTCTGATGATCAAGCCCACA

GGCCCGGGTCTGGGAGGCAACACAGGAACTAAATTCCTACTCCCATCTCT

TGGGCTCTAAATAAAGTCATTGCCATATTGCAGCTGTGGGGCATCCTAAA

TGGACTGGAGCTCCATCCCTAGTCTAATTTCA

>Nfkb1

>mm10_dna range=chr3:135612651-135614742 5'pad=0 3'pad=0 strand=+ repeatMasking=none

(flanking exon 11)

ACTTAAATAAAGTGTGTGCAAACCCAGCACCTGGAGAAAGTTCCCAGTTAGTACCTTATACCCACACTTATACTGCCCCCGTGAAAGTAAAACACAACAGCATGATGGCGGATCACGCGACTCAGCCATTTCTCTTTCAGATTAGCCTTGAAAACATCCAGGATCACTGCTAGCAGCAAGAGAAGGAGTGGATGTGGAAGCAGACAGAACCAGGGCTGCCGCTGACAGCCCGCAGACAGTCATGCTAAGTGAGGCTGCGCAGG**AGGTGTCA**CATGCTAGTCCTGGGCAGTGCTGGGATAATAAGGAACTGGACAGACTTAGTTTCGGTCTTTCTATGTTTATAAGGATCAAGATGTCATCAAATATAGGATGAGCAATAGGTAGCATATGCTGACAGTTCTCCCTGCCTCCCCGCTTCCCACTCATGCACTAACTTGAGATCCTAGAGGGAGCTGATGTTGTTATTGTCTCAGGCTTCCTGTGCACGTGAACACAGAATTGTTTCTACATAGAATTGTTTCTACATACAGTGTGTTCCATATCGATTGTTACAAATCTTTGCATTCTATTTTGAGGTCTTTGTTCATTCACAGAGCCTTCTTTTCTAGTAACTGTATGATGTTCCATTACATAGATACACCACTATCTAATTAGTCATTTACTGGCTAACATTTGGGTTGTTGCCAATTATTTGAAACACCATTTCTCAGTCACTGTCAAAGAGTGACTATTACTGTGGTGGCTCATGTCATCAGCTGGGAGACCCTGGTCATGTAACCAATCAGCCAGAG**ACACCA**GCTCATCAGGACAGCATCAGCTTCAGCCCTTTAGACTTCTCAGCCTCTATGTCAGAGTACTGGAAGAGCCATAAACATAGCATTGCTGTATGGATGTCAAAGAGACTTATGACGGTTTCTTTGTTAATATGTGGAAACTGAGCACTGAATTTCACCAAGGCAGAAGTGCATTAAAATAAAACCCGCAATAATAACCCACCTAC

*CTGTCTATGAACATCTGTGGGGGAAAAGTCCCCAAATCCTTCCCAAACTCCGCCATTTTCTTCCTCTTCATAAAACCGAATCTGGATGTCATC*

TGCAAACGGGCGTATTCAGGCATGTGATTGCAATGCGTGTAA**TGGTGTT**GAAGAGCGCTGTCTTTTTGAGACTCCCACAGCAAAAGGAGAACACCCCTGGTCCGCTGTTTCCAGAGAACTCTGACAGCCACACCCTACATCTAGAGACC**AGGTGTG**TACGTACCTTTCTGAACCTTGTCACAGAGAAGGTAAATCTCCTCCCCTCCCGTCACACATCCTGCTGTTCTGTCCATTCTCACGATTTTCAGGTTGGATGCATTCGGGGCTTCTGTTCAAAGGGAGAAGAGAATGCATACCCTGGCTTAGCATTAGCCTCACAAAGATTCCATGATGATGCTCACTCTTACTGCCTTTAACAAGACACAAATAGAGCAAGAAAGACTTCCAAGGTACTAGGAGCAGTTCTTGGGTCTGGGGCACTCATGGTTCCCCAGAAAGGAGCAGGTCAGGGAGGGATGGTGGCATATGGCTTCTCTAACACACAGGCTGATGAGGAAGGAGAGAGCTGTCTGATGTTTCTGGACCAATGGCTTGTTTCATGCTAACTTTATGTCATTTGTAAAATTCTTAAAAATTTTAAGAAAATGACATTTTAAGCTAATATATATATATATATATATATACACATTTACACACACACACACACACACACACACACACACACACACATTCTTCAAAGAAAAGTGAAGATGTTCTGGTTTTTTTCCCCCAACCTAAATTAGCATTCTGTGTGGTTTGGGGCAAAGCTGCCCATAG**TGGTGAGT**GGGGCAAAGCTTCCCCCTTTAAAAACTCCAACAGCTGTAGCTCATCTCTGTTCAGTAAGACTCTTTTCCATTCAATAAACTTTTCACTTCCCTTCACACTTGTTTAAATCAGACAGACCCATGCGTGGCTGGATGCAGAACATGGGATTTCCTTGTGCATCGTATTCTATGACCATTCAGTGAGGCTTGCAGGGTTCCTGAAACAACTGTTTGTTAAGCCCAATGCATATCGAAC

>Nfkb1

>mm10_dna range=chr3:135658028-135660065 5'pad=0 3'pad=0 strand=+ repeatMasking=none

(flanking exon 5)

AGCTTTACAAAGTAGCATGGACATCCCTAAGATGTTTCTCCTATTTAACAGCTGACCACATCAGAATTCTACAGCACAGCACCGAATGCTGTCAGCATTCACTTCTTAGCATTGAGTGTCATACAACACAATGTGTTAACCCACTCTCCTGAGTTACAAGAAGGAACAGCCTAGAAAAGACATTGGGTGACACAACTCGGTAGGCCAGGTATACAGTCCTTCAGCGCCACGGCTGTGATGAGCATATCCACCCAGAGATCTAGTGACTTGGTTCTCACTCTGTGGGAGGCTATACCATAACTTCCATCCCATAATGACTGAAATGTTCTGTCGGCAGATATTGAAGATTCTCTTCTCTCTTACAGTACTAGAGCCATGCAACTTCTAATGAGCATATTTGCAGTTTTGAGGCTATTTTAATCTTCAGCTGGTGAACACAATATTTAGTAGAAAGCGAAAGAGCCAGACAAAAACACAAACAAAAACAACAACAACAACCAAACAAAAAAATCCTCTCCTTGCTTCAAAAAGGCTGGGATTAAAGCCAGAGGGGTGGAGGGGAGATCTCCCAGTCCACTCTAATGTCTCCCAGTGCGTTTACTGCCTTAAGCTACACATTCTATGCACAGAAAAGGCCAGAGCTTATGAAGGCAGGAGGGGTACCCTGTGGGCATGGCCATGAGCAATAGAGCGTCAGAGTGGCTGTGAACCTGTGTGTGTGTGAGTATTTCTTCATGAATGAGACTCTTTAGTGCTTTCCCTGGAAGCAGCTGATGAGCTAAGCTGGAAAAAGGACAGACTACCAAGGATTTATAATTAACTGATGTTTCAAAGCCATTTGTAAGAAAAATGTAGACACAGGAAGTTATTGCATAACACGGCAGGCTGCCGATTATTCAGATAAAACACTTACCTCTGTCATCAGATTTAACTATTACTCAGAGACCGAGCCTTACCATGAGATGTAGCATTACCAAACACCCGACCCCTTTACGCTTAC

*CTGTTTTGGTTGCTCTAATATTTGAAGGTATGGGCCAT*

CTGTTAAAAACAAGTACAACACAAGAAAACCAAAAGAAAACTGTGAGACTTCTGGAATCTTGTATGAAATACAAAGCAAGCCAAATACTGAAGTTATGCTTTAAACAGAGATTTCAAATCAATTTTTATAGGTGAAGTGTGTTCTACTGATATGTGTTCTCATTTGAAGTATTAAATAAATATTTTTAAAAATATTTATTTTATTTTTTGCTCATAAGTGCTTTGCTTAGTGCCCAAGGAAGCCAGATGAGAGTGTCTGATGCCCTGGAACTGGAGTTACAGGTGGTTGTTAGCTGCCATGTGATACTAGGAACCAAGCTTGGGTCCCCAGGAAGAGAACTGGACACTCTAAACTGCTGACCAATTGCTCCTGCTCCTAAGTTCAGCTTATTTTAACAGTAGTTTTATAGTTTAAGTGACTAACGGTAAATAAGTGGCTTTTTCTGAATGGTAATGGT**ACACCA**CTGGACTGAACAAAAGGTCTGAATGCTGACAGAGATTCTGATCCGAGCCGTCCTCAGACTCATGTGTCAACAAACATTTAGGGCACAATACAGCTGAGTGCAGGGAGCACCTCTGAGTGCTG**TGGTGTCA**GGTGGCCTTCACCTGCAGACTACTTTCCTCCCTCAAAGAGAACTGCCCAGGAGTAAATGAAGAACTGAAGCTAGAAACCCACAGAAGGGAGATCCTGGGCCACCTGCCTCTCCTAACACTGCTCCTGAAATGTAGTCTTTCCCTTACTTCAAGCCAAGGGACAGCCACTGTTGCCTAACTGGCTTCTTCCTTGCCCTCTGTGAGACCTGAAGAACTTTTCTTGCTTCTCCATGGCTTGTTTTGTTTTGTTAGACAGAGA**CTCACCA**TGTATCCTTGGCTCGCCTGGAGCTCACTATGTAGACAAAGTTGGCCTCAAACTCACAGAGATTTTCTTGCCAAGTGCTGGGATTAAAAGCATGGACCACTACCCTGGCATCTTACTCACAGCTTTTGATGCAGATATAAT
