# Supplementary material for: TBX3 Regulates Splicing In Vivo: A Novel Molecular Mechanism for Ulnar-Mammary Syndrome
Source: PLoS Genet. 2014 Mar 27;10(3):e1004247. doi: 10.1371/journal.pgen.1004247 (PMC3967948; doi:10.1371/journal.pgen.1004247)
Supplement: Text S1 — Supplemental Methods and Figure Legends. This supplement contains 2 sections: Supplemental Experimental Procedures and Supplemental Information References. (DOCX) [file pgen.1004247.s010.docx]

**SUPPLEMENTAL INFORMATION

Supplemental Experimental Procedures**

Antibodies

We synthesized a KLH conjugated peptide unique to the C-terminus of Tbx3/TBX3: GLEAKPDRSCSGSP. Rabbits were immunized by Covance. Polyclonal antibody was purified by peptide immunoaffinity chromatography and validated by immunoblot, immunoprecipitation and immunohistochemistry using protein lysates from wild type and *Tbx3* null mouse tissues [[1](#_ENREF_1),[2](#_ENREF_2)]. Additional antibodies: Tbx3 (SC-17871, goat polyclonal against internal epitope at 5’ end), hnRNP C1/C2 (SC-32308), DDX17 (SC-130650), DDX3 (SC-365768), FMRP1 (Cell Signaling 4317S), hnRNP U (Novus Biologicals-100-2135), hnRNP K (SC-53620) and PABP1 (SC-166027).

Protein extraction and immunoprecipitation

Cells were washed in PBS and lysed in 5 volumes of Dignam buffer (25mM HEPES ph7.9, 25% glycerol, 0.42M NaCl, 1.5mM MgCl2, 0.2mM EDTA, 0.5 mM DTT with Protease inhibitor (Complete EDTA free, Roche). Lysis and subsequent reactions were performed at 4°C in a cold room. Lysates were cleared by centrifugation at 12,000g for 15 min. and snap frozen in liquid nitrogen and stored in -80°C freezer. For each immunoprecipitation, cell lysates were prepared from 107 cells with or without RNase A (Sigma, R4642) were then incubated for 4 h at 4°C with the appropriate antibody followed by 2 h at 4°C with the pre equilibrated Dynabeads Protein G (Invitrogen). Immune complexes were collected and washed three times with lysis buffer. Pelleted beads were resuspended in 6X Laemmli buffer and subjected to SDS-PAGE analysis followed by immunoblotting with specific antibodies.

For immunoprecipitation of endogenous proteins from mouse embryos, embryonic day 10.5 embryos were dissected and washed twice with cold PBS and snap frozen in liquid nitrogen. Embryo lysates were prepared by resuspending the embryo tissue in 5 volumes of Dignam buffer followed by homogenization in a Dounce homogenizer. Cleared lysate was obtained by centrifugation at 12000g at 4°C for 15 min. Supernatants were stored at -80°C . For each IP, 1 mg of cleared lysate was used.

Immunoblotting

Whole-cell lysates and immunoprecipitates were separated by 10% SDS–PAGE (or) 4-12% NuPAGE precast gels (Invitrogen). SDS-PAGE resolved proteins were transferred on to PVDF membrane by Trans-blot semidry transfer cell (BioRad) as per the manufacturer protocol. Specific proteins were revealed using ECL Plus western Blotting detection system (GE Healthcare).

Dual -tagged pull down and immunoprecipitation assays

HEK293 cells were transfected with Tbx3-Myc-His dually tag plasmid by standard transfection procedure. Cells were washed in PBS and lysed in 5 volumes of lysis buffer (25mM HEPES ph7.9, 10% glycerol, 200mM NaCl, 1.5mM MgCl2 with Protease inhibitor (Complete EDTA free, Roche). Lysis and subsequent reactions were performed at 4°C in a cold room. Lysates were cleared by centrifugation at 12,000g for 15 min. and then incubated for 4 hr at 4°C with pre-equilibrated Ni-NTA agarose beads to bind the His tag on TBX3. Bound protein complexes were eluted with 2 volumes of elution buffer (25mM HEPES ph7.9, 10% glycerol, 500mM NaCl, 250mM Imidazole). Eluted samples were dialyzed in Dignam buffer at 4°C overnight. Dialyzed samples were analyzed by western to detect His-TBX3 associated proteins (lanes labeled Ni-NTA in Supplemental Figure 1F-J), and also subjected to second round immunoprecipitation using anti-Myc antibody as described above. Immune complexes were collected and washed three times with lysis buffer. Pelleted beads were resuspended in 6X Laemmli buffer and subjected to SDS-PAGE analysis followed by immunoblotting with specific antibodies for the proteins of interest (lanes labeled anti-Myc in Supplemental Figure 1F-J).

Enzymatic digestion of protein from cultured cells and mouse embryos.

Proteins were separated by SDS-PAGE and in-gel digested prior to analysis by mass spectrometry as previously described [[3](#_ENREF_3),[4](#_ENREF_4)]. Gel plugs were dehydrated in acetonitrile (ACN) and dried completely in a Speedvac. Samples were reduced and alkylated with 10 mM dithiotreitol and 10mM tris(2-carboxyethyl)phosphine solution in 50 mM NH4HCO3 (30 min. at 56°C) and 100 mM iodoacetamide (25 min. in the dark), respectively. Gel plugs were then washed with 50 mM NH4HCO3, dehydrated with ACN, and dried in a Speedvac. Gel pieces were then swollen in digestion buffer containing 50mM NH4HCO3 and 20 ng/μL of trypsin (Promega, V511A), and incubated at 37°C overnight. Peptides were extracted with 0.1%TFA in 50% ACN solution and the volume was reduced and ACN removed in a Speedvac. Peptides were then analyzed on a mass spectrometer.

Mass spectrometry analyses and database searching.

Ten microliters of extracted peptides were analyzed by nano-flow LC/MS/MS on a Thermo Orbitrap with dedicated Eksigent nanopump using a reversed phase column (75 µm i.d. 10 cm, BioBasic C18 5μm particle size, New Objective). The flow rate was 200 nL/min for separation using a mobile phase A (0.1% formic acid, 2% ACN in water) and mobile phase B (0.1% formic acid, 20% water in ACN). The gradient used for analyses was linear from 5% B to 50% B over 60 min, then to 95% B over 15 min, and finally keeping constant 95% B for 10 min. Spectra were acquired in data-dependent mode with dynamic exclusion where the instrument selects the top six most abundant ions in the parent spectra for fragmentation. Data were searched against the mouse (version 2011.03) and human (version 2011.03) Uniprot databases using the SEQUEST algorithm in the BioWorks software program version 3.3.1 SP1. All spectra used for identification had deltaCN>0.1, consensus score ≥ 20 and met the following Xcorr criteria: >3 (+2), >4 (+3), and >5 (+4). Searches required full cleavage with the enzyme, ≤ 3 missed cleavages and were performed with the differential modifications of carbamidomethylation on cysteine and methionine oxidation. Mass tolerance was 2 Da for precursor and 0.5 Da for product ions. All 75 interacting proteins reported were identified in at least two independent immunoprecipitation experiments and on the basis of two or more unique peptides. Proteins included in Supplemental Table 1 with lower identification criteria were manually validated to confirm identification by mass spectrometry.

Bioinformatic analyses of MS datasets

Gene Ontology (GO) analysis, was performed on the 75 proteins identified from Tbx3 immunoprecipitation experiments to determine the enrichment of annotated molecular functions (MF), biological processes (BP) and cellular components (CC) using the DAVID Bioinformatics Resource (v6.7) developed by the NIAID [[5](#_ENREF_5),[6](#_ENREF_6)]. The Interpro, Prosite and KEGG (Kyoto Encyclopedia of Genes and Genomes) analysis functions of DAVID were utilized to determine enrichment in protein domains and functional pathways.

Purification of MBP and MBP-TBX3: Amylose bound MBP and MBP-tagged TBX3 affinity columns were prepared as per the in the manufacturer’s protocol (E8022S, NEB). Bound proteins were eluted with 3 volumes of elution buffer (20mM TrisHCl, 7.4, 200mM NaCl, 1mM EDTA, 10mM Maltose) analyzed by SDS-PAGE and coomassie brilliant blue stain, and employed for GST-MBP pull-down assays.

GST-MBP pull-down assays: Expression of GST and GST-DDX3 proteins in BL-21 DE3 cells was induced by treatment with 1mm IPTG for 4hrs at 370C. Bacterial cells were pelleted by centrifugation at 12,000rpm for 10 min. Cell pellet was resuspended in 5 volumes of lysis buffer (50mM Tris HCl, 7.4, 150mM NaCl, 0.05% triton X 100) and cleared lysate was obtained by sonication followed by centrifugation. Equilibrated glutathione beads were incubated with the bacterial lysates at 40C for 4 hrs. GST and GST-DDX3 bound beads were washed 5 times with wash buffer (50mM Tris HCl, 8.0). Small aliquots of bound beads were initially analyzed by SDS-PAGE and coomassie brilliant blue stain. GST, GST-DDX3 bound columns were incubated with purified MBP and MBP-TBX3 proteins at 40C for over night. Beads were washed 3 times with wash buffer (20mM Tris HCl, 7.4, 200mM NaCl, 1mM EDTA) and bound proteins were analyzed by western blotting with anti-TBX3 antibody.

Plasmids, cell transfection and reporter assays

Wild type Tbx3 and Exon 7 missense, Tbx3 deleted repressor domain (Tbx3 Δ RD1), Tbx3 Δ NLS, were generated by PCR amplification and then cloned into the pcDNA3.1 vector. The missense mutation in exon 7 is an insertion of a 43bp polylinker in the Not1 site 1961bp 3’ to the ATG; in addition to adding 12 amino acids, the frameshift caused by the insertion results in a transcript of 2786 bps and a protein 762 amino acids long that terminates at a frameshifted stop codon in the 3’UTR of the wild type gene (sequence provided upon request). The C-terminal deletion constructs Tbx3 1-656, Tbx3 1-623, Tbx3 1-565, Tbx3 1-471 were generated by PCR amplification and then cloned into pCS2 with an N-terminal Myc tag. Tbx3 L143P and N277D point mutants were kind gifts of Phil Barnett. The *in vitro* splicing “T-box vector” mini gene construct was generated by cloning the T-box consensus DNA binding element (AATTTCACACCTAGGTGTGAAA) into pRHCglo [[7](#_ENREF_7)]. Sequence of all plasmids was confirmed. Transfections were performed with Lipofectamine 2000 (Invitrogen) as per the manufacturer’s recommended protocol.

RNA isolation and reverse transcription–PCR analysis

Total RNA was prepared using the RNeasy RNA isolation kit (Qiagen). For RT–PCR analysis, total RNA was reverse transcribed with oligo(dt)12-18 primers using the SuperScript III reverse Transcriptase (Invitrogen). cDNAs were PCR amplified using Taq DNA polymerase (Denville Scientific) with the gene specific primer sets as noted in the figures.

pRHCglo minigene FP: CATTCACCACATTGGTGTGC

pRHCglo minigene RP: GGGCTTTGCAGCAACAGTAAC

mBrca1 FP1: CATCTCCAGCTTCTTGAGGGGCGGTCTGTAAC

mBrca1 RP1: CCAACCTCGAAAGAAATCTGTCTACATTGAAC

mPus10 FP: CAGCTGAAAGAAGCCTACAAGTGGATAAC

mPus10 RP: GCTTTCAAAACTGCCATTCTGGTAAATACCGA

mNfkb1 FP: TCCAGGTCTGATTTCCTCCGAAGCTGAACAAAC

mNfkb1 RP: AGAATGGACAGAACAGCAGGATGTGTGACGGG

mNfkb1 FP2: CTCAATATTTAATGCAGAATTATATTCACC

mNfkb1 RP2: GCCCCTAATACACGCCTCTGTCATCCGTGC

mDlg3FP: CGAAACGTAAAAAGAGTTTTCGCCTCTCTCG

mDlg3RP: CCCAAGATGATCACAGGCCTGGCATAGTG

mp21 FP: TCCACAGCGATATCCAGACA

mp21RP: GGCACACTTTGCTCCTGTG

mCcne FP: CTGAGAGATGAGCACTTTCTGC

mCcne RP: TGGAGCTTATAGACTTCGCACA

mGAPDH FP: GAGTCCACTGGCGTCTTC

mGAPDH RP: GGGGTGCTAAGCAGTTGGT

mH2a FP: CTCGCGCCAAGGCCAAGACCCGCTCCTCCC

mH2a RP: TGGATGTTGGGCAGGACGCCGCCCTGCGCG

hNFKB FP1: TCCAAGTCAGATTTCCTCCGAAGCTGGACA

hNFKB RP1: AGAATGGACAGGACAGCTGGATGTGTGAC

hH2A FP: CTCGCGCCAAAGCCAAGACCCGCTCTTCTC

hH2A RP: TGGATGTTGGGCAGGACACCACCCTGAGCG

hBRCA1 FP: CATCCCTGGTTCCTTGAGGGGTGATTTGTAAC

hBRCA1 RP: ACAACCTCAAAAGACGTCTGTCTACATTGAAT

hPUS10 FP: CAGCTAAAAGAAGCCTACAAATGGATAAC

hPUS10 RP: GCTTTCATAACTGCCATTCTAGTGAATACAGA

hDLG3 FP: CGAAACGTAAAAAGAGTTTCCGCCTCTCTCG

hDLG3 RP: CCCAGGATGATCACAGGCCTTGCATAGTG

hP21 FP: TCAGAGGAGGCGCCATGT

hP21RP: TGTCCACTGGGCCGAAGA

hCCNE1 FP: AGACGGGGAGCTCAAAACTG

hCCNE1 RP: TCGTCCACCAGGGCACCAT

Cacnb3 FP: GAAAGAGGGCGGAGACATCGCCTTCATC

Cacnb3 rp: CTTGGCCAGCGAGAGGTCGGCCGTGAC

Fanca FP: CGCAAGCTGCATGCTCTGGAGCACAAGG

Fanca RP: CTCCTTCTGCCGGCAGCCTCTGTGTCCCT

Dtnb FP: CACGCTGGACCACAGCACGGAGATCAGCG

Dtnb RP: GCTGCGGAAAACAGGTACGGACTGCATGC

Stra6 FP: GGTGAGGGCGGGGATCAACACAGATGTC

Stra6 RP: GCAGCCCCTCGGTGTAGTGCTTGAAGATTG

Ttc3 FP: GAGGGCCATTGTTTTGAAGAACACCTGG

Ttc3 RP: GTGATGTCCTAAAAGCAGGTGCTGATGAG

Tpcn1 FP: GCAGCTGAAGGGGACGCAGGACCACAATG

Tpcn1 RP: TTAACATCCTTGTGAATTCCAAGGCCTTCC

Daam1 FP: CACCTCTGGAAAACTTTAATATCAAGAACG

Daam1 RP: CATTTCTTCCTTCTCTTGGGTCTTAGCATC

Add3 FP: CCACCAGCTCCTCCTAACCCATTCAGC

Add3 RP: GGTGAGCCATCAGGCGACAGGACCTCTTC

Arnt2 FP: GGAGGACTGAATGTCATTCACTCCAGGCACATG

Arnt2 RP: CCCAGTACCCAACCTACCCGCTGGTGTTCACG

Ift122 FP1: GGCACAACGACTCTATACAGTGTGTCTCC

Ift122 RP: CTTGAAGGGTTCCAGCAGATGGACCATA

Ofd1 FP1: GTAGCTATTGTTTATTGCTAGTTTAAG

Ofd1 RP1: GATTACACATGACTTCCTCCAGCCCTC

Lars2 FP1: GCCACTGCAAGACTTGCCTGTGATTTTGCC

Lars2 RP1: GTACAACGAATGCTCACCTGTGCTTCACC

*Nfkb1* minigene reporters

*Nfkb1* genomic regions (Fragments 1, 2 and 3) were synthesized as G-block fragments by IDT DNA and cloned in place of exon 2 in the pRHCglo splicing reporter. G-block fragment sequences are shown.

Fragment 1: cGTGgtcgacAACCCACCTACCTGTCTATGAACATCTGTGGGGGAAAAGTCCCCAAATCCTTCCCAAACTCCGCCATTTTCTTCCTCTTCATAAAACCGAATCTGGATGTCATCTGCAAACGGGCGTATTCAGGCATGTGATTGCAATGCGTGTAATGGTGTTGAAGAGCGCTGTCTTTTTGAGACTCCCACAGCAAAAGGAGAACACCCCTGGTCCGCTGTTTCCAGAGAACTCTGACAGCCACACCCTACATCTAtCtaGaTAag

Fragment 2: cGTGgtcgacACTATTACTGTGGTGGCTCATGTCATCAGCTGGGAGACCCTGGTCATGTAACAATCAGCCAGAGACACCAGCTCATCAGGACAGCATCAGCTTCAGCCCTTTAGACTTCTCAGCCTCTATGTCAGAGTACTGGAAGAGCCATAAACATAGCATTGCTGTATGGATGTCAAAGAGACTTATGACGGTTTCTTTGTTAATATGTGGAAACTGAGCACAACCCACCTACCTGTCTATGAACATCTGTGGGGGAAAAGTCCCCAAATCCTTCCCAAACTCCGCCATTTTCTTCCTCTTCATAAAACCGAATCTGGATGTCATCTGCAAACGGGCGTATTCAGGCATGTGATTGCAATGCGTGTAATGGTGTTGAAGAGCGCTGTCTTTTTGAGACTCCCACAGCAAAAGGAGAACACCCCTGGTCCGCTGTTTCCAGAGAACTCTGACAGCCACACCCTACATCTAtCtaGaTAag

Fragment 3: cGTGgtcgacACTATTACTGTGGTGGCTCATGTCATCAGCTGGGAGACCCTGGTCATGTAACCAATCAGCCAGAGACACCAGCTCATCAGGACAGCATCAGCTTCAGCCCTTTAGACTTCTCAGCCTCTATGTCAGAGTACTGGAAGAGCCATAAACATAGCATTGCTGTATGGATGTCAAAGAGACTTATGACGGTTTCTTTGTTAATATGTGGAAACTGAGCACAACCCACCTACCTGTCTATGAACATCTGTGGGGGAAAAGTCCCCAAATCCTTCCCAAACTCCGCCATTTTCTTCCTCTTCATAAAACCGAATCTGGATGTCATCTGCAAACGGGCGTATTCAGGCATGTGATTGCAATGCGTGTAATGGTGTTGAAGAGCGCTGTCTTTTTGAGACTCCCACAGCAAAAGGAGAACACCCCTGGTCCGCTGTTTCCAGAGAACTCTGACAGCCACACCCTACATCTAAGACCAGGTGTGTAtCtaGaTAag

MS2-minigene reporter splicing assay

The pMS2-GFP and pSL-MS2-12X plasmids were obtained from Addgene (27121 and 27119, respectively) [[8](#_ENREF_8),[9](#_ENREF_9)]. Full length TBX3 was cloned into the MS2-GFP vector for production of the MS2-TBX3 fusion protein. MS2-12X was cloned into the BamH1 site of pRHCglo vector to create the MS2-vector minigene splicing reporter. pMS2-TBX3 and MS2-vector were co-transfected into HEK293 cells with Lipofectamine 2000 as per the manufacturer’s protocol. 48hrs post-transfection, RNA was purified and cDNA was synthesized. cDNA was amplified with minigene specific primers and PCR products were analysed on 6% native PAGE gel.

RNA interference of *TBX3* expression

HEK-293 or MEF cells were transfected with control siRNAs (Sense; 5’ CAGCGACUAAACACAUCA-3’ Antisense; 5’-UUGAUGUGUUUAGUCGCUGTT-3’) or TBX3 specific siRNA A (Sense: GACCAU GGA GCCCGAAGAA; Antisense: UUCUUCGGGCUC CAU GU), siRNA B (Sense: CAGCUCACCCUGCAGUCC A, Antisense: UGGACUGCAGGGUGAGCUG), DDx3 and hnRNPU (Santa Cruz) using lipofectamine plus transfection reagent (Invitrogen) as per manufacturer's instructions. Cells were harvested 48 hrs post-transfection.

Retroviral transduction and selection of stably transformed cells

Primers were annealed and cloned directly into the pGFP-B-RS (Origen) vector. shRNA against luciferase served as a negative control. High-titer retrovirus was produced by transfection of TBX3 shRNA retroviral construct along with gag/pol and VSVG encoding plasmids into HEK 293 EBNA cells by Lipofectamine 2000 reagent as per the manufactures protocol. Virus containing supernatant was collected after 48 hrs, filtered through 0.45 µm filters (Fisher 09-720-4) and virus-containing filtrate used for transduction. HEK293 cells were incubated with complete medium containing polybrene (8mM) and 500 μl of TBX3 shRNA encoding retrovirus medium for the stable integration and knock down of endogenous TBX3. 24 hrs post infection, cells were split to lower densities and blasticidin antibiotic selection applied for 2–3 days. Single stably integrated colonies were picked from the antibiotic selected culture plate and analyzed for knock down efficiency of TBX3 by western analysis using Tbx3 (SC-17871) antibody.

shRNA sequences:

TBX3 A FP: CCGG GACCATGGAGCCCGAAGAA ttcaagaga TTCTTCGGGCTCCATGGTC TTTTTG

TBX3 A RP: AATTCAAAAA GACCATGGAGCCCGAAGAA tctcttgaa TTCTTCGGGCTCCATGGTC

TBX3 B FP: CCGG CAGCTCACCCTGCAGTCCA ttcaagaga TGGACTGCAGGGTGAGCTG TTTTTG

TBX3 B RP: AATTCAAAAA CAGCTCACCCTGCAGTCCA tctcttgaa TGGACTGCAGGGTGAGCTG

RNA immunoprecipitation (RIP) and Crosslinked RIP

For RNA Immunoprecipitation, cells or e10.5 wild type or Tbx3-KO embryos were lysed in NP-40 lysis buffer (50mM Tris HCl, ph7.4, 150 mM NaCl, 1% NP-40 and Protease inhibitor cocktail) and cleared lysate was immunoprecipitated with anti-TBX3, Anti-PABP, Anti-Ddx17, Anti-Ddx3, Anti-hnRNP K, Anti-hnRNP U, Anti-hnRNP C1/C2, mIgG and R-IgG antibodies. Dynabead purified immune complexes were subjected to RNA isolation by TRI reagent (Sigma). For the Crosslinked RIP, Cells were crosslinked for 10 min at room temperature by adding formaldehyde directly to the culture medium at a final concentration of 1%. Immunoprecipitations were carried out as described above. Purified Immune complexes were subjected to Proteinase K digestion at 37°C for 1 hr. Immunoprecipitated RNAs were purified with TRI reagent as per the manufacturer’s protocol. RNA was reverse transcribed by SuperScript III reverse Transcriptase (Invitrogen) reagent. cDNA was used as a template in PCR amplifications with gene specific primers. RIP was performed using rabbit anti-TBX3 polyclonal antibody.

Mice

Lines were maintained on a 50%FVBNS; 25% C57BL/6; 25% SV129 background. Experiments were conducted in compliance with IACUC and AALAC standards. The *Tbx3* conditional allele employed previously described in [[2](#_ENREF_2),[10](#_ENREF_10)]. *Tbx3* conditional limb ablation was obtained with PrxCre [[11](#_ENREF_11)].

MEF Isolation.

MEFs were derived from e13.5 embryos. All steps were performed under aseptic conditions. Pregnant female mice were euthanized and e13.5 embryos were isolated, washed in sterile PBS at room temperature and transferred into 15ml sterile tubes containing 1 ml of 50% trypsin in DMEM. Embryos were minced using fine scissors followed by gentle pipetting with 1ml pipette tips. Embryos were dispersed into cell suspensions within 5 mins and cell suspension plated into 10cm dished in 10ml of DMEM supplemented with 5% FBS, penicillin/streptomycin and incubated for 8hrs in CO2 incubator. Next day, culture medium was replaced with fresh medium and repeated for two days. Transfections were performed in MEFs with X-tremeGENE HP DNA transfection Reagent (Roche) as per the manufacturer’s recommended protocol.

RNA-Seq analysis of embryonic day 10.5 limb buds

Total RNA was isolated from microdissected anterior and posterior segments of e10.5 wild type and *Tbx3;Prx1Cre* conditional mutant forelimb buds using the miRNeasy Kit (Qiagen) according to the manufacturer's protocol. Two biological replicates of each tissue and genotype (2 each of control anterior, control posterior, mutant anterior and mutant posterior) were obtained. RNA purity was assessed using the ND-1000 Nanodrop. RNA was chemically fragmented followed by reverse transcription and ligation of sequencing adapters. Illumina sequencing of the libraries generated 279 million 50 nt single end reads (28-42 million reads per library) with a median base quality of 36. These reads were aligned to the reference genome of *Mus musculus* (mm9) downloaded from the UCSC Genome Browser [[12](#_ENREF_12)] using Tophat [[13](#_ENREF_13)], with default parameters. 261 million reads (94%) were mapped.

Identifying differential Alternative-Splicing events in *Tbx3*mutant limb buds

To identify alternative splicing (AS) events that are differentially regulated between control and mutant  limb buds, we ran USeq software package [[14](#_ENREF_14)] over the RNA-seq datasets of control and mutant segments. For every gene, USeq counts the number of reads aligned to each exon versus the whole gene in cases and controls, and uses Pearson’s Chi-squared test to calculate the significance of differential alternative splicing. In our study, we used “AltSpliceChiSqrPval” values > 13 to identify statistically significant AS events, which corresponds to a p-value of 0.05. Within these genes, the exon with the highest differential alternative splicing signal (reported as “SpliceMaxExon” in USeq output) was used for subsequent analyses, including MEME.

MEME Motif analysis

For pair-wise library comparison of anterior control and mutant mRNA-Seq, a multi fasta file was created containing 1000 bp of flanking genomic sequence on either side of each high-confidence differential alternatively spliced exon.   That fasta file was analyzed with the MEME software package [[15](#_ENREF_15)] to identify DNA motifs enriched in the file with the following parameters: “-dna –mod zoops –nmotifs 10 –minw 5 –maxw 50 –revcomp”.

*In vitro* RNA binding analysis by electrophoretic mobility shift assay (EMSA)

EMSAs were performed as described previously [[16](#_ENREF_16)]. Recombinant TBX3 full-length protein was expressed and purified as a Maltose Binding Protein (MBP) fusion. Full-length Tbx3/MBP and MBP protein alone were expressed in BL21 cells and purified using amylose resin (NEB: E8021S) affinity chromatography. The bacterial cell pellet was lysed in 5 volumes of lysis buffer (200mM Nacl, 20mM Tris HCl pH 7.4, 1mM EDTA, 0.5mM PMSF, 1 tablet of protease inhibitor cocktail (Roche) per 10ml of lysis buffer). Cleared lysate was passed through a pre equilibrated 400-amylose resin column. MBP and MBP-TBX3 proteins were eluted from the amylose resin affinity column using maltose-containing elution buffer (10mM Maltose in lysis buffer). Both the proteins were dialyzed against EMSA binding buffer. Dialyzed proteins were aliquoted and flash frozen in liquid nitrogen and stored at -800C until use. We used 32P-labeled, commercially synthesized RNA oligonucleotides. Radiolabeled fragments were gel purified followed by phenol:chloroform extraction. Binding reactions were performed in a 20-μl total volume containing 10 mM HEPES (pH 7.9), 1 mM dithiothreitol, 50 mM KCl, 2.5 mM MgCl2, 10% glycerol, 0.5 μg salmon sperm DNA, and 1 to 500 ng of recombinant protein. Samples were preincubated at 40C for 5 min. prior to addition of the probe. After 15 min of incubation at 40C temperature, the products of such binding reactions were then resolved by 8% native polyacrylamide gel electrophoresis. The gels were dried under vacuum and exposed to X-ray film.

The antibody supershift assay was performed as described previously [[16](#_ENREF_16)]. Briefly, binding reaction was performed with 5 μg of nuclear extract and radiolabeled PAGE gel purified RNA probe and the complex was incubated at 40C for 15 mins. These RNA-Protein complexes were further incubated with 2 or 5 μg of anti-TBX3 antibody or IgG for 10 mins at 40C and analyzed by native PAGE.

RNA probe sequences tested:

1. AUAUAUAUAUAUAUAUAU
2. GGUGGUGGUGGUGGUGGU
3. AAAAAAAAAAAAAAAAAA
4. GCCGCCGCCGCCGCCGCC
5. AGAGAGAGAGAGAGAGAG
6. UUUUUUUUUUUUUUUUUU
7. GUGUGUGUGUGUGUGUGU
8. ACACACACACACACACAC
9. GCGCGCGCGCGCGCGCGC
10. AAUAAUAAUAAUAAUAAU
11. CUCUCUCUCUCUCUCUCU
12. GUUGUUGUUGUUGUUGUU
13. AUUAUUAUUAUUAUUAUU
14. TBE MOTIF: CCGUGGUGUCACCGUGGUGUCA
15. Mutant: CCGUCCUGUCACCGUCCUGUCA
16. CCAUAAUGUCACCAUAAUGUCA
17. NFKB1 (F1): GUGAUUGCAAUGCGUGUAAUGGUGUUG
18. Nfkb1 (F2): GUGGUGGCUCAUGUCAUCAGCUGGGAG
19. Nfkb1 (F3): GACCAGGUGUGUAU
20. Nfkb1 F2 Mutant: CUCCUCCCUCAUGUCAUCAGCUGGGAG
21. Nfkb1 F3 Mutant: CACCACCUCUCUAU

**Supplemental Information References**

1. Frank DU, Carter KL, Thomas KR, Burr RM, Bakker ML, et al. (2012) Lethal arrhythmias in Tbx3-deficient mice reveal extreme dosage sensitivity of cardiac conduction system function and homeostasis. Proc Natl Acad Sci U S A 109: E154-163.

2. Frank DU, Emechebe U, Thomas KR, Moon AM (2013) Mouse TBX3 mutants suggest novel molecular mechanisms for Ulnar-mammary syndrome. PLoS One 8: e67841.

3. Franklin S, Chen H, Mitchell-Jordan S, Ren S, Wang Y, et al. (2012) Quantitative analysis of the chromatin proteome in disease reveals remodeling principles and identifies high mobility group protein b2 as a regulator of hypertrophic growth. Mol Cell Proteomics 11: M111 014258.

4. Franklin S, Zhang MJ, Chen H, Paulsson AK, Mitchell-Jordan SA, et al. (2011) Specialized compartments of cardiac nuclei exhibit distinct proteomic anatomy. Mol Cell Proteomics 10: M110 000703.

5. Huang da W, Sherman BT, Lempicki RA (2009) Systematic and integrative analysis of large gene lists using DAVID bioinformatics resources. Nat Protoc 4: 44-57.

6. Huang da W, Sherman BT, Tan Q, Kir J, Liu D, et al. (2007) DAVID Bioinformatics Resources: expanded annotation database and novel algorithms to better extract biology from large gene lists. Nucleic Acids Res 35: W169-175.

7. Singh G, Cooper TA (2006) Minigene reporter for identification and analysis of cis elements and trans factors affecting pre-mRNA splicing. Biotechniques 41: 177-181.

8. Fusco D, Accornero N, Lavoie B, Shenoy SM, Blanchard JM, et al. (2003) Single mRNA molecules demonstrate probabilistic movement in living mammalian cells. Curr Biol 13: 161-167.

9. Bertrand E, Chartrand P, Schaefer M, Shenoy SM, Singer RH, et al. (1998) Localization of ASH1 mRNA particles in living yeast. Mol Cell 2: 437-445.

10. Frank DU, Carter KL, Thomas KR, Burr RM, Bakker ML, et al. (2012) Lethal arrhythmias in Tbx3-deficient mice reveal extreme dosage sensitivity of cardiac conduction system function and homeostasis. Proc Natl Acad Sci U S A.

11. Logan M, Martin JF, Nagy A, Lobe C, Olson EN, et al. (2002) Expression of Cre Recombinase in the developing mouse limb bud driven by a Prxl enhancer. Genesis 33: 77-80.

12. Fujita PA, Rhead B, Zweig AS, Hinrichs AS, Karolchik D, et al. (2011) The UCSC Genome Browser database: update 2011. Nucleic Acids Res 39: D876-882.

13. Trapnell C, Pachter L, Salzberg SL (2009) TopHat: discovering splice junctions with RNA-Seq. Bioinformatics 25: 1105-1111.

14. Nix DA, Courdy SJ, Boucher KM (2008) Empirical methods for controlling false positives and estimating confidence in ChIP-Seq peaks. BMC Bioinformatics 9: 523.

15. Bailey TL, Elkan C (1994) Fitting a mixture model by expectation maximization to discover motifs in biopolymers. Proc Int Conf Intell Syst Mol Biol 2: 28-36.

16. Kumar PP, Purbey PK, Ravi DS, Mitra D, Galande S (2005) Displacement of SATB1-bound histone deacetylase 1 corepressor by the human immunodeficiency virus type 1 transactivator induces expression of interleukin-2 and its receptor in T cells. Mol Cell Biol 25: 1620-1633.
